# The mechanism of microglia-mediated immune inflammation in ischemic stroke and the role of natural botanical components in regulating microglia: A review

**DOI:** 10.3389/fimmu.2022.1047550

**Published:** 2023-02-02

**Authors:** Jinsong Zeng, Tingting Bao, Kailin Yang, Xiaofei Zhu, Shanshan Wang, Wang Xiang, Anqi Ge, Liuting Zeng, Jinwen Ge

**Affiliations:** ^1^ The First Hospital of Hunan University of Chinese Medicine, Changsha, Hunan, China; ^2^ Institute of Metabolic Diseases, Guang’anmen Hospital, China Academy of Chinese Medical Sciences, Beijing, China; ^3^ Key Laboratory of Hunan Province for Integrated Traditional Chinese and Western Medicine on Prevention and Treatment of Cardio-Cerebral Diseases, Hunan University of Chinese Medicine, Changsha, China; ^4^ Fudan University, Shanghai, China; ^5^ Department of Rheumatology, The First People's Hospital Changde City, Changde, Hunan, China; ^6^ Hunan Academy of Chinese Medicine, Changsha, Hunan, China

**Keywords:** ischemic stroke, microglia/macrophages, neuroimmune inflammation, natural botanical components, botanicals

## Abstract

Ischemic stroke (IS) is one of the most fatal diseases. Neuroimmunity, inflammation, and oxidative stress play important roles in various complex mechanisms of IS. In particular, the early proinflammatory response resulting from the overactivation of resident microglia and the infiltration of circulating monocytes and macrophages in the brain after cerebral ischemia leads to secondary brain injury. Microglia are innate immune cells in the brain that constantly monitor the brain microenvironment under normal conditions. Once ischemia occurs, microglia are activated to produce dual effects of neurotoxicity and neuroprotection, and the balance of the two effects determines the fate of damaged neurons. The activation of microglia is defined as the classical activation (M1 type) or alternative activation (M2 type). M1 type microglia secrete pro-inflammatory cytokines and neurotoxic mediators to exacerbate neuronal damage, while M2 type microglia promote a repairing anti-inflammatory response. Fine regulation of M1/M2 microglial activation to minimize damage and maximize protection has important therapeutic value. This review focuses on the interaction between M1/M2 microglia and other immune cells involved in the regulation of IS phenotypic characteristics, and the mechanism of natural plant components regulating microglia after IS, providing novel candidate drugs for regulating microglial balance and IS drug development.

## 1 Introduction

Ischemic stroke (IS) is one of the common cerebrovascular diseases, which seriously affects national health due to its high morbidity and lethality (1). It is characterized by the pathological changes of the cerebral arteries or the carotid arteries that innervate the brain, causing cerebral blood circulation disorders, which in turn lead to acute or subacute brain damage. It often causes patients to have varying degrees of language, motor and sensory dysfunction (2). Among them, focal rapid-onset cerebral ischemia-hypoxia (or hemispherical in the case of coma) persists for more than 24 hours or results in death (3). IS constitutes 87% of strokes, including cryptogenic, lacunar and thromboembolic strokes (4). The risk factors for IS include age, smoking, diabetes, high blood pressure and obesity (5). The pathological mechanism is that ischemia and hypoxia in the brain can lead to a series of events including calcium overload, excitatory amino acid neurotoxicity, free radical generation, activation of apoptotic genes, and immune inflammation (6). In fact, recent studies have found that the inflammatory response plays a dual key role in neuroprotection and neurotoxicity in IS (6). Activation of resident cells, such as microglia, astrocytes and endothelial cells promotes both brain regeneration and recovery. It also recruits immune cells that express inflammatory mediators, leading to blood-brain barrier (BBB) disruption, neuronal death, brain edema, and hemorrhagic transformation (6). In this case, clinical treatment options for acute ischemic stroke remain limited. Intravenous injection of tissue-type plasminogen activator (t-PA) restores cerebral perfusion through thrombolysis, which to a certain extent rescues dying cells in the ischemic penumbra (7). However, the treatment time window of 3-4.5 hours is too narrow for practical use of thrombolytics in most parts of the world. Once the therapeutic window is exceeded, the benefits of t-PA are outweighed by its risks, with a dramatic increase in the chance of hemorrhagic transformation (8). Meanwhile, the choice of medical devices for intra-arterial thrombectomy can be used as an alternative to clinical thrombolysis, but it may also cause other complications, so it has great limitations (9). The current study shows that the regulation of immune cells after stroke is the key to regulating inflammation and repairing vascular neural units after stroke (10). As the main body of neuroimmune inflammation in the brain after stroke, microglia play functions such as immune recruitment, regulation, inflammation, phagocytosis, and vascular repair, which in turn become the key to the development of stroke drugs (11). This review focuses on the interaction between classical activation (M1 type) or alternative activation (M2 type) microglia and other immune cells involved in the regulation of IS phenotypic characteristics. Meanwhile, Our previous studies have found that natural compounds and multi-component herbs may treat IS by regulating microglia (12–16); other teams also explored the mechanism by which natural plant active ingredients regulate microglia after IS (17). Therefore, this review also summarizes the natural plant compounds that regulate M1/M2 microglia after IS, in order to provide candidate or lead compounds for the development of drugs that regulate neuroimmune inflammation after IS.

## 2 Pathological mechanisms of immune inflammation in IS

### 2.1 Pathological mechanism of IS

Following an ischemic attack, a series of events involving the central nervous system (CNS) is triggered (18). The pathogenesis of IS begins in the blood vessels, in part due to arterial occlusion leading to hypoxia, reactive oxidative species production, and changes in shear stress on the luminal wall (19, 20). During hypoxia, shear stress on the vascular endothelium due to changes in rheology and blood flow stagnation causes activation of platelets, the complement system and the coagulation cascade, resulting in endothelial cell destruction and microvascular occlusion (21). The combined effect of oxidative stress, inflammatory mediators (such as IL-1β, TNF-α), down-regulated endothelin, and up-regulated leukocyte- or vascular-derived proteases increases BBB permeability (22). Endothelial cell-derived prostaglandins and chemoattractants also promote leukocyte entry into the infarct site (22). The increased surface affinity of leukocytes, the activation of integrin molecules and the up-regulated expression of corresponding ligands on endothelial cells further promote the infiltration of neutrophils, macrophages and other leukocytes. Activated leukocytes produce reactive oxidative species, proteolytic enzymes, cytokines, platelet-activating factor, which promote vasoconstriction, platelet aggregation and further neurotoxicity (19). In the perivascular space, activated macrophages secrete numerous pro-inflammatory cytokines, leading to the release of histamine, proteases and TNF-α, and further reducing the integrity of the BBB (18).

While all of the above processes occur in the vascular and perivascular spaces, ischemia can also affect the brain parenchyma. Following the impact of ischemia, a series of interrelated cytoplasmic and nuclear events, including bioenergetic exhaustion, excitotoxicity, Ca2+ overload, oxidative stress, and inflammatory responses, begin to occur at the damaged site, culminating in neuronal cell death (20). Excitotoxicity and Ca2+ overload are the main factors leading to the early stage of ischemic cell death (23). Glutamate overload results in prolonged activation of AMPA and NMDA ionotropic receptor subtypes, resulting in an enhanced influx of calcium, sodium and water to neurons (23). A large influx of calcium activates protease, lipase and nuclease-mediated catabolic processes (24). Increased calcium influx from glutamate receptor hyperactivation, Ca2+ release from mitochondria, and failure of Ca2+ efflux mechanisms are known to explain the irreversible accumulation of intracellular Ca2+ following excitotoxic stimulation. Meanwhile, oxidative and nitrosative stress are also potent mediators of ischemic injury. Under normal physiological conditions, there is a balance between the production and decomposition of reactive oxygen species (ROS), but IS disrupts this balance and leads to an increase in its production (25). The metabolic activity of ROS and reactive nitrogen species is rapid, and the antioxidant defense capacity of the brain is limited, so the brain is sensitive to damage caused by oxidative and nitrosative stress (26). Damage-associated molecular patterns (DAMPs) released by dying neurons contribute to a new phase of the inflammatory response (27, 28). Among them, heat shock proteins, high mobility group-binding protein 1 (HMGB1), mitochondria-derived N-formyl peptides and peroxidases, activate brain-native immune cells through pattern recognition receptors (29–31). DAMPs lead to an inflammatory environment by stimulating immune cells to produce cytokines, chemokines, adhesion molecules and many immune effector molecules (32–34).

### 2.2 Key events and pathways of immune biological modules involved in the IS pathological progression

After the occurrence of IS, the immune system starts rapidly, participates in all aspects of the occurrence, development and prognosis of stroke, and plays a corresponding role in different stages of stroke (35). Intracerebral inflammation after IS is not limited to the surrounding ischemic foci, but spreads to the whole brain and persists for a long time, continuously affecting the pathophysiological changes of brain tissue after stroke (36). Therefore, understanding the changes and roles of immune responses in different stages of stroke has important guiding significance for further research on neuroprotection and neuroreparation in stroke. Previous studies have shown that in the early stage of IS, various inflammatory cells and factors are involved in the development of inflammation in the brain and aggravate secondary brain injury (21, 37). In the subacute phase, brain injury can remodel the immune system, turning immune system function from activation to suppression, but it leads to an increase in post-stroke infections (21, 38). Moreover, the spread of neuroinflammation in the whole brain after IS can lead to delayed brain tissue changes (39).

#### 2.2.1 Immune activation after IS

After the occurrence of IS, with the occurrence of intravascular hypoxia and changes in hemodynamics, platelets, coagulation and complement systems are activated, and the inflammatory response first occurs in the blood vessels (40). The oxidative stress response and activated complement system caused by hypoxia directly damage the local vascular system, leading to necrosis and dissociation of vascular endothelial cells, destruction of BBB integrity, and exposure of antigens under the vascular endothelium (41). Immune cells in the blood adhere to the vessel wall and upregulate the expression of adhesion factors and chemokines. Innate immune cells such as neutrophils, monocytes, and macrophages are activated, migrate to ischemic sites under the action of chemokines and extravasate into the extravascular space through the damaged BBB (42). Subsequently, macrophages in the ischemic brain tissue are activated to further release inflammatory factors and aggravate the chemotaxis and extravasation of innate immune cells (43). Meanwhile, immune cells such as neutrophils and mast cells at the site of ischemic injury release intracellular MMPs, destroy vascular basement membrane and tight junction proteins, accelerate the destruction of BBB, and lead to an increase in cerebral infarct size (44). Neurons are extremely sensitive to ischemia, hence, ischemia leads to rapid neuronal necrosis. Necrotic neurons release endogenous factors, called DAMPs. DAMPs increase the release of chemokines from immune cells through Toll-like receptors (TLRs) on the surface of immune cells such as microglia, macrophages, dendritic-like cells, and exuding neutrophils (45). This further promotes the chemotaxis of immune cells, activates and amplifies the innate immune response, accelerates vascular destruction and cell death, and ultimately forms a vicious cycle of vascular damage, inflammation, and neuronal death (46). The adaptive immune response is the second phase of the immune response after ischemic stroke, which arises from BBB disruption. Normally immune-isolated central nervous system antigens can contact antigen-presenting cells (APCs) in peripheral blood to induce autoimmune responses. DAMPs can promote the interaction between APCs and receptors to activate adaptive immune responses, which are mediated by effector T cells (47). Effector T cells play a role in the adaptive immune response by recruiting to ischemic areas, traversing the injured BBB, releasing inflammatory cytokines such as interferon gamma (IFN-γ) in the brain parenchyma, and ultimately leading to delayed neurotoxicity (39, 48). The immune response after IS is a self-limiting pathophysiological process that gradually subsides under the combined action of regulatory T cells and B cells, preparing for the structural and functional reconstruction of the later brain injury site. In the process of inflammation resolution, regulatory T cells play a role through IL-10 and transforming growth factor-β (TGF-β) secreted by macrophages in the local tissues, inhibiting helper T cells to further induce inflammation, thereby promoting the repair of residual neurons (49, 50).

#### 2.2.2 Immunosuppression after IS

After IS, while the immune system is activated, immunosuppression will occur at the same time. The systemic immune function is down-regulated within a few hours after cerebral ischemia, the cellular immunity is suppressed, the number of various immune cells such as monocytes, T lymphocytes, B lymphocytes and natural killer cells is decreased, apoptosis is increased, or cell dysfunction occurs (51). Meanwhile, a variety of inflammatory factors, including IL-10, IL-1β, TNF-α, IL-6, etc. are inhibited. This immunosuppressive state is known as stroke-induced immunosuppressive syndrome (SIDS) (52). Its occurrence is related to the activation of the hypothalamic-pituitary-adrenal axis and the sympathetic nervous system caused by stress, and the secretion of adrenocortical hormones and catecholamines increases and plays an immunosuppressive effect (53). Immune activation and immunosuppression after IS are a contradictory unity. The former removes necrotic tissue through inflammatory response to create conditions for nerve repair, and it can also cause secondary nerve damage due to an excessive inflammatory response. The latter can reduce the destruction of neurons by the immune system and play a neuroprotective role, but excessive immunosuppression inevitably increases the chance of infection and worsens clinical prognosis (54).

## 3 Mechanisms of microglia/macrophages in IS

### 3.1 Physiological functions of microglia

Microglia account for approximately 10% of the CNS and are traditionally thought to function as immunocompetent cells of the brain and spinal cord, and undertake sensory functions of injury and infection in tissues (55). Microglia is derived from the primitive c-kit(+) erythroid precursor in the yolk sac, migrates into the brain during early embryonic development before the formation of the BBB, and remains there until the BBB is formed (56). Notably, this is a population of self-maintaining and renewing cells, and peripheral macrophages only contribute to this population in disease states, i.e. when the BBB is damaged (57). Initial studies generally believed that under normal physiological conditions, the microglia in the brain were branched with multiple slender protrusions and were in a resting state. However, recent studies have shown that the microglia never really rests, and the branched microglia constantly patrols the brain, using its motor branch as a sentinel to investigate and scan its nearby microenvironment to detect changes in brain homeostasis (58). Once a threat is identified, microglia rapidly activate to an amoeba-like phenotype with large cell bodies (59). Activated microglia can eliminate cellular debris through phagocytosis on the one hand, and produce a wide range of signaling molecules, including cytokines, neurotransmitters, and extracellular matrix proteins, to regulate neuronal and synaptic activity and their functional plasticity (58). Furthermore, when microglia are involved in the degradation of internalized targets in the phagosome, it may become a major source of ROS. If these internalization targets are too large and not properly processed inside the phagosome, it will result in the release of toxic molecules, including ROS, from the surrounding microglia (60). The normal phagocytosis process is accompanied by the release of several anti-inflammatory cytokines, growth factors and neurotrophic factors, and reduced release of pro-inflammatory cytokines (61). As immune effector cells in the CNS, microglia are continuously active. They monitor the brain microenvironment in real time through the elongation and retraction of branches, modulate neural circuits through specific interactions with neuronal synapses (59, 62), participate in pruning synapses and clear apoptotic cells in time to maintain CNS homeostasis (63–65). They play an important role in most known CNS diseases. A study has monitored the interaction between neurons and fluorescently labeled microglia in transgenic mice by intravital two-photon microscopy imaging (66). They found that microglia made direct contact with neuronal synapses during imaging every 5 minutes for 1 hour. Microglia can rapidly change their phenotype in active response to perturbation of CNS homeostasis and are often activated based on changes in their morphology or cell surface antigen expression (67–69).

### 3.2 Activation and differentiation of microglia/macrophages regulate immune inflammation

#### 3.2.1 Activation of microglia/macrophages after IS

M2-type microglia mainly regulate the repair of brain injury after IS. It mainly promotes the survival and recovery of injured neurons by secreting brain-derived neurotrophic factors, insulin secretion factors and transforming growth factors, and at the same time enhances the ability of neurons to withstand stimulation and damage (70, 71). It produces cytokines IL-10, TGF-β, IL-4, IL-13, IGF-1, etc., which cooperate with the clearance of infiltrating neutrophils, thereby preventing neuronal damage caused by cytotoxic substances (72, 73). Unlike M2-type microglia, which inhibit inflammation, M1-type microglia will produce a large number of pro-inflammatory cytokines IFN-γ, IL-1β, TNF-α, IL-6, etc. to activate the inflammatory cascade, and promote the activation of T and B lymphocytes to regulate immune responses. It can also increase the release of vasoactive factors, causing vasoconstriction and aggravating ischemic cerebral edema (74). M1 type acts on the extracellular matrix through the production of ROS and NO production as well as proteolytic enzymes (MMP9, MMP3), resulting in the decomposition of BBB (39, 75). M1 type also generates reactive oxygen species through nicotinamide adenine dinucleotide phosphate (NADPH) oxidase, which further aggravates the damage of cerebral ischemia (76, 77). Therefore, regulating the homeostasis of M1/M2 type may become an important strategy for the treatment of IS.

#### 3.2.2 Activation and differentiation of M1/M2 type microglia/macrophages

As the first line of defense of the immune system in the brain, microglia are rapidly activated within minutes of the acute phase of ischemic stroke, peak around day 2/3 of activation, and persist for several weeks after the onset of IS (78). At the core of ischemic injury, microglia activation is essentially triggered by excitotoxic signals generated during the ischemic cascade. In the peri-infarct region, the activation of microglia is associated with several innate immune receptors that can be activated by DAMP stimulation (79). For example, purinergic receptors, especially P2X7 and P2Y12, regulate microglial activation and mediate neurotoxicity, and similarly, pharmacological inhibition of P2X7 and P2Y12 reduces brain damage in experimental stroke models (80). Several other innate immune receptors involved in microglial activation include TLR, CD36 scavenger receptor, and receptor for advanced glycation end products (RAGE) (81). In addition to morphological changes, activated microglia also showed altered gene expression patterns, polarizing toward functionally distinct phenotypes: “classically activated” M1 and “alternatively activated” M2.

At present, the main signal pathways that contribute to the polarization of M1/M2 microglia are as follows: (1) IFN-γ secreted by helper T cells 1 (Th1) induces the transformation of microglia into M1 phenotype by activating JAK1/JAK2 and STAT (82). (2) Another pathway to induce M1 activation is triggered by lipopolysaccharide (LPS) or DAMP stimulation of TLR4. Subsequently, an “activation complex” composed of myeloid differentiation factor 88 (Myd88), nuclear factor-KB (NF-KB), p65, p38 and interferon regulatory factor 3 (IRF3) is formed (83). This complex in turn regulates the expression of inflammatory mediators of M1-inducible nitric oxide synthase (iNOS), CD16, CD32, etc. and cell surface markers-histocompatibility complex (MHC-II), CD86, etc. Microglial polarization of the M1 phenotype is characterized by high expression of IL-12, high expression of IL-23 and low expression of IL-10 (84). M2-type microglial replacement activation is usually induced by IL4 or IL-10 and IL-13, and is usually characterized by high expression of IL-12, high expression of IL-23b, and low expression of IL-10. To activate M2-type microglia, IL4 or IL-13 binds to IL4Rx or IL-13Ra1 to activate transcription factors, such as STAT6, peroxisome proliferator-activated receptor gamma (PPARγ), Jumonji domain-containing protein 3 (Jmjd3), and IRF4, respectively. This subsequently causes M2-type microglia to release cytokines such as IL-10, transforming growth factor B (TGFβ), IL-1 receptor agonists, CD302, CD163 and other inflammatory mediators such as platelet-derived growth factor (PDGF), fibronectin 1 and arginase 1 (Arg1), etc. (84).

#### 3.2.3 Activation of microglia and activation, recruitment and polarization of blood-derived macrophages after IS

After IS, intracerebral microglia are rapidly activated within minutes of injury (85), while disruption of BBB integrity allows macrophages to infiltrate the injury site (86, 87). Cerebral ischemia results in dramatic changes in the morphology, density, and function of branched microglia, including processes such as cell body enlargement, debranching, and cell wall thickening. It eventually becomes “amebic”, produces inflammatory proteins, and undergoes changes in proliferation, migration, and phagocytosis (88). Because microglia and blood-migrating macrophages are morphologically indistinguishable and perform similar functions, they are represented as microglia/macrophages in many studies. The current single-cell transcriptomic sequencing also found that the two have similar phenotypes (89). Activated microglia/macrophages have been found to produce a variety of mediators, including iNOS (90), inflammatory cytokines (such as TNF-α, IL-1β, TGF-β, IL-10) (86), nerve growth and trophic factors (such as IGF-1, bFGF, PDGF, BDNF) (91).

Many surface receptors involved in regulating the activation and function of microglia/macrophages have been found: (1) TLRs: TLRs represent a series of pattern-recognition transmembrane receptors that recognize relevant molecular patterns on the surface of pathogens. It is an essential component of the innate immune response of microglia and induces microglia to produce neurotoxic factors that contribute to the microglia response to neuronal damage (92, 93). Studies have found that stimulation of TLR2 and TLR4 activates microglia, produces pro-inflammatory cytokines, and exacerbates brain damage after focal cerebral ischemia (94–99). Knockdown of TLR2 or TLR4 reduces the production of TNF-α, iNOS and cyclooxygenase-2 (COX-2), contributing to smaller cerebral infarct volume (100, 101). (2) Purinergic receptors: Purinergic receptors consist of P1 adenosine receptors and P2ATP receptors (102). Among P1 adenosine receptors, A2A receptors are upregulated in microglia after focal cerebral ischemia and are involved in the control of microglial proliferation and BDNF release induced by LPS stimulation (103). The use of A2A receptor antagonists attenuates ischemia-induced brain damage (104). P2ATP receptors are composed of P2X and P2Y receptors, each containing distinct subunit subtypes (105, 106). Among them, the P2X7 receptor mediates microglia activation after ischemic stroke (107, 108). P2X7 receptor activation induces microglia to release proinflammatory cytokines such as TNF-α, IL-1β, NO, CXCL2 and CCL (109, 110). In addition, P2Y12 is another purinoceptor expressed on microglia, and P2Y12 is down-regulated upon activation of microglia. The accumulation of microglia in the infarcted area was reduced after knockout of the P2Y12 receptor, while attenuating neuronal death following cerebral ischemia in mice (80). (3) CCR2: CCR2 is present in almost all immune cells and highly inflammatory mononuclear MPs (111). However, under normal conditions, CCR2 is poorly expressed in brain microglia (102, 112). CCR2 and its ligand monocyte chemoattractant protein-1 (MCP-1) are upregulated in microglia and migrating macrophages after ischemic stroke (113). Activation of CCR2 enhances cerebral inflammatory responses and significantly increases the volume of cerebral infarcts (114). Deletion of CCR2 in mice reduces blood immune cell recruitment, but does not affect microglia activation after transient MCAO (115). Thus, CCR2 appears to be critical for blood immune cell recruitment, but has little effect on microglia activation after focal cerebral ischemia. (4) Receptor for advanced glycation end products (RAGE): RAGE is another receptor that mediates the activation of microglia/macrophages and plays an important role in the inflammatory response of many diseases (116, 117). In IS patients, RAGE is upregulated in brain and plasma (117, 118). *In vitro* studies have shown that the interaction between RAGE and its ligand high mobility group box 1 (HMGB1) is critical for neuronal death induced by microglia activation (119).

#### 3.2.4 Molecular mechanisms of signaling pathway transduction of M1 and M2 polarization under IS

Microglia/macrophages are activated and polarized upon an ischemic injury, and the degree of polarization changes with pathophysiological conditions (102, 120, 121). Different phenotypes of microglia/macrophages can differentially regulate dying cells after brain injury, possibly aggravating neuronal death or promoting damaged tissue repair (122, 123). Among them, iNOS, IL-1β, IL-6, TNF-α, etc. can be used as molecular markers of M1-type microglia/macrophages. IL-10, IL-4, TGF-β, CD206, Ym1, etc. can be used as molecular markers of M2-type microglia/macrophages. Studies on the progression of IS over time found that on day 1, the M2 phenotype marker Ym1 was highly upregulated in border regions, which induces an M2-type response that provides a protective function for the damaged brain; on day 7, it performs a phagocytic function (124). Further studies showed that M1/M2 microglia participate in different stages of IS. Among them, M2 type mainly appeared in the early stage of cerebral ischemia, appeared 1 to 3 days after ischemia, rose to the highest peak in 3 to 5 days, and returned to the low level before injury on the 14th day. Then it gradually transformed into M1 type on the 3rd day, and maintained a high level for 14 days after ischemia (31). In addition, microglia/macrophages are susceptible to ischemia-induced injury, which may be related to the purinoceptors P2X4 and P2X7, resulting in reduced numbers and suppressed activity of microglia/macrophages in the ischemic core (125, 126). Thus, low levels of microglia/macrophages in the ischemic core and high proportions of M1 and M2 phenotype cells in the peri-infarct area may contribute to the pathological process of ischemic injury.

The signaling molecules associated with M1 phenotype polarization mainly include the following: (1) NF-κB: NF-κB is a traditional transcription factor that is activated by LPS and regulates the expression of most M1 phenotype marker genes. Substantial evidence suggests that the NF-κB signaling cascade adversely affects cerebral ischemia because of its role in regulating pro-inflammatory mediators, including IL-1, IL-2, IL-6, IL-12, TNF-α, iNOS and cyclooxygenase-2 (COX-2) (127, 128). In addition, NF-κB regulates the expression and activation of MMPs, leading to leakage of the BBB and exacerbating the inflammatory response (129, 130). (2) Notch signaling: Notch signaling in response to LPS activation enhances IFN-γ production by co-recruiting p50 and c-Rel (131, 132). Notch signaling exacerbates ischemic brain injury by prolonging NF-κB activation with concomitant persistent inflammation and enhancing microglia/macrophage-induced neurotoxicity (131, 133). (3) Signal transducers and activators of transcription (STAT1 and STAT3): STAT1 and STAT3 can increase the expression of NF-κB/p65 (134). Inhibition of the activation of STAT1 and STAT3 attenuates the inflammatory response induced by cerebral ischemia while improving infarct volume (135, 136). (4) Glycogen synthase kinase-3β (GSK-3β): Cerebral ischemia-induced dephosphorylation and activation of GSK-3β reduces cAMP response element-binding protein (CREB) activity while enhancing NF-κB signaling to initiate pro-inflammatory capacity (137, 138). (5) Prostaglandin E2 (PGE2): PGE2 is the main product of cyclooxygenase and prostaglandin E synthase, and is considered to be a typical pro-inflammatory mediator in the brain. PGE2 activates its downstream signaling pathways through the G protein-coupled E-prostaglandin (EP) receptors EP1-EP4 (139). The EP1 receptor is expressed in microglia, and EP1 deletion inhibits microglial activity and phagocytosis. Although EP2 is expressed in neurons and not in microglia, loss of EP2 results in increased activation of M1-type microglia, suggesting that EP2 mediates the interaction between neurons and microglia (140, 141). (6) mTORC1: mTORC1 is a protein complex downstream of the PI3K-AKt pathway, and is one of the participants in the dysregulation after ischemia and OGD. Maria J et al. (142) showed that blocking mTORC1 can reduce lesion size, improve motor function, significantly reduce the production of pro-inflammatory cytokines and chemokines, and reduce the number of M1-type microglia. Thus, mTORC1 blockade attenuates behavioral deficits and post-stroke inflammation after MCAO by preventing the polarization of microglia towards the M1 type. (7) Related microRNAs: Recent studies have also identified the role of microRNAs (miRNAs) in microglial polarization (143). Current studies have shown that miRNAs involved in the positive regulation of microglial activation and M1 transformation after IS include: miR-689, miR-124, miR-155, miRNAlet-7c-5p, miRNA-200b, MiR-377, etc. These may be related to pro-inflammatory pathways and M1-type polarization (144, 145).

The signaling molecules associated with M2 phenotype polarization mainly include the following: (1) Peroxisome proliferator-activated receptor γ (PPARγ): In the inflammatory response, PPARγ can inhibit the inflammatory response by competitively inhibiting the inflammatory signaling pathway and the generation of inflammatory mediators. Among them, the crosstalk between Notch and NF-κB signaling pathway can inhibit the expression of PPARγ, which will decrease the expression of PPARγ after stroke, thereby aggravating the inflammatory response (146, 147). (2) cAMP response element binding protein (CREB): CREB cooperates with C/EBPβ and amplifies the expression of M2 phenotype-specific genes such as IL-10 and Arg1, promoting tissue repair (148), while the expression of M1 phenotype genes encoding pro-inflammatory molecules is also regulated by C/EBPβ (149). The dual role of C/EBPβ in regulating gene expression of M1 and M2 phenotypes may result from the competition between CREB and NF-κB for binding to C/EBP (148, 150). (3) Interferon regulatory factor-3 (IRF-3): In response to TLR activation, PI3K/Akt signaling initiates phosphorylated IRF-3. Activated IRF-3 translocates into the nucleus and drives polarization of the M2 phenotype by interacting with CREB-binding protein (CBP) (151, 152). (4) Related microRNAs: Current studies have shown that miR-124, miR-711, miR-145, miRNA203 and miRNA27a, which are involved in the positive regulation of microglial activation and M2 transformation after ischemic stroke, may be involved in the regulation of anti-inflammatory pathways and M2-type polarization (144, 145). Among them, miR-146a can not only inhibit the LPS-induced M1-type polarization of microglia, but also promote the M2-type polarization of microglia (145).

### 3.3 Regulation of cellular interactions between microglia/macrophages, neurons, and other immune-inflammatory cells after IS

#### 3.3.1 The effect of the interactive regulation of microglia/macrophages and neurons on brain injury after IS

After IS, a large number of nerve cells die due to reduced blood flow and insufficient supply of glucose and oxygen. Dying neuronal cells release injury-associated ligands and excitotoxic glutamate to promote microglia/macrophage activation (153), thereby exacerbating neuronal damage (154). However, ischemia-induced neuronal injury can release IL-4, which can enhance the expression of IL-4 receptors in microglia/macrophages and promote microglia/macrophage polarization to the M2 phenotype. IL-4-activated PPARγ enhances the phagocytosis of apoptotic neurons by microglia/macrophages (155). The release of glutamate enables neurons to secrete soluble fractalkine (sFKN), which enhances the ability of microglia/macrophages to clear neuronal debris (156). These studies suggest that damaged neurons can promote microglia/macrophage protection to help neurons survive ischemic conditions (154, 157–159). Microglia/macrophages play a beneficial role in tissue remodeling and regeneration after IS by eliminating dead or dying neurons (160). A study of the infiltration of microglia and macrophages in the brain of chimeric mice found that microglia in the brain could phagocytose neuronal debris as early as day 1, and reached a peak on day 2, while infiltrating macrophages began to clear neuronal debris on day 4 after MCAO. They found that microglia in the brain are more sensitive and important in defense against ischemia by eliminating dead neurons (115).

#### 3.3.2 The effect of interaction between microglia/macrophages and astrocytes on brain injury after IS

Microglia and astrocytes play important roles in the innate immune environment of the brain. In two-photon microscopy-based time-lapse imaging recordings, it was found that microglia directly contact astrocytes by extending their branches toward the astrocytes (161). When the brain microenvironment is disrupted, microglia/macrophages and astrocytes play important roles in various pathological states such as IS (162–164). Among them, modulators such as IL-1β, TNF-α, TGF-β, adenosine, ATP and glutamate contribute to functional communication between microglia/macrophages and astrocytes (165–168), which is critical for immune responses in the brain (163). In the CNS, astrocytes mainly secrete cytokines such as IL-6, IL-1β, and IL-10. In addition, astrocytes secrete many chemokines, such as CCL2, CXCL1, CXCL10, and CXCL12, etc., and several chemokines have been found to be involved in microglial activation and polarization, as well as M1 and M2 phenotype switching. *In vitro* studies found that CCL2 released by primary astrocytes contributed to the polarization of M1-type microglia. The proinflammatory mediator lysophosphatidylcholine (LPC) produced by neurons and astrocytes after IS stimulates microglia/macrophages to upregulate the mRNA expression of Mcp-1 and Ccr2, which is involved in mediating the inflammatory response after cerebral ischemia (113). Therefore, there is a complex communication between microglia/macrophages and astrocytes.

#### 3.3.3 The effect of the interaction between microglia/macrophages and T cells on brain injury after IS

After cerebral ischemia, T cells activate and infiltrate into brain tissue, release cytokines and ROS, and induce brain injury by inducing early inflammatory response (169). However, some T cell subtypes have protective effects on brain cells in the early stage of cerebral ischemia. Existing evidence shows that T cells also play an important role in the repair and regeneration of brain tissue in the late stage of stroke (170). T cells include a variety of functional subsets, mainly pro-inflammatory Th1 and Th17 subsets and anti-inflammatory Th2 and Treg subsets. Different T cell subsets play different roles in ischemic brain injury (171). Among them, Th1 can secrete pro-inflammatory cytokines IL-2, IL-12, INF-γ and TNF-α, and play an important role in IS; while Th2 can exert neuroprotective effects by secreting anti-inflammatory cytokines IL-4, IL-10, IL-5 and IL-13 (172). Th17 mainly secretes IL-17, which can promote the occurrence of inflammation (172). IL-10 secreted by Treg is also an important brain protective mediator, which exerts neuroprotective effects mainly by inhibiting the secretion of pro-inflammatory cytokines TNF-α and INF-γ. Treg inhibits secondary infarct enlargement by inhibiting the production of pro-inflammatory cytokines, regulating lymphocyte activation and/or human invasion of ischemic brain tissue (172, 173). After IS, activated microglia and secreted cytokines promote the differentiation of T cells into different functional subsets (6). Among them, M1-type microglia promote the proliferation and differentiation of Th1, while M2-type microglia induce the production of Treg with strong inhibitory function. The interaction between them exerts pro-inflammatory and anti-inflammatory effects, respectively, after stroke, thereby inhibiting the occurrence of the disease or promoting the recovery of the disease (174). Immunofluorescence double-staining of IS brain tissue found that there was a certain interaction between microglia and T cells, indicating that T cells also had a certain regulatory effect on the mutual transformation of M1/M2 microglia (175, 176).

**Figure 1:**
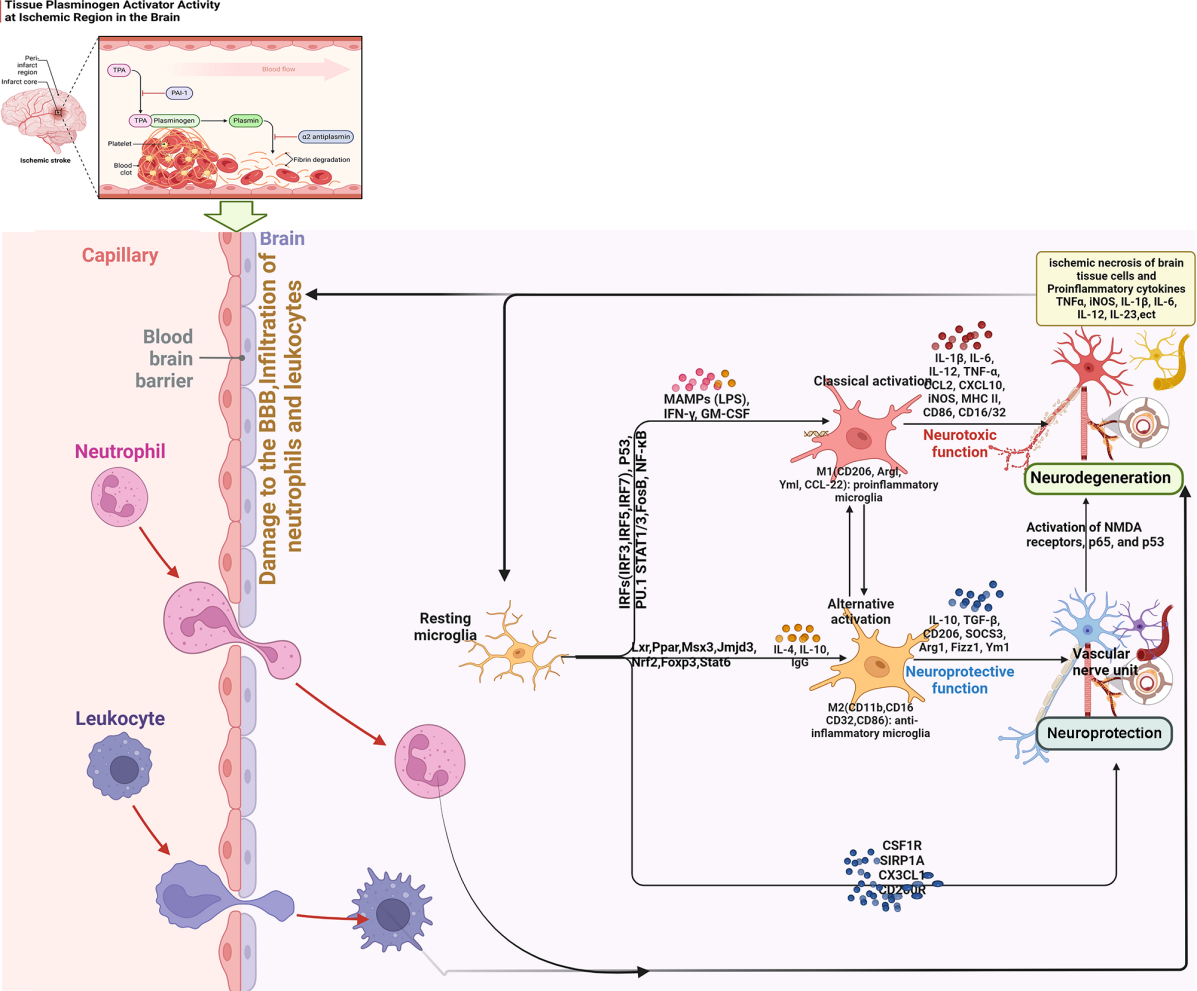
Summary of key inflammatory responses after ischemic stroke (BBB, blood brain barrier; IRF, interferon regulatory factor; STAT, signal transducer and activator of transcription; NF-κB, nuclear factor-κB; IFN, Interferon; NMDA, N-methyl-D-aspartate receptor; MAMPs, Metabolism-related molecular patterns; LPS, Lipopolysaccharide; GM-CSF, granulocyte-macrophage colony stimulating factor).

(1) Interaction between M1-type microglia and Th1/Th17: After ischemia, M1-type microglia can produce pro-inflammatory cytokines leading to BBB disruption (177). Both Th1 and M1 microglia can produce pro-inflammatory cytokines, and iNOS is closely related to inflammatory cells. Cerebral ischemia can induce the up-regulation of iNOS mRNA and protein expression in inflammatory cells, enhance iNOS activity, and promote the production of NO, which can further generate peroxynitrite, thereby aggravating brain damage (178, 179). Studies have shown that Th1 can produce IFN-γ and promote the transformation of microglia into M1 type, thereby aggravating secondary ischemic injury (180). In addition, M1-type microglia can induce Th1 to secrete pro-inflammatory cytokines IL-12 and TNF-α (181–183), and the chemokines (CXCL9, CXCL10) they express can mobilize Th1 to participate in the inflammatory response. Therefore, Th1 and M1-type microglia interact after cerebral ischemia, and can simultaneously promote inflammatory response and aggravate brain injury. IL-17 secreted by Th17 is a powerful pro-inflammatory cytokine that induces the expression of other pro-inflammatory cytokines (such as IL-6 and TNF-α), chemokines, and MMPs, causing inflammatory cell infiltration and tissue destruction. Meanwhile, M1-type microglia induce Th17 differentiation by secreting IL6 and IL-23, which together promote immune response (181–183). Therefore, M1-type microglia and Th17 act as pro-inflammatory effects of brain injury after cerebral ischemia.

(2) Interaction between M2-type microglia and Th2/Treg: After cerebral ischemia, the expression of inflammatory mediators is up-regulated, which induces the accumulation of microglia to the injured area and breaks the dynamic balance between M1 and M2 types. The anti-inflammatory cytokines IL4 and IL-10 secreted by Th2 can further promote the polarization of microglia to M2 type (184, 185). This indicates that there is an interaction between M2-type microglia and Th2 cells, which together play an anti-inflammatory role after cerebral ischemia (186). After cerebral ischemia, Treg inhibits the activation of microglia and reduces the inflammatory response in the brain by secreting IL-10 and TGF-β (187). In addition, Treg cell-derived osteopontin acts through integrin receptors on microglia to enhance the repair activity of microglia, thereby promoting oligodendrogliosis and white matter repair (188). Treg can induce the polarization of microglia to M2 type through the IL-10/GSK3 β/PTEN signaling pathway, thereby reducing the inflammatory injury caused by cerebral hemorrhage (189–191). Treg regulates the expression of other cytokines and inhibits the activation of microglia by releasing IL-10 in the late stage of cerebral infarction. In addition, studies on amyotrophic lateral sclerosis show that Treg can promote the transformation of microglia to M2 type. This suggests that Treg can change its effect from neurotoxicity to neuroprotection without changing the number of microglia (189). After cerebral ischemia, Treg can reduce infarct volume and improve neurological function by reducing T cell infiltration, reducing microglia/monocyte activation, or promoting M2-type polarization of microglia (187). The key inflammatory responses after ischemic stroke are summarized in **Figure 1**.

## 4 Modulatory effects of natural botanical components on microglia-mediated immune inflammation in IS

### 4.1 Natural botanical component monomer

#### 4.1.1 Polyphenols and phenols

(1) Gastrodin: Gastrodin is an organic compound extracted from the dried roots of *Gastrodia elata* Bl (192). Gastrodin has a good sedative and sleeping effect on neurasthenia, insomnia, headache symptoms have eased. *Gastrodia elata* Bl. Is able to treat pain, dizziness, limb numbness, and convulsions. Clinically, *Gastrodia elata* Bl. is generally used to treat vertebrobasilar insufficiency, vestibular neuritis and vertigo (193). Gastrodin is one of the main effective monomer components of *Gastrodia elata* Bl. It is currently widely used in clinical applications, and can exert neuroprotective effects in neurological diseases through multiple pathways such as anti-oxidative stress, anti-neuroinflammatory response, regulation of neurotransmitters, regulation of neural remodeling, anti-apoptosis and anti-autophagy. The study found that gastrodin pretreatment can significantly improve the neurological function of MCAO rats after 72h of reperfusion, and reduce the volume of cerebral infarction and BBB permeability. Gastrodin at 100 mg/kg *in vivo* and 40 μmol/L *in vitro* can inhibit microglia MMP2, MMP9 and AQP4, and increase ZO-1 expression, thereby exerting its protective effect on ischemia-reperfusion injury in MCAO and OGD/R models. In addition, OGD/R and MCAO can significantly increase the expression of SOX4 in microglia *in vitro* and *in vivo*, and pretreatment with gastrodin can inhibit the trend of increasing SOX4. Overexpression of SOX4 could reverse the effects of gastrodin on MMP2, MMP9, AQP4, and ZO-1 in OGD/R microglia, suggesting that gastrodin could regulate MMP2, MMP9, AQP4, and ZO-1 through SOX4 to exert neuroprotective effects (194).

(2) Malibatol A: Malibatol A is a natural resveratrol oligomer purified from the leaves of serrata with antioxidant activity. Yang et al. (195) found that Malibatol A improved mitochondrial dysfunction induced by middle cerebral artery occlusion. Pan et al. (196) found that Malibatol A significantly reduced the infarct size of mice with MCAO and improve neurological function. Weng et al. (197) found that Malibatol A can attenuate OGD/R-induced BV2 cell damage and promote M2 microglial polarization, which may be related to the inhibition of mammalian Ste20-like kinase 1 phosphorylation.

(3) Resveratrol: Resveratrol, a non-flavonoid polyphenolic organic compound, is an antitoxin produced by many plants when stimulated, with a chemical formula of C14H12O3. It can be synthesized in grape leaves and grape skins and is a bioactive component in wine and grape juice (198). *In vitro* and animal experiments have shown that resveratrol has anti-oxidation, anti-inflammatory, inhibition of platelet formation, blood clot adhesion to the vascular wall, anti-cancer and cardiovascular protection (199–201). Resveratrol reduces glial cell activation and prevents delayed neuronal cell death in MCAO rats (202). In addition, resveratrol may protect cranial nerves by reducing the production of inflammatory mediators such as IL-1β, TNF-α and ROS in the ischemic cortex, possibly mediated by attenuating the activation of microglia (203).

(4) 6-Shogaol: 6-shogaol, an active substance isolated from ginger, has a variety of biological activities, including anticancer, anti-inflammatory and antioxidant. For example, 6-shogaol reduced diethylnitrosamine (DEN)-mediated elevation of serum aspartate aminotransferase and alanine aminotransferase and DEN-induced hepatic lipid peroxidation. The induction of Nrf2 and HO-1 by 6-shogaol was also confirmed in mice. 6-shogaol also restores the decreased activity of DEN and the protein expression of liver antioxidant enzymes such as superoxide dismutase, glutathione peroxidase and catalase in mice (204–206). 6-Shogaol also reduces inflammatory biomarkers levels in LPS-activated microglia and neuroinflammation in the brain. It also attenuates iNOS, NO, COX-2, PGE2, TNF-α and IL-1β production by downregulating MAPK (p38, JNK and ERK)/NF-κB signaling. The inhibition of microglial activation and inflammatory mediators by 6-Shogaol contributes to its neuroprotective effect (207).

(5) 6-Paradoll: 6-Paradoll reduces tMCAO-induced cerebral infarction, neurological deficit, and the inflammatory cascade in the ischemic brain, which is mainly mediated by inhibition of microglia/macrophage activation (208).

(6) Honokiol: Honokiol is derived from the bark, root bark and branches of *Magnolia officinalis* Rehd. et Wils. or *M. officinalis* Rehd. et Wils. var. biloba Rehd. et Wils (209). Honokiol has obvious and long-lasting central muscle relaxation, central nervous system inhibition, anti-inflammatory, antibacterial, anti-pathogenic microorganisms, anti-ulcer, antioxidant, anti-aging, anti-tumor, cholesterol-lowering and other pharmacological effects. It is used to treat acute enteritis, bacterial or amoebic dysentery, chronic gastritis, etc. (210, 211). Honokiol inhibits inflammatory biomarkers in the ischemic brain, including NF-κB transcriptional activation, NO, and TNF-α production (212), which are mainly produced by activated glial cells and infiltrating macrophages.

(7) Indole-3-propionic acid: Indole-3-propionic acid treatment not only inhibited glial (astrocyte and microglia) activation in the ischemic brain, but also reduced lipid peroxidation, neuronal DNA damage. Its neuroprotective efficacy is mainly related to the fight against glial cell activation (213).

(8) Paeonol: Paeonol is a monomer extracted from the dried roots of Paeonia suffruticosa Andr. or Cynanchum paniculatum (Bge.) Kitag., which has various pharmacological effects (214). Paeonol has the effect of treating cardiovascular and cerebrovascular diseases, such as lipid-lowering and anti-atherosclerosis, vasodilation and blood pressure lowering, anti-arrhythmia, anti-cerebral ischemia-reperfusion injury and neuroprotection. It also has anti-hepatic injury and liver fibrosis, anti-inflammatory, antibacterial activity, anti-inflammatory activity, antioxidant activity and anti-tumor activity (215–217). Paeonol also decreased IL-1β immunoreactive cell numbers and microglia/macrophage activation in the ischemic brain (218).

(9) Epigallocatechin gallate: Epigallocatechin gallate is a component extracted from green tea. It is the main active and water-soluble component of green tea and is a component of catechins (219). Catechins are mainly divided into four categories: epicatechin, epigallocatechin, epicatechin gallate, epigallocatechin gallate (220). Epigallocatechin gallate has the activity of protecting dopaminergic neurons, inhibiting inflammation, inhibiting oxidative stress, anti-oxidation, protecting nervous system, anti-tumor and protecting cardiovascular and cerebrovascular (221, 222). Epigallocatechin gallate reduces infarct volume by reducing microglia/macrophage activation (223).

(10) Theaflavins: Theaflavins generally refers to tea yellow pigment, is a golden yellow pigment in black tea, is the product of tea fermentation. In biochemistry, tea yellow pigment is a class of polyphenols hydroxyl benzophenone structure of the material, with inhibition of inflammation, anti-oxidative stress and the neuroprotective effect (224–226). Theaflavins reduce infarct and edema volume by reducing microglial inflammatory mediators such as COX-2, iNOS, and ICAM-1 in the damaged brain (227).

(11) Propofol: Propofol reduces infarct volume and improves neurological function by reducing microglia/macrophage CD68 and Emr1 levels and inhibiting proinflammatory cytokines including TNF-α, IL-6, and IL-1β. Inhibition of microglial proinflammatory cytokine production during propofol-mediated MCAO contributes to neuroprotection against IS (228).

(12) Probucol: The neuroprotective effect of probucol is related to its anti-inflammatory effect in microglia. It downregulates NF-κB, MAPKs and AP-1 signaling pathways in LPS-activated microglia to reduce inflammatory mediators such as NO, PGE2, IL-1β and IL-6. It also reduces iNOS, COX-2, IL-1 and IL-6 in the brains of MCAO mice (229).

#### 4.1.2 Anthraquinones

(1) Emodin: Emodin, an active ingredient extracted from the *Rheum palmatum* L., has a wide range of pharmacological properties, including anticancer, hepatoprotective, anti-inflammatory, antioxidant and antibacterial activities (230). Previous studies have shown that (231, 232) emodin has neuroprotective effects, antagonizes CIRI, and prevents the formation of atherosclerotic plaques. However, its neuroprotective mechanism remains unclear. Cai et al. found that emodin may play a role in brain protection by inhibiting the activation of microglia and the release of inflammatory factors mediated by the TLR4/NF-κB pathway (233).

(2) Chrysophanol: Zhang et al. found that Iba-1 positive cells in the cerebral ischemic penumbra of MCAO model rats increased significantly, and were amoeba-shaped or round; the neurological deficit score, the percentage of cerebral infarction and the relative expression of Notch-1, TNF-α and ICAM-1 proteins in the ischemic penumbra were significantly increased (234). However, after chrysophanol intervention, Iba-1-positive cells in the cerebral ischemic penumbra were reduced; the neurological deficit score, the percentage of cerebral infarction and the relative expression of Notch-1, TNF-α and ICAM-1 proteins in the ischemic penumbra were significantly decreased (234). This suggests that chrysophanol has a certain cerebral protective effect on cerebral ischemia injury model rats, and can reduce its nerve damage, and its mechanism may be related to the inhibition of Notch signaling pathway-mediated activation of microglia and the expression of inflammatory factors. In addition, some studies have used a neuroinflammation model of LPS-induced microglial activation, and found that chrysophanol can inhibit LPS-induced microglial inflammatory response and promote the transformation of microglial M1 type to M2 type. The mechanism may be related to down-regulation of TLR4/NF-κB signaling pathway (235).

#### 4.1.3 Terpenes and alkaloids

(1) Astragaloside IV: Astragaloside IV is an organic compound with a chemical formula of C41H68O14. It is a white crystalline powder and is extracted from the herbal medicine *Astragali radix*. The main active ingredients in *Astragali radix* are astragalus polysaccharides, astragalussaponins and isoflavones. Astragaloside IV is mainly used as a quality control standard to evaluate the quality of *Astragali radix* (236, 237). Studies have shown that astragaloside IV can reduce cerebral infarct volume, down-regulate the M1-type microglia/macrophage markers CD86, iNOS, TNF-α, IL-1β and IL-6 mRNA, and up-regulate the M2-type microglia/macrophage markers CD206, Arg-1, YM1/2, IL-10 and TGF-β mRNAs. Astragaloside IV can also reduce the number of CD16/32+/Iba1+ cells and increase the number of CD206+/Iba1+ cells in the ischemic area of the brain. This suggests that astragaloside IV has a protective effect on cerebral ischemia injury in rats, which may be related to promoting the transformation of microglia/macrophages from M1 type to M2 type and inhibiting the inflammatory response (238). In addition, studies have shown that astragaloside IV can inhibit IFN-γ-induced activation of microglia. This is related to inhibiting the activation of STAT1/IκB/NF-κB signaling pathway, reducing the gene expression of IL-1β, TNF-α and iNOS in microglia under inflammatory state, thereby reducing the production of NO and TNF-α (239).

(2) Cycloastragenol: Cycloastragenol is directly extracted from the dried roots of Astragalus membranaceus (Fisch.) Bge.var.mongholicus (Bge.) Hsiao or Astragalus membranaceus (Fisch.) Bge., or obtained by hydrolysis of Astragaloside IV (240, 241). It has oral safety, a wide range of pharmacological effects, anti-oxidation, anti-inflammatory, anti-aging, anti-apoptosis and cardiovascular protection (242, 243). Li et al. found that Cycloastragenol dose-dependently reduced cerebral infarct volume, significantly ameliorated functional deficits, and prevented neuronal cell loss in MCAO mice. Meanwhile, Cycloastragenol significantly reduced the activity of MMP9, prevented the degradation of tight junctions, and subsequently ameliorated the disruption of the BBB. Furthermore, Cycloastragenol significantly upregulated the expression of SIRT1 in the ischemic brain, but did not directly activate its enzymatic activity. Concomitant with SIRT1 upregulation, Cycloastragenol reduces p53 acetylation and Bax to Bcl-2 ratio in the ischemic brain. Cycloastragenol also inhibits NF-κB p65 nuclear translocation. In summary, Cycloastragenol inhibits the expression of proinflammatory cytokines including TNF-α and IL-1β mRNA and inhibits the activation of microglia and astrocytes in the ischemic brain (243).

(3) Triptolide: Tripterygium is derived from the root bark of the traditional Chinese medicine Tripterygium wilfordii, which has anti-inflammatory, antioxidant and anti-cancer effects. Triptolide has been used in the treatment of various diseases, such as tumors [colorectal cancer (244), hepatocellular carcinoma (245)], autoimmune-related diseases [rheumatoid arthritis (246)], obesity (247), etc. Triptolide exerts anti-inflammatory and neuroprotective effects on cerebral ischemia rats through the nuclear factor-KB signaling pathway (248). Jiang et al. found that triptolide reduced neuronal apoptosis and inflammatory factor expression in rats with cerebral ischemia through IL-33/growth-stimulating expression gene 2 protein-mediated polarization of M2 microglia, thereby reducing cerebral infarct volume (249).

(4) Artesunate: Artesunate is a derivative of artemisinin with high water solubility and can pass through the BBB for the treatment of cerebral and other types of severe malaria (250). Artesunate can also maintain a high concentration in the nervous system, showing high efficiency and low toxicity (251–254). Studies have shown that artesunate may exert multiple functions, including anti-inflammatory, immunomodulatory, BBB protection, antibacterial and antitumor effects (253, 254). Studies have shown that the anti-inflammatory effects of artesunate are mediated by NF-κB and inflammatory cytokine inhibition. Lai et al. found that artesunate could alleviate liver fibrosis and inflammation by inhibiting the LPS/TLR4/NF-κB pathway (255). Okorji and Olajide found that artesunate reduced proinflammatory cytokine production by inhibiting the p38 MAPK-NF-κB signaling pathway in activated BV2 microglia (256). Artesunate also exerts a protective effect in CIRI and inhibits the expression of TNF-α and IL-1β (257). Liu et al. found that artesunate significantly improved neurological deficit score and infarct volume, and improved inflammation by reducing neutrophil infiltration, inhibiting microglial activation, and downregulating the expression of TNF-α and IL-1β. In addition, artesunate inhibits nuclear translocation of NF-κB and inhibits protein α proteolysis. In summary, artesunate may protect against inflammatory injury by reducing neutrophil infiltration and microglial activation, inhibiting inflammatory cytokines and inhibiting NF-κB pathway. Therefore, artesunate is a potential IS treatment drug (258).

(5) Neo-Minophagen C: Glycyrrhizin is derived from the glycosides of *Glycyrrhizae radix et rhizoma*, among which Neo-Minophagen C has anti-inflammatory effect, immunomodulatory effect, inhibitory effect on experimental liver cell injury, inhibition of virus proliferation and inactivation of virus (259, 260). Neo-Minophagen C reduces infarct volume and improves motor function and neurological deficits. The neuroprotective effect of Neo-Minophagen C is mediated by reducing neutrophil infiltration and microglial activation after ischemia. Neo-Minophagen C reduces inflammatory mediators and pro-inflammatory cytokines in LPS-activated microglia. Inhibition of microglial activation and inflammatory mediators contributes to the neuroprotective effect of neophage C after cerebral ischemia (261).

(6) Hyperforin: Hyperforin, as an active ingredient of Hypericum perforatum L, is used to alleviate mild to moderate depression and has a significant antidepressant effect (262). Further studies have shown that hyperforin has good anti-inflammatory, anti-tumor and neuroprotective effects (263, 264). Hyperforin reduces infarct size and improves nerve damage by inhibiting inflammatory microglial activation and promoting microglial polarization towards an anti-inflammatory M2 phenotype in the peri-infarct striatum (265).

(7) Ilexonin A: Ilexonin A is a water-soluble compound, 3-succinyl-18-dehydroursolic acid disodium salt, prepared by succinylation of 18-dehydroursolic acid isolated from the rhizome or root bark of Ilex pubescens Hook.et Arn (266). Ilexonin A is an antithrombotic drug whose chemical structure is different from the currently known antiplatelet aggregation drugs. Ilexonin A is effective in the treatment of ischemic cerebrovascular disease, coronary heart disease, central retinal choroiditis, peripheral vascular disease, etc., especially for the treatment of acute ischemic cerebrovascular disease (267, 268). Meanwhile, Ilexonin A reduces inflammatory microglial activation in the ischemic brain. Its neuroprotective effects may be related to neuronal regeneration, inhibition of microglial activation and increased angiogenesis (269).

(8) Huperzine A: Huperzine A is an alkaloid extracted from *Huperzinaserrata (Thumb.)* Trev. It is a potent cholinesterase reversible inhibitor. Its characteristics are similar to neostigmine, but the duration of action is longer than the latter (270, 271). Huperzine A can effectively prevent brain neurasthenia in middle-aged and elderly people, restore brain nerve function, and activate brain nerve transmission substances (272). Huperzine A may inhibit NF-κB activity and pro-inflammatory mediator production in the cerebral cortex and striatum of MCAO rats. It reduces neurological deficits and glial cell activation after ischemic injury mainly through its anti-inflammatory effect in the post-ischemic brain (232). Huperzine A can also down-regulate MAPK signaling, especially JNK and p38, to reduce the level of the inflammatory factor TNF-α. Huperzine A exerts neuroprotection against 2-VO-induced cognitive impairment by promoting an anti-inflammatory response in the brain (273).

(9) Berberine: Berberine, a quaternary ammonium alkaloid isolated from COPTIDIS RHIZOMA, is the main active ingredient in COPTIDIS RHIZOMA for antimicrobial activity. Studies have shown that berberine regulates immune and inflammatory mechanisms (274, 275). Berberine also improves ischemia-induced short-term memory impairment by reducing neuronal apoptosis, microglial activation and oxidative stress. Berberine exerts neuroprotective effects against ischemic injury by increasing the activation of the PI3K/Akt pathway through its phosphorylation in the hippocampus of ischemic gerbils (276).

(10) Sinomenine: Sinomenine is the main active ingredient isolated from *Sinomenium acutum* (Thunb.) Rehd.et Wils (277), which is a kind of morpholine alkaloids, molecular formula is C19H23NO4. It has anti-inflammatory, antihypertensive, analgesic, anti-arrhythmic and other pharmacological activities, and also plays an important role in the treatment of chronic nephritis, anti-oxidation, anti-tumor, detoxification and so on (278, 279). Sinomenine also reduces glial cell activation by inhibiting the NLRP3-ASC-Caspase-1 inflammasome in mixed glial cultures exposed to OGD as well as in MCAO mice. Sinomenine also reduces OGD-induced K phosphorylation in A macrophages *in vitro*. The inhibition of NLRP3 and A-macrophage K activation in activated glial cells by sinomenine is a key cellular mechanism for its neuroprotective effect against stroke (280).

#### 4.1.4 Flavonoids

(1) Icariin: Icariin is the main active ingredient of *Epimedii folium*, which is an 8-prenyl flavonoid glycoside compound (281). Icariin can increase cardiovascular and cerebrovascular blood flow, inhibit inflammation, resist oxidative stress, regulate immunity, promote hematopoietic function, immune function and bone metabolism, and also has the effects of tonifying kidney and strengthening yang, anti-aging and so on (282–284). Tang et al. found that after icariin treatment, the neurological function score and cerebral infarction rate of MCAO model rats were improved, the activation of Iba1 and TLR4 in microglia decreased, the NF-κB p65 protein level decreased, and the content of inflammatory factors IL-1α and TNF-α decreased significantly. This suggests that icariin may play a role in brain protection by regulating the activation of microglia, inhibiting the activation of TLR4 and its downstream NF-κB signaling pathway, and reducing the expression of related inflammatory factors IL-1α and TNF-α (285).

(2) Eupatilin: Eupatilin, a lipophilic flavonoid isolated from ARTEMISIAE ARGYI FOLIUM, is a PPARα agonist with anti-apoptotic, anti-oxidant and anti-inflammatory effects (286–288). Eupatilin may inhibit pro-inflammatory mediators including nitrite, PGE2, TNF-α and IL-6 in activated microglia *in vitro* and *in vivo* to combat focal cerebral ischemia. In the post-ischemic brain of mice challenged with tMCAO, eupatin significantly improved neurological function and reduced cerebral infarction. It also significantly reduced Iba1-immunopositive cells, microglia/macrophage proliferation, NF-κB signaling, IKKα/β phosphorylation, IκBα phosphorylation, and IκBα degradation in the tMCAO-attacked brain. This suggests its powerful effect on counteracting the inflammatory response of microglia/macrophages in the ischemic brain (289).

(3) Heptamethoxyflavonoids: Heptamethoxyflavone protects neuronal cells from ischemia-induced injury by increasing BDNF production, CaMK II phosphorylation, and reducing microglia/macrophage activation (290).

(4) Wogonin: Wogonin is a flavonoid extracted from the root of *Scutellaria baicalensis* Georgi (291). Wogonin has attracted attention because of its various pharmacological activities, including antioxidant activity, anti-inflammatory, immune regulation, and cardiovascular protection. It also has neuroprotective effects during cerebral ischemia (292, 293). Wogonin reduces LPS-induced microglial activation by attenuating the production of inflammatory biomarkers, including iNOS, nitrite, IL-1β, TNF-α and NF-κB in microglia. Furthermore, wogonin treatment down-regulated hippocampal neuronal death by reducing inflammatory mediators such as iNOS and TNF-α after systemic ischemia. It also inhibited the level of microglia-specific isolectin B4 staining, suggesting its role in inhibiting microglial activation (294).

(5) Puerarin: Puerarin is the main active ingredient extracted from Pueraria lobata (Willd.) Ohwi (295). It has the effect of protecting cardiovascular and cerebrovascular and nerve cells, and can dilate blood vessels, lower blood pressure, lower blood sugar, anti-tumor, improve immunity, anti-oxidation, anti-inflammatory and regulate bone metabolism (296–298). Puerarin also reduces ischemia-induced COX-2 expression and reduces cerebral infarction in MCAO rats by inhibiting microglia and astrocyte activation (299).

(6) Quercetin: Quercetin is a naturally occurring phytochemical with good biological activity. It mainly exists in vegetables, fruits, tea and wine in the form of glycosides and has a healthy effect (300, 301). The anti-diabetic, anti-hypertensive, anti-Alzheimer’s disease, anti-arthritis, anti-influenza virus, anti-microbial infection, anti-aging, autophagy and cardiovascular protective effects of quercetin have been extensively studied (302–304). Quercetin may reduce neuroinflammation and apoptosis by reducing the expression of iNOS and caspase-3, which is associated with hippocampal neuroprotection after systemic ischemia in rats (299).

(7) Fisetin: Fisetin is a compound extracted from natural plants with pharmacological effects against different pathological processes (305). The concentration of Fisetin in food is 2 ~ 160μg/g. The content of Fisetin in strawberry, apple and persimmon is high, and fisetin is also abundant in various legume trees and shrubs (306). Studies have shown that Fisetin has a certain therapeutic effect on neurological abnormalities, cardiovascular disease, diabetes, obesity, lung disease, immune disease, cancer and other inflammatory diseases (307, 308). Fisetin may reduce the infiltration of macrophages and dendritic cells into the ischemic hemisphere and reduces immune cell activation in the brain, as evidenced by decreased TNF-α levels. Fisetin significantly downregulates inflammation in LPS-activated microglia and macrophages by reducing NF-κB signaling and reducing TNF-α production. This anti-inflammatory effect of Fisetin is associated with reduced neurotoxicity and neuroprotection by activated microglia and macrophages following ischemic injury (309).

(8) Breviscapine: Breviscapine is the extract of *Erigeron breviscapus* (Vant.) Hand.-Mazz. It is composed of flavonoids, lignans, coumarins, terpenoids, phytosterols and other chemical components (310). Modern pharmacological studies have shown that Breviscapine has a wide range of pharmacological effects, including anti-oxidation, anti-fibrosis, anti-inflammatory, anti-aging, anti-platelet aggregation, reducing blood lipids, increasing blood flow, improving microcirculation, preventing and treating tumors, and anti-brain injury. At present, Breviscapine has been widely used in the treatment of diabetes, cerebral insufficiency, sequelae caused by a cerebral hemorrhage, hyperviscosity, cerebral thrombosis, nephropathy, liver disease, Alzheimer’s disease and other complex diseases (311–313). Breviscapine reduces microglial activation by inhibiting inflammatory biomarkers (such as ROS, NO, and iNOS) in LPS-activated microglia. It also inhibited pro-inflammatory cytokines, especially TNF-α, in the rat brain after ischemia (314). Breviscapine reduces levels of NF-κB, MCP-1 and Notch-1 signaling *in vitro* and *in vivo* in animal models of ischemia. It also reduced microglial migration and adhesion. Breviscapine also inhibits the inflammatory microglia/macrophage phenotype through the Notch pathway and contributes to neuroprotection against ischemia/stroke (315).

(9) Chrysin: Chrysin is a flavonoid found in plant species such as *Oroxylum indicum* (L.) Vent. It is mainly used for intervention in the treatment of hyperlipidemia, cardiovascular and cerebrovascular diseases, anxiety, inflammation, gout, cancer, muscle enhancement, etc. (316–318). Chrysin may reduce the number of activated glial cells, production of pro-inflammatory cytokines, iNOS, COX-2, and NF-κB signaling in the brain after ischemia. Through this anti-inflammatory mechanism, chrysin reduces infarct size and improves neurological deficits (319).

(10) Epicatechin: Epicatechin is a natural plant flavanol compound, chemical formula C15H14O6, with epigallocatechin, catechin gallate, epicatechin gallate, epigallocatechin gallate collectively referred to as catechin compounds (320, 321). Epicatechin and other flavonoids have the effects of anti-oxidation, scavenging free radicals, enhancing metabolism, regulating immunity and anti-tumor (322, 323). Epicatechin reduces oxidative stress by activating the antioxidant Nrf2 pathway, thereby increasing neuronal viability against OGD-mediated injury. In MCAO, epicatechin down-regulates motor dysfunction by down-regulating microglia/macrophage activation (324).

#### 4.1.5 Glycosides

(1) Salidroside: Salidroside is a phenylpropanol glycoside extracted from Rhodiola rosea L., which has good anti-inflammatory and antioxidant effects (325). Han et al. (326) found that salidroside can reduce the size of cerebral infarction in IS rats and improve neurological function and histological changes in rats, which may involve the activation of Nrtf2 pathway and its endogenous antioxidant system. Liu et al. (327) found that in the IS mouse model, salidroside decreased the expression of M1-type markers, increased the expression of M2-type markers, and induced the transformation of microglia from M1 phenotype to M2 phenotype. Salidroside can also inhibit LPS-induced BV2 microglia inflammatory response, mainly by activating PI3K/Akt signaling pathway, promoting Akt phosphorylation, inhibiting NF-κB p50 nuclear transcription, and then inhibiting cytokines. In addition, in a model of neuroinflammation after spinal cord injury, Li et al. found that salidroside can restore motor function in rats, increase the M2/M1 polarization ratio in the spinal cord, reduce the expression of Bax, NF-κB, iNOS and COX-2 mRNA, increase the expression of Bcl-2, p-AMPK, and reduce the expression of p-mTOR.

(2) Forsythin: Forsythin is the dried fruit of *Forsythia suspensa* (Thunb.) Vahl (328, 329). Modern pharmacological studies have shown that Forsythia suspense has antibacterial, anti-inflammatory, antiviral, hepatoprotective, anti-tumor, immune regulation and antioxidant effects (328). Studies have found that *Forsythia suspensa* (Thunb.) Vahl and its constituents have significant effects in improving neurodegenerative diseases and other neuroprotection in the elderly (330). Meanwhile, forsythin may protect neuronal cells in the CA1 region of the hippocampus after ischemia by attenuating glial activation. Forsythin also reduces the levels of pro-inflammatory cytokines such as IL-1β and TNF-α (331).

(3) Ginsenosides: Ginsenoside is a steroid compound, also known as triterpenoid saponins, which mainly exists in *Panax* L (332). The experimental results showed that Ginsenoside inhibited the formation of lipid peroxide in the brain and liver, reduced the content of lipofuscin in the cerebral cortex and liver, and also increased the content of superoxide dismutase and catalase in the blood (333, 334). In addition, some monomer saponins in Ginsenoside such as rb1, rb2, rd, rc, re, rg1, rg2, rh1, etc.can reduce the content of free radicals in the body to varying degrees. Ginsenoside can delay neuronal senescence and reduce memory impairment in the elderly, and has a stable membrane structure and increased protein synthesis, thereby improving the memory ability of the elderly (332, 335). The inhibitory effect of ginsenoside Rd on inflammation was demonstrated by reducing microglial activation and inflammatory biomarkers, including iNOS and COX-2, to exert neuroprotective effects against transient focal ischemia (336). Ginsenoside Rb1 improves neurological deficit and reduces infarct size by reducing microglia activation. Ginsenoside Rb1 treatment reduces the mRNA levels of proinflammatory cytokines such as IL-6, TNF-α by downregulating NF-κB-mediated transcription in the ischemic brain. This suggests that its neuroprotective efficacy is exerted by down-regulating the inflammatory response of activated glia (337).

(4) Kaempferol glycosides: Kaempferol is a flavonoid compound mainly derived from the rhizome of *Kaempferol galanga* L, which is widely found in various fruits, vegetables and beverages (338, 339). It has been widely concerned because of its anti-cancer, anti-cancer, anti-inflammatory, antioxidant, antibacterial, antiviral and other effects (340–342). Kaempferol glycosides, kaempferol-3-O-rutinoside and kaempferol-3-O-glucoside significantly reduced infarct volume, neurological deficits, neuronal and axonal damage in the brains of tMCAO-injured rats. Furthermore, these glycosides inhibit inflammation by reducing transcription mediated by GFAP, OX-42, phosphorylated STAT3, and NF-κB. These glycosides also inhibit macrophage O, iNOS, TNF-α, IL-1β, ICAM-1 and MMP-9 production for neuroprotective effects (343).

(5) Paeoniflorin: Paeoniflorin is derived from the roots of *Paeonia albiflora* Pall, *P. suffersticosa* Andr and *P. delarayi* Franch, and its content is high in *P. lactiflora* (344). Its pharmacological effects of paeoniflorin has significant analgesic, sedative, anticonvulsant effect, antithrombotic effect, anti-platelet aggregation, anti-hyperlipidemia effect, etc. (345, 346). Paeoniflorin ameliorates learning and memory impairment by reducing morphological and structural changes in the CA1 region of brain injury in rats with cerebral hypoperfusion. This neuroprotective efficacy was associated with reductions in inflammatory mediators (eg, NO) and proinflammatory cytokines (eg, IL-1β, TNF-α, and IL-6), and increases in anti-inflammatory cytokines (IL-10 and TGF-β1). Thus, down-regulation of the pro-inflammatory phenotype and increased anti-inflammatory phenotype of activated microglia/macrophages are associated with the neuroprotective efficacy of paeoniflorin (347).

#### 4.1.6 Others

(1) Fingolimod (FTY720): FTY720 is a high affinity agonist for sphingosine 1-phosphate (S1P) receptors and was developed from a sphingosine analogue extracted from *Ophiocordyceps sinensis* (Berk.) G.H. Sung, J.M.Sung, Hywel-Jones & Spatafora as a lead compound; it is approved by the US Food and Drug Administration for the treatment of relapsing-remitting multiple sclerosis (348). Among the four S1P receptor subtypes targeted by FTY720, the current study found that S1P1 and S1P3 are associated with cerebral ischemia (349). The therapeutic mechanism of FTY720 for ischemic stroke is not fully understood. Li et al. (350) found that FTY720 can activate the mammalian target of rapamycin/p70S6 signaling pathway and inhibit neuronal autophagy activity. Many scholars believe that (351, 352), the beneficial effect of FTY720 on IS has nothing to do with direct neuronal protection, but is anti-inflammatory and vascular protection by reducing the invasion of brain lymphocytes. Gaire et al. (349) found that FTY720 inhibits S1P3, thereby inhibiting the transformation of microglia to M1 type. Qin et al. (352) found that FTY720 activates signal transducer and activator of transcription 3 and promotes the polarization of microglia from M1 to M2.

(2) 3-N-butylphthalide (NBP): NBP is a compound isolated from celery seeds, and there are three types of derivatives: L-NBP, D-NBP and DL-NBP (353). L-NBP has been approved for use in China. Among the three derivatives, L-NBP has the strongest biological effect and the best safety (353). NBP has neuroprotective effects on ischemic stroke animal models by inhibiting oxidative damage, neuronal apoptosis and glial cell activation, increasing the level of circulating endothelial progenitor cells (354, 355). Li et al. (356) observed that L-NBP could enhance the M2 polarization of microglia in animal models of cerebral ischemia and inhibit the M1 polarization.

(3) Danshenol bornanyl ester: Danshenol bornanyl ester is a new compound with anti-cerebral ischemia effect, which is designed and synthesized based on the active ingredient of Salvia miltiorrhiza Bge.and the effective structural fragment of borneol by using the principle of modern drug design (357, 358). Danshenol bornanyl ester significantly inhibits NF-κB activity, inhibits the production of pro-inflammatory mediators, and simultaneously promotes the expression of M2 mediators in LPS-stimulated BV2 cells and mouse primary microglia. Danshenol bornanyl ester also exhibits antioxidant activity by enhancing Nrf2 nuclear accumulation and transcriptional activity, increasing HO-1 and NQO1 expression, and inhibiting LPS-induced ROS production in BV2 cells. The aforementioned anti-neuroinflammatory and antioxidant effects could be reversed by Nrf2 knockdown. In addition, Danshenol bornanyl ester improves disease behavior in mice with neuroinflammation induced by systemic LPS administration, significantly reduces infarct volume in rats with transient MCAO (tMCAO), and improves sensorimotor and cognitive function. Danshenol bornanyl ester also restores microglia morphological changes and alters M1/M2 polarization (359).

(4) Arctigenin: Arctigenin is a lignan compound from Arctium lappa L., which can effectively inhibit the release of inflammatory factors. It inhibits the proliferation, migration and angiogenesis of human umbilical vein endothelial cells (HUVECs) induced by high glucose, and plays a protective and anti-oxidative stress role in HUVECs injury (360–362). Arctigenin reduces the activation of microglia by reducing the release of TNF-α and IL-1β in rats with ischemic injury. This anti-inflammatory effect of arctigenin contributes to its neuroprotective effect (363).

(5) Sesamin: Sesamin mainly comes from the roots of *Acanthopanax sessiliflorus* (Rupr.et Maxim.) Seem., the seeds or seed oil of *Sesamum indicum* DC., and the wood of *Paulownia tomentosa* (Thunb.) Steud. Its main pharmacological effects are inhibition of inflammation and anti-oxidative stress (364, 365). Current research found that sesamin may inhibit oxidative stress and reduces cleaved-caspase-3 activation, lipid peroxidation and increases GSH activity. It also inhibited inflammatory mediators such as peroxynitrite, iNOS, COX-2, Iba1, Nox-2 in the brains of MCAO-challenged mice to exert their neuroprotective effects (366).

(6) Edaravone: Edaravone ameliorates cognitive decline and delays neuronal death after focal cerebral ischemia by inhibiting inflammatory biomarkers including iNOS, NO, ROS, IL-1β and TNF-α production. In addition, inhibition of inflammation, oxidative stress, and astrocyte activation are thought to be relevant mechanisms for the neuroprotective effect of edaravone against ischemic injury (314, 367).

(7) Tetramethylpyrazine (Ligustrazine): Ligustrazine is an alkyl pyrazine extracted from *Ligusticum wallichii* (368). It has potential anti-neural and anti-inflammatory activity in rats, can protect vascular endothelial cells, reduce capillary permeability, reduce vascular resistance in anesthetized dogs, and increase blood flow in brain, cerebral arteries and lower limbs (369, 370). Ligustrazine can also reduce the damage of neurons and microvascular endothelial cells and improve neurological signs. It has a short-term improvement effect on complete cerebral ischemia, and has a certain degree of promoting cerebral resuscitation, antagonizing systemic circulation and pulmonary blood pressure after cerebral ischemia (371). Ligustrazine has a strong inhibitory effect on rabbit platelet aggregation induced by ADP, collagen and thrombin *in vitro*, and inhibits the production of platelet malondialdehyde. Its mechanism is to inhibit the phosphorylation of phosphatidylinositol 4-phosphate (PIP) kinase and 20K protein in platelets (87). Ligustrazine can also reduce whole blood viscosity, red blood cell and platelet electrophoresis speed up, reduce fibrinogen, inhibit thrombosis (372). In regulating microglia, ligustrazine can reduce the activation of microglia/macrophages, lymphocyte infiltration and the production of inflammatory mediators in the brain after ischemia. It also reduces inflammatory responses and increases antioxidant/anti-inflammatory responses in microglia/macrophages and post-ischemic neurons *via* Nrf2/HO-1 (373).

(8) Cannabidiol: Cannabidiol is an ingredient extracted from *Cannabis sativa* L (374, 375). At present, its main pharmacological effects are analgesic and anti-inflammatory, inhibition of nerve growth factor-induced mast cell degranulation and neutrophil aggregation to inhibit allergic inflammation, and thus mediate immunosuppression (376, 377). Cannabidiol inhibits hippocampal neurodegeneration, cognitive and memory impairment, glial responses, and white matter damage against BCCAO. It also induces BDNF production in the hippocampus, promoting neurogenesis and dendritic reorganization in BCCAO mice (378).

(9) Ligustolide: Ligustolide is the main active ingredient in the volatile oil of Angelica sinensis (379). The current pharmacological Ligustolide can inhibit the proliferation of vascular smooth muscle cells and cell cycle progression, and inhibit vasoconstriction (380). It also increases vasodilation, antithrombotic and serotoninergic activity, and reduces platelet aggregation (381, 382). Currently, Ligustolide is widely used in the research and treatment of cardiovascular and cerebrovascular diseases and ischemic brain injury (383). Ligustolide can inhibit neuroinflammation and oxidative stress on CIRI damage (384) and reduces cerebral infarct volume and improves neurological function. Ligustolide-induced neuroprotection was accompanied by amelioration of neuropathological changes, decreased activation of microglia and macrophages, infiltration of neutrophils and lymphocytes, and downregulation of inflammatory mediators. This anti-inflammatory effect is controlled by the ERK/NF-κB signaling axis in the ischemic brain. Ligustolide-mediated inhibition of TLR4/Prx6 signaling induces neuroprotection against ischemic stroke (384).

### 4.2 Herb Extracts

#### 4.2.1 Salvia Polyphenolic Acid

Salvia Polyphenolic Acid is an active ingredient extracted from *Salvia miltiorrhiza* Bge. Its main function is to improve the viscous state of blood, with blood circulation, blood stasis, and good clinical tolerance (385, 386). Current studies have shown that its mechanism of action is to inhibit the inflammatory response of endothelial cells, improve energy metabolism, promote vascular endothelial cell migration, and improve ischemia-reperfusion injury (380, 387, 388). Studies have shown that salvianolic acid can reduce the inflammatory factors (such as ICAM-1, IL-1β, IL-18 and TNF-α) in the cerebral cortex of the rat brain MCAO/R model, and reduce the apoptosis of cortical neurons. Salvianolic acid can alleviate the cell damage of oxygen glucose deprivation/reoxygenation (OGD/R)-treated neurons alone and co-cultured with microglia, improve cell viability, and reduce the rate of apoptosis, suggesting that salvianolic acid may reduce the cytotoxicity of microglia to neurons. Salvianolic acid can reduce the expression of NLRP3 in microglia after cerebral ischemia-reperfusion injury (CIRI) in rats, and inhibit the expression of pro-inflammatory factors such as IL-1β and IL-18 in the brain. It can also inhibit the cleavage of the pyroptosis-related protein GSDMD in microglia after CIRI, and reduce the expression of NLRP3, ASC, caspase1, and IL-1β mRNA in microglia. Salvianolic acid can also reduce the number of Iba1 and P2X7 double-labeled microglia in the MCAO/R model rat cerebral cortex, and can reduce the expression of P2X7 protein and mRNA in microglia (194).

#### 4.2.2 Panax notoginseng saponins

Panax notoginseng saponins are the main active ingredients of *Panax notoginseng* (Burkill) F. H. Chen ex C. H., mainly containing ginsenoside Rb1, ginsenoside Rg1, notoginsenoside R1 and other components (389). It can inhibit platelet aggregation in rabbits caused by ADP, and can also dilate cerebral vessels and increase cerebral blood flow (390, 391). Current studies have shown that Panax notoginseng saponins have a wide range of functions in the central nervous system, cardiovascular system, blood system, immune system, anti-fibrosis, anti-aging, anti-tumor, etc. (392–394). Jia et al. found that Panax notoginseng saponins can improve cerebral blood flow, neurological deficits, tissue morphology, and neuronal damage. It can also promote the expression of CD206/Iba-1 in M2-type microglia, and up-regulate the expression of CD206, TGF-β and IL-10 protein; inhibit the expression of CD16/Iba-1 in M1 type microglia cells and down-regulate the expressions of IL-1β, IL-6, TNF-α and iNOS protein. This suggests that Panax notoginseng saponins may promote the transition from M1-type polarization to M2-type in microglia (395).

#### 4.2.3 Omega-3 polyunsaturated fatty acids (n-3 PUFA)

n-3 PUFA is one of the members of the PUFA family, mainly derived from deep-sea fish and some plants, among which eicosapentaenoic acid and docosahexaenoic acid are most involved in the regulation of human physiology. n-3 PUFAs play a role in the prevention and treatment of ischemic stroke (396, 397) can promote neurogenesis, increase peri-infarct vascular formation, improve glial scarring after cerebral ischemia, reduce mitochondrial dysfunction, reduce neuroinflammation, etc. (398, 399). Jiang et al. (400) found that n-3 PUFAs can switch the phenotype of microglia from M1 to M2 in mice with cerebral ischemia, which helps to improve white matter integrity and sensorimotor recovery.

#### 4.2.4 Notoginseng leaf triterpenes

Notoginseng leaf triterpenes, as a valuable drug, have been found to have neuroprotective effects. It can reduce the expression of HMGB1, inhibit the inflammation caused by HMGB1, and inhibit the activation of microglia (IBA1) in hippocampus and cortex, thereby reducing the concentration of inflammatory cytokines including VCAM-1, MMP-9 and MMP-2 and ICAM-1 of IS in a dose-dependent manner. In addition, it can inhibit the activation of MAPKs and NF-κB, thereby ameliorating the neuropathological changes induced by CIRI (401).

#### 4.2.5 Fructus Gardenia Extracts

Fructus Gardenia is a TCM with various pharmacological effects, such as anti-inflammatory, antidepressant, improving cognition and ischemic brain injury. GJ-4 is a natural extract from Fructus Gardenia, GJ-4 can significantly improve memory impairment, cerebral infarction and neurological deficit in MCAO/R-induced vascular dementia (VD) rats. Further studies showed that GJ-4 attenuated VD-induced neuronal damage. In addition, GJ-4 can protect the synapses of VD rats by up-regulating the expression of synaptophysin, postsynaptic density 95 protein (PSD95) and down-regulating the expression of N-methyl-D-aspartate receptor 1 (NMDAR1). Subsequent investigations into the underlying mechanism found that GJ-4 could inhibit neuroinflammatory responses, supported by inhibiting microglia activation and reducing the expression of inflammatory proteins, ultimately exerting neuroprotective effects on VD (402).

**Figure 2:**
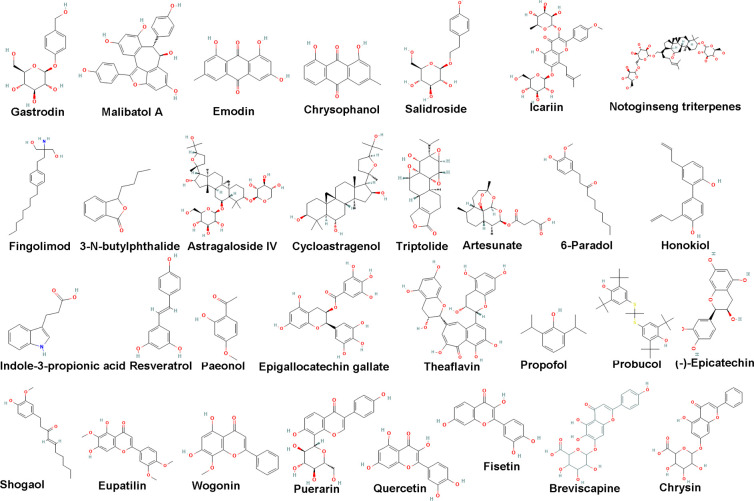
The structure of main natural botanical components.

**Figure 3:**
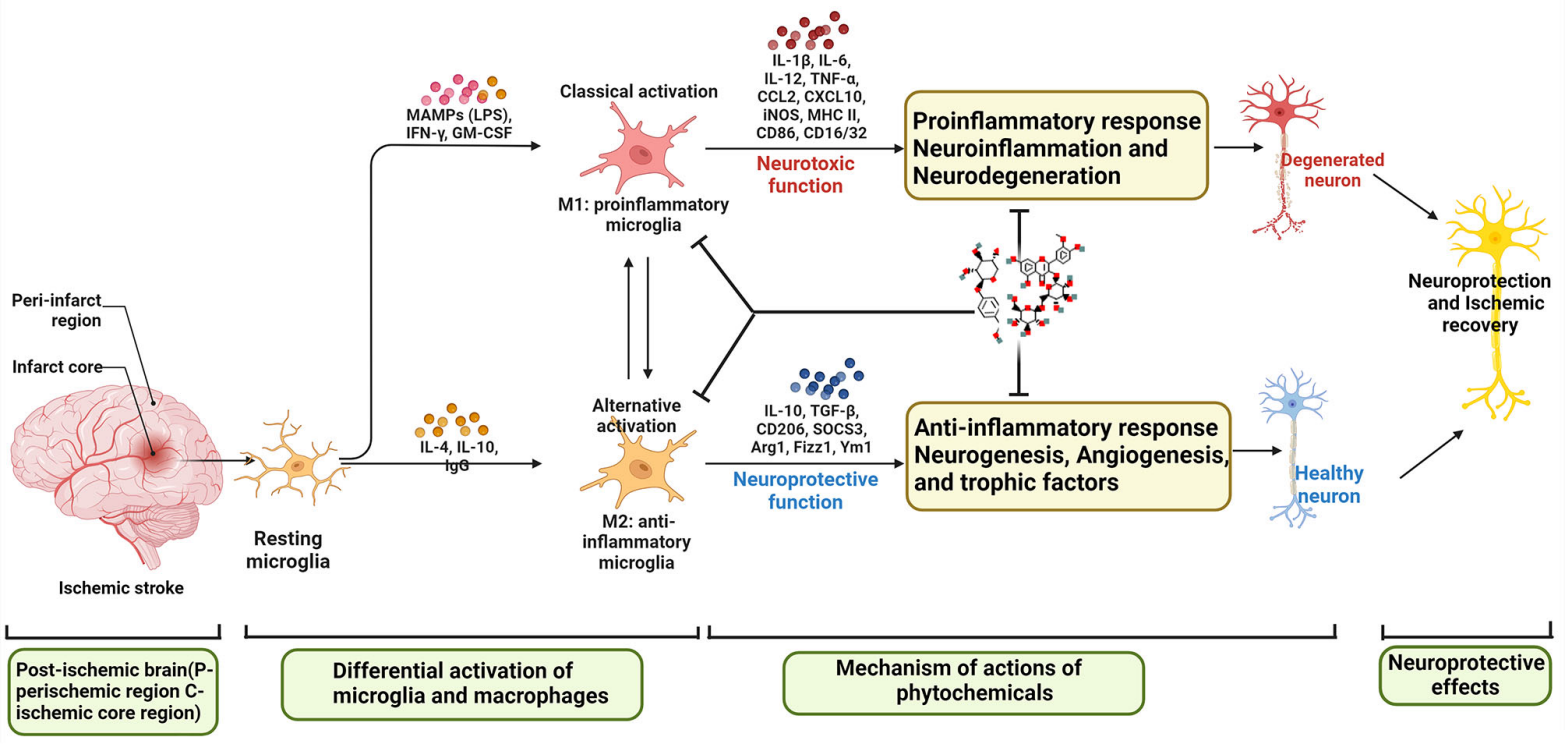
Effects of Natural botanical components on microglia/macrophages after cerebral ischemia (MAMPs, Metabolism-related molecular patterns; LPS, Lipopolysaccharide; GM-CSF, granulocyte-macrophage colony stimulating factor; IL, interleukin; TGF, transforming growth factor).

The structures of those components are shown in **Figure 2**. The effects of natural botanical components on microglia/macrophages after cerebral ischemia are summarized in **Table 1** and **Figure 3**.

**Table 1:** Summary of the role of natural botanical components on IS.

Natural botanical component	Effects	Ischemia type	Category	Reference
Gastrodin	The volume of cerebral infarction and BBB permeability ↓; MMP2, MMP9, AQP4, IL-1β, COX-2 and iNOS↓; ZO-1↑	MCAO/R and OGD/R	Polyphenols and Phenols	(194)
Malibatol A	Promoting M2 microglial polarization	OGD/R	Polyphenols and Phenols	(195–197)
Emodin	MMP-9, TLR4, NF-κB, TNF-α, ICAM-1 ↓	MCAO	Anthraquinones	(230–233)
Chrysophanol	Notch-1, TNF-α and ICAM-1 protein ↓; Iba-1 positive cells↓; promoting the transformation of microglial M1 type to M2 type	MCAO	Anthraquinones	(234, 235)
Salidroside	CD16, CD32, CD11b, iNOS↓; CD206, Arg-1, TGF-β, Ym-1/2 ↑	MCAO	Glycosides	(326, 327)
Icariin	the activation of Iba1 and TLR4 in microglia ↓NF-κB p65 protein ↓; IL-1α and TNF-α ↓	MCAO	Flavonoids	(285)
Salvia Polyphenolic Acid	ICAM-1, IL-1β, IL-18, TNF-α, NLRP3, GSDMD, ASC, CASP1↓; Iba1/P2X7 double-labeled microglia↓	MCAO/R and OGD/R	Polyphenols and Phenols	(194)
Panax notoginseng saponins	CD206, TGF-β and IL-10↑;IL-1β, IL-6, TNF-α and iNOS↓;Promoting the expression of CD206/Iba-1 in M2-type microglia; Inhibiting the expression of CD16/Iba-1 in M1 type microglia cells	MCAO/R	Glycosides	(395)
Notoginseng leaf triterpenes	ICAM-1, VCAM-1, MMP-9, MMP-2, HMGB1 ↓	MCAO/R	Terpenes and Alkaloids	(401)
Omega-3 polyunsaturated fatty acid	Promoting the phenotype of microglia from M1 to M2.	MCAO	Lipids	(398–400)
Fingolimod	promoting the polarization of microglia from M1 to M2	Bilateral carotid artery stenosis	Other	(349–352, 403)
3-N-butylphthalide	Inhibiting oxidative damage, neuronal apoptosis and glial cell activation, increasing the level of circulating endothelial progenitor cells; promoting the M2-type polarization of microglia, inhibiting the M1-type polarization	IS patients; MCAO animal	Other	(356)
Danshenol bornanyl ester	HO-1 and NQO1↑; NF-κB ↓ Altering M1/M2 polarization	MCAO/R	Other	(359)
Astragaloside IV	M1-type microglia/macrophage markers CD86, iNOS, TNF-α, IL-1β and IL-6 mRNA ↓; M2-type microglia/macrophage markers CD206, Arg-1, YM1/2, IL-10 and TGF-β mRNAs ↑; the number of CD16/32+/Iba1+ cells↓; the number of CD206+/Iba1+ cells ↑; IL-1β, TNF-α and iNOS↓	MCAO	Terpenes and Alkaloids	(238, 239)
Cycloastragenol	SIRT1↑; p53, Bax/Bax ratio ↓;TNF-α and IL-1β mRNA↓; Inhibiting NF-κB p65 nuclear translocation; Inhibiting the activation of microglia and astrocytes	MCAO	Terpenes and Alkaloids	(243)
Triptolide	IL-1β, IL-6, TNF-α, IL-33, IL-10 ↓; promoting the M2-type polarization	IS paitents; MCAO/R and OGD/R animal	Terpenes and Alkaloids	(248, 249)
Artesunate	TNF-α and IL-1β ↓; Inhibiting microglial activation	MCAO	Terpenes and Alkaloids	(255–258)
6-Paradol	Iba1 ↓; TNF-α and iNOS↓; Inhibiting microglia/macrophages	MCAO/R	Polyphenols and Phenols	(208)
Honokiol	NF-κB, NO, and TNF-α↓; Inhibiting the M1 phenotypes	2 vessel occlusion/reperfusing	Polyphenols and Phenols	(212)
Indole-3-propionic acid	Iba1 ↓; Inhibiting microglia; Inhibiting the M1 phenotypes	2 vessel occlusion/reperfusing	Polyphenols and Phenols	(213)
Resveratrol	Iba1 ↓; IL-1β and TNF-α↓Inhibiting microglia; Inhibiting the M1 phenotypes	MCAO/R; Bilateral common carotid artery occlusion	Polyphenols and Phenols	(202, 203)
Paeonol	IL-1β↓; Inhibiting microglia; Inhibiting the M1 phenotypes	MCAO/R	Polyphenols and Phenols	(218)
Epigallocatechin gallate	iNOS↓; Inhibiting the M1 phenotypes	MCAO/R	Polyphenols and Phenols	(223)
Theaflavin	COX-2, iNOS↓; Inhibiting the M1 phenotypes	MCAO/R	Polyphenols and Phenols	(227)
Propofol	IL-1β, IL-6, TNF-α↓Inhibiting microglia; Inhibiting the M1 phenotypes	MCAO/R	Polyphenols and Phenols	(314)
Probucol	COX-2, IL-1β, IL-7, iNOS↓; Inhibiting the M1 phenotypes	MCAO/R	Polyphenols and Phenols	(229)
6-Shogaol	Iba1 ↓; Inhibiting microglia	2 vessel occlusion/reperfusing	Polyphenols and Phenols	(207)
Eupatilin	Iba1 ↓; Inhibiting microglia; Inhibiting the M1 phenotypes	MCAO/R	Flavonoids	(289)
Heptamethoxyflavonoids	BDNF↑;Iba1 ↓; Inhibiting microglia; Promoting the M2 phenotypes	2 vessel occlusion	Flavonoids	(290)
Wogonin	TNF-α, iNOS↓; Inhibiting microglia; Inhibiting the M1 phenotypes	MCAO	Flavonoids	(294)
Puerarin	COX-2↓; Inhibiting microglia; Inhibiting the M1 phenotypes	4 vessel occlusion	Flavonoids	(299)
Quercetin	iNOS↓; Inhibiting the M1 phenotypes	Bilateral common carotid artery occlusion	Flavonoids	(299)
Fisetin	TNF-α↓; Inhibiting the M1 phenotypes	MCAO/R	Flavonoids	(309)
Breviscapine	TNF-α, NF-κB, Notch-1, MCP-1, Hes-1 and iNOS↓; Inhibiting microglia; Inhibiting the M1 phenotypes	MCAO	Flavonoids	(314, 315)
Chrysin	Iba1, iNOS, COX-2, CD68, IL-1β, IL-6, IL-12, IL-1α, IL-17a, IFN-γ, and TNF-α ↓; Ym-1↑; Inhibiting microglia; Inhibiting the M1 phenotypes	MCAO/R	Flavonoids	(319)
Epicatechin	Iba1 ↓; Inhibiting the M1 phenotypes	MCAO	Flavonoids	(324)

## 5 Prospect

Microglia play a biphasic regulatory role in ischemic stroke. After IS, microglia were activated, migrated, and exerted pro-inflammatory and anti-inflammatory effects through M1/M2 phenotype polarization, respectively, and at the same time played a protective role by inhibiting M1 phenotype polarization or promoting M2 phenotype polarization. However, the research on induced cell polarization is limited to animal experiments and *in vitro* experiments, and the potential mechanism still needs further research. Apoptosis after IS involves many complex signaling pathways. Regulating the programmed death of neurons in the ischemic penumbra can save dying neurons to the greatest extent and promote the recovery of neural function. In addition, activation of SIPRs, TLRs, NLRPs, PPARs, and P2X7R may be the potential mechanisms for regulating microglia after IS, however, the mechanisms need to be further studied.

Natural plant compounds may have great potential as therapeutic agents to reduce pro-inflammatory responses after cerebral ischemia. As can be seen from the foregoing, most of the current studies describe M1/M2 polarization, but most studies show a mixed signature of M1 or M2 microglial/macrophage phenotypes after cerebral ischemia. An urgent need to address is the need to define the M1 or M2 microglia/macrophage phenotype in experiments with these natural compounds. Furthermore, the markers currently used to differentiate M1 and M2 phenotypes are not microglia or macrophage specific. Therefore, technologies that can separate M1 from M2 microglia/macrophages (such as microfluidics and single-cell transcriptomics to identify novel microglia-specific biomarkers) are needed in the future.

The limitation of this review is that this review focuses on the detailed mechanisms of microglia/macrophages in IS and the mechanisms of natural compound intervention, but lacks a summary of other immune cells such as T cells, B cells, and neutrophils in IS. Since immune cells such as T cells and B cells also play an important role in the pathophysiological process of IS, it is recommended to review the mechanism of these immune cells in IS in the future (39, 169, 172).

In summary, it can be seen that not only single-component natural botanical components can exert a therapeutic effect on IS by regulating microglia, but multi-component natural botanical components also show potential synergistic effects in regulating microglia. This suggests that the combination of natural botanical components to treat multiple inflammatory models (including macrophages and microglia) is a promising direction, and we can see that the combination of natural botanical components will be more effective to achieve the desired therapeutic effect.

## 6 Conclusion

Intervention of natural botanical components and their derivatives in microglia-mediated neuroinflammation in IS is a promising research direction. In the absence of effective neuroprotective drugs, we should pay more attention to the mechanisms of natural botanical components in regulating microglia-mediated neuroinflammatory diseases such as IS. Future research directions are suggested as follows: (1) Differentiate between microglia and macrophages through new techniques (such as single-cell transcriptomics and its derivatives) to more accurately determine the regulatory effects of natural compounds. (2) A more standardized and stable IS model is needed to determine the effects of drugs. (3) Using spatiotemporal omics to map the dynamic continuity state of microglia in natural botanical components intervening in IS. (4) Validate the long-term brain-protective effects of natural botanical components and the mechanisms regulating microglia in rodent and mammalian IS models. (5) Pay attention to the pharmacokinetics, pharmacodynamics, and toxicological properties of natural botanical components. (6) Explore the synergistic effect of natural botanical components in inhibiting neuroinflammation. (7) If all results are favorable, the next step is to conduct clinical trials of potential phytochemicals to investigate their neuroprotective effects on cerebral ischemia/stroke (For example, the team is currently conducting a clinical trial of Naotai Fang in the treatment of cerebral small vessel disease: ChiCTR1900024524).

## Author contributions

TB, and JZ are responsible for the study concept and design. TB, JZ, KY, SW, WX, XZ, AG, LZ, JG are responsible for the data collection, data analysis and interpretation; JZ and KY drafted the paper; JG supervised the study; all authors participated in the analysis and interpretation of data and approved the final paper.

## References

[1] Feske SK. Ischemic stroke. Am J Med (2021) 134(12):1457–64. doi: 10.1016/j.amjmed.2021.07.027 34454905

[2] Herpich F Rincon F. Management of acute ischemic stroke. Crit Care Med (2020) 48(11):1654–63. doi: 10.1097/CCM.0000000000004597 PMC7540624 32947473

[3] Putaala J. Ischemic stroke in young adults. Continuum (MinneapMinn) (2020) 26(2):386–414. doi: 10.1212/CON.0000000000000833 32224758

[4] Mendelson SJ Prabhakaran S. Diagnosis and management of transient ischemic attack and acute ischemic stroke: A review. JAMA (2021) 325(11):1088–98. doi: 10.1001/jama.2020.26867 33724327

[5] GBD 2019 Stroke Collaborators. Global, regional, and national burden of stroke and its risk factors, 1990-2019: a systematic analysis for the global burden of disease study 2019. Lancet Neurol (2021) 20(10):795–820. doi: 10.1016/S1474-4422(21)00252-0 34487721 PMC8443449

[6] Xu S Lu J Shao A Zhang JH Zhang J. Glial cells: Role of the immune response in ischemic stroke. Front Immunol (2020) 11:294. doi: 10.3389/fimmu.2020.00294 32174916 PMC7055422

[7] Kim JS. tPA helpers in the treatment of acute ischemic stroke: Are they ready for clinical use? J Stroke (2019) 21(2):160–74. doi: 10.5853/jos.2019.00584 PMC6549064 31161761

[8] Hollist M Morgan L Cabatbat R Au K Kirmani MF Kirmani BF. Acute stroke management: Overview and recent updates. Aging Dis (2021) 12(4):1000–9. doi: 10.14336/AD.2021.0311 PMC8219501 34221544

[9] Paul S Candelario-Jalil E. Emerging neuroprotective strategies for the treatment of ischemic stroke: An overview of clinical and preclinical studies. Exp Neurol (2021) 335:113518. doi: 10.1016/j.expneurol.2020.113518 33144066 PMC7869696

[10] Qin C Zhou LQ Ma XT Hu ZW Yang S Chen M. Dual functions of microglia in ischemic stroke. Neurosci Bull (2019) 35(5):921–33. doi: 10.1007/s12264-019-00388-3 PMC6754485 31062335

[11] Li LZ Huang YY Yang ZH Zhang SJ Han ZP Luo YM. Potential microglia-based interventions for stroke. CNS NeurosciTher (2020) 26(3):288–96. doi: 10.1111/cns.13291 PMC7052807 32064759

[12] Jiang J Bai Q He C Li Z Song Z Cheng S. Dihuang-Yinzi Decoction Contained Serum Suppresses LPS-induced BV2 Cells Inflammatory Response Through PPARγ/NF-κB Signaling Pathway. World Sci Technology-China Modernization Med (2021) 23(05):1610–6. doi: 10.11842/wst.20210104003

[13] Zhang X Lei C Liu Y Ge J Meng P Zhangyu. The regulatory effect of naotaifang II drug-containing serum on LPS-induced microglial polarization. J Beijing Univ Traditional Chin Med (2020) 43(05):408–13. doi: CNKI:SUN:JZYB.0.2020-05-010

[14] Zuo L. The effect of annao pingchong recipe on inflammatory response after cerebral hemorrhage from the P2X7R/NLRP3 pathway[D]. Hunan Univ Traditional Chin Med (2020). doi: 10.27138/d.cnki.ghuzc.2020.000171

[15] He C Yu W Yang M Li Z Xia X Li P. Baicalin inhibits lipopolysaccharide/interferon γ-induced inflammatory response in BV2 cells *via* TREM2/TLR4/NF-κB signaling pathway. China J Traditional Chin Med (2022) 47(06):1603–10. doi: 10.19540/j.cnki.cjcmm.20211103.401 35347959

[16] Wang XJ Hu R Huang QY Peng QH Yu J. Gynostemma glycosides protect retinal ganglion cells in rats with chronic high intraocular pressure by regulating the STAT3/JAK2 signaling pathway and inhibiting astrocyte and microglia activation. Evid Based Complement Alternat Med (2022) 2022:9963754. doi: 10.1155/2022/9963754.P 35990857 PMC9388231

[17] Chen M Lai X Wang X Ying J Zhang L Zhou B. Long non-coding RNAs and circular RNAs: Insights into microglia and astrocyte mediated neurological diseases. Front Mol Neurosci (2021) 14:745066. doi: 10.3389/fnmol.2021.745066 34675776 PMC8523841

[18] Di Napoli M Elkind MS Godoy DA Singh P Papa F Popa-Wagner A. Role of c-reactive protein in cerebrovascular disease: a critical review. Expert Rev Cardiovasc Ther (2011) 9(12):1565–84. doi: 10.1586/erc.11.159 22103876

[19] Kissa K Herbomel P. Blood stem cells emerge from aortic endothelium by ael type of cell transition. Nature (2010) 464(7285):112–5. doi: 10.1038/nature08761 20154732

[20] Lichanska AM Browne CM Henkel GW Murphy KM Ostrowski MC McKercher SR. Differentiation of the mononuclear phagocyte system during mouse embryogenesis: the role of transcription factor PU. 1 Blood (1999) 94(1):127–38. doi: 10.1182/blood.V94.1.127.413k07_127_138 10381505

[21] Iadecola C Anrather J. The immunology of stroke: from mechanisms to translation. Nat Med (2011) 17(7):796–808. doi: 10.1038/nm.2399 21738161 PMC3137275

[22] Endres M Moro MA Nolte CH Dames C Buckwalter MS Meisel A. Immune pathways in etiology, acute phase, and chronic sequelae of ischemic stroke. Circ Res (2022) 130(8):1167–86. doi: 10.1161/CIRCRESAHA.121.319994 35420915

[23] Liu R Song P Gu X Liang W Sun W Hua Q. Comprehensive landscape of immune infiltration and aberrant pathway activation in ischemic stroke. Front Immunol (2022) 12:766724. doi: 10.3389/fimmu.2021.766724 35140708 PMC8818702

[24] Ankarcrona M Dypbukt JM Bonfoco E Zhivotovsky B Orrenius S Lipton SA. Glutamate-induced neuronal death: a succession of necrosis or apoptosis depending on mitochondrial function. Neuron (1995) 15(4):961–73. doi: 10.1016/0896-6273(95)90186-8 7576644

[25] Rana AK Singh D. Targeting glycogen synthase kinase-3 for oxidative stress and neuroinflammation: Opportunities, challenges and future directions for cerebral stroke management. Neuropharmacology (2018) 139:124–36. doi: 10.1016/j.neuropharm.2018.07.006 30017999

[26] Adibhatla RM Hatcher JF. Lipid oxidation and peroxidation in CNS health and disease: from molecular mechanisms to therapeutic opportunities. Antioxid Redox Signal (2010) 12(1):125–69. doi: 10.1089/ars.2009.2668 19624272

[27] Gomez Perdiguero E Geissmann F. Myb-independent macrophages: a family of cells that develops with their tissue of residence and is involved in its homeostasis. Cold Spring Harb Symp Quant Biol (2013) 78:91–100. doi: 10.1101/sqb.2013.78.020032 24122769

[28] Gomez Perdiguero E Schulz C Geissmann F. Development and homeostasis of "resident" myeloid cells: the case of the microglia. Glia (2013) 61(1):112–20. doi: 10.1002/glia.22393 22847963

[29] Zeyu Z Yuanjian F Cameron L Sheng C. The role of immune inflammation in aneurysmal subarachnoid hemorrhage. Exp Neurol (2021) 336:113535. doi: 10.1016/j.expneurol.2020.113535 33249033

[30] Garcia-Bonilla L Iadecola C. Peroxiredoxin sets the brain on fire after stroke. Nat Med (2012) 18(6):858–9. doi: 10.1038/nm.2797 PMC3955950 22673994

[31] Petrovic-Djergovic D Goonewardena SN Pinsky DJ. Inflammatory disequilibrium in stroke. Circ Res (2016) 119(1):142–58. doi: 10.1161/CIRCRESAHA.116.308022 PMC5138050 27340273

[32] Sánchez KE Rosenberg GA. Shared inflammatory pathology of stroke and COVID-19. Int J Mol Sci (2022) 23(9):5150. doi: 10.3390/ijms23095150 35563537 PMC9101120

[33] DeLong JH Ohashi SN O'Connor KC Sansing LH. Inflammatory responses after ischemic stroke. Semin Immunopathol (2022) 44 (5):625–48. doi: 10.1007/s00281-022-00943-7 35767089

[34] Guo J Wang J Sun W Liu X. The advances of post-stroke depression: 2021 update. J Neurol (2022) 269(3):1236–49. doi: 10.1007/s00415-021-10597-4 34052887

[35] Levard D Buendia I Lanquetin A Glavan M Vivien D Rubio M. Filling the gaps on stroke research: Focus on inflammation and immunity. Brain Behav Immun (2021) 91:649–67. doi: 10.1016/j.bbi.2020.09.025 PMC7531595 33017613

[36] Iadecola C Buckwalter MS Anrather J. Immune responses to stroke: mechanisms, modulation, and therapeutic potential. J Clin Invest (2020) 130(6):2777–88. doi: 10.1172/JCI135530 PMC7260029 32391806

[37] Jayaraj RL Azimullah S Beiram R Jalal FY Rosenberg GA. Neuroinflammation: friend and foe for ischemic stroke. J Neuroinflamm (2019) 16(1):142. doi: 10.1186/s12974-019-1516-2 PMC6617684 31291966

[38] Stanzione R Forte M Cotugno M Bianchi F Marchitti S Rubattu S. Role of DAMPs and of leukocytes infiltration in ischemic stroke: Insights from animal models and translation to the human disease. Cell Mol Neurobiol (2022) 42(3):545–56. doi: 10.1007/s10571-020-00966-4 PMC11441194 32996044

[39] Qiu YM Zhang CL Chen AQ Wang HL Zhou YF Li YN. Immune cells in the BBB disruption after acute ischemic stroke: Targets for immune therapy? Front Immunol (2021) 12:678744. doi: 10.3389/fimmu.2021.678744 34248961 PMC8260997

[40] Shi K Tian DC Li ZG Ducruet AF Lawton MT Shi FD. Global brain inflammation in stroke. Lancet Neurol (2019) 18(11):1058–66. doi: 10.1016/S1474-4422(19)30078-X 31296369

[41] Orellana-Urzúa S Rojas I Líbano L Rodrigo R. Pathophysiology of ischemic stroke: Role of oxidative stress. Curr Pharm Des (2020) 26(34):4246–60. doi: 10.2174/1381612826666200708133912 32640953

[42] Maida CD Norrito RL Daidone M Tuttolomondo A Pinto A. Neuroinflammatory mechanisms in ischemic stroke: Focus on cardioembolic stroke, background, and therapeutic approaches. Int J Mol Sci (2020) 21(18):6454. doi: 10.3390/ijms21186454 32899616 PMC7555650

[43] Lambertsen KL Finsen B Clausen BH. Post-stroke inflammation-target or tool for therapy? Acta Neuropathol (2019) 137(5):693–714. doi: 10.1007/s00401-018-1930-z 30483945 PMC6482288

[44] Mashaqi S Mansour HM Alameddin H Combs D Patel S Estep L. Matrix metalloproteinase-9 as a messenger in the cross talk between obstructive sleep apnea and comorbid systemic hypertension, cardiac remodeling, and ischemic stroke: a literature review. J Clin Sleep Med (2021) 17(3):567–91. doi: 10.5664/jcsm.8928 PMC7927322 33108267

[45] Wang M Pan W Xu Y Zhang J Wan J Jiang H. Microglia-mediated neuroinflammation: A potential target for the treatment of cardiovascular diseases. J Inflammation Res (2022) 15:3083–94. doi: 10.2147/JIR.S350109 PMC9148574 35642214

[46] Couch C Mallah K Borucki DM Bonilha HS Tomlinson S. State of the science in inflammation and stroke recovery: A systematic review. Ann Phys Rehabil Med (2022) 65(2):101546. doi: 10.1016/j.rehab.2021.101546 34098132 PMC9018463

[47] Wicks EE Ran KR Kim JE Xu R Lee RP Jackson CM. The translational potential of microglia and monocyte-derived macrophages in ischemic stroke. Front Immunol (2022) 13:897022. doi: 10.3389/fimmu.2022.897022 35795678 PMC9251541

[48] Muhammad S Chaudhry SR Kahlert UD Niemelä M Hänggi D. Brain immune interactions-novel emerging options to treat acute ischemic brain injury. Cells (2021) 10(9):2429. doi: 10.3390/cells10092429 34572077 PMC8472028

[49] An C Shi Y Li P Hu X Gan Y Stetler RA. Molecular dialogs between the ischemic brain and the peripheral immune system: dualistic roles in injury and repair. Prog Neurobiol (2014) 115:6–24. doi: 10.1016/j.pneurobio.2013.12.002 24374228 PMC4014303

[50] Ziv Y Ron N Butovsky O Landa G Sudai E Greenberg N. Immune cells contribute to the maintenance of neurogenesis and spatial learning abilities in adulthood. Nat Neurosci (2006) 9(2):268–75. doi: 10.1038/nn1629 16415867

[51] Hug A Dalpke A Wieczorek N Giese T Lorenz A Auffarth G. Infarct volume is a major determiner of post-stroke immune cell function and susceptibility to infection. Stroke (2009) 40(10):3226–32. doi: 10.1161/STROKEAHA.109.557967 19661470

[52] Meisel C Schwab JM Prass K Meisel A Dirnagl U. Central nervous system injury-induced immune deficiency syndrome. Nat Rev Neurosci (2005) 6(10):775–86. doi: 10.1038/nrn1765 16163382

[53] Chen C Ai QD Chu SF Zhang Z Chen NH. NK cells in cerebral ischemia. BioMed Pharmacother (2019) 109:547–54. doi: 10.1016/j.biopha.2018.10.103 30399590

[54] Shim R Wong CH. Ischemia, immunosuppression and infection–tackling the predicaments of post-stroke complications. Int J Mol Sci (2016) 17(1):64. doi: 10.3390/ijms17010064 26742037 PMC4730309

[55] Shi AC Rohlwink U Scafidi S Kannan S. Microglial metabolism after pediatric traumatic brain injury - overlooked bystanders or active participants? Front Neurol (2021) 11:626999. doi: 10.3389/fneur.2020.626999 33569038 PMC7868439

[56] Zhou R Qian S Cho WCS Zhou J Jin C Zhong Y. Microbiota-microglia connections in age-related cognitionline. Aging Cell (2022) 21(5):e13599. doi: 10.1111/acel.13599 35349746 PMC9124309

[57] Leng F Edison P. Neuroinflammation and microglial activation in Alzheimer disease: where do we go from here? Nat Rev Neurol (2021) 17(3):157–72. doi: 10.1038/s41582-020-00435-y 33318676

[58] Borst K Dumas AA Prinz M. Microglia: Immune and non-immune functions. Immunity (2021) 54(10):2194–208. doi: 10.1016/j.immuni.2021.09.014 34644556

[59] Cserép C Pósfai B Dénes Á. Shaping neuronal fate: Functional heterogeneity of direct microglia-neuron interactions. Neuron (2021) 109(2):222–40. doi: 10.1016/j.neuron.2020.11.007 33271068

[60] Wink DA Hines HB Cheng RY Switzer CH Flores-Santana W Vitek MP. Nitric oxide and redox mechanisms in the immune response. J Leukoc Biol (2011) 89(6):873–91. doi: 10.1189/jlb.1010550 PMC3100761 21233414

[61] Lukens JR Eyo UB. Microglia and neurodevelopmental disorders. Annu Rev Neurosci (2022) 45:425–45. doi: 10.1146/annurev-neuro-110920-023056 PMC10449242 35436413

[62] Andoh M Koyama R. Microglia regulate synaptic development and plasticity. Dev Neurobiol (2021) 81(5):568–90. doi: 10.1002/dneu.22814 PMC8451802 33583110

[63] Woodburn SC Bollinger JL Wohleb ES. The semantics of microglia activation: neuroinflammation, homeostasis, and stress. J Neuroinflamm (2021) 18(1):258. doi: 10.1186/s12974-021-02309-6 PMC8571840 34742308

[64] Marinelli S Basilico B Marrone MC Ragozzino D. Microglia-neuron crosstalk: Signaling mechanism and control of synaptic transmission. Semin Cell Dev Biol (2019) 94:138–51. doi: 10.1016/j.semcdb.2019.05.017 31112798

[65] Verkhratsky A Sun D Tanaka J. Snapshot of microglial physiological functions. Neurochem Int (2021) 144:104960. doi: 10.1016/j.neuint.2021.104960 33460721

[66] Wake H Moorhouse AJ Jinno S Kohsaka S Nabekura J. Resting microglia directly monitor the functional state of synapses *in vivo* and determine the fate of ischemic terminals. J Neurosci (2009) 29(13):3974–80. doi: 10.1523/JNEUROSCI.4363-08.2009 PMC6665392 19339593

[67] Andoh M Koyama R. Assessing microglial dynamics by live imaging. Front Immunol (2021) 12:617564. doi: 10.3389/fimmu.2021.617564 33763064 PMC7982483

[68] Guo S Wang H Yin Y. Microglia polarization from M1 to M2 in neurodegenerative diseases. Front Aging Neurosci (2022) 14:815347. doi: 10.3389/fnagi.2022.815347 35250543 PMC8888930

[69] Zhang L Cao Y Zhang X Gu X Mao Y Peng B. The origin and repopulation of microglia. Dev Neurobiol (2022) 82(1):112–24. doi: 10.1002/dneu.22862 34874111

[70] Orihuela R McPherson CA Harry GJ. Microglial M1/M2 polarization and metabolic states. Br J Pharmacol (2016) 173(4):649–65. doi: 10.1111/bph.13139 PMC4742299 25800044

[71] Ma D Feng L Deng F Feng JC. Overview of experimental and clinical findings regarding the neuroprotective effects of cerebral ischemic postconditioning. BioMed Res Int (2017) 2017:6891645. doi: 10.1155/2017/6891645 28473987 PMC5394355

[72] Saxena S Kruys V Vamecq J Maze M. The role of microglia in perioperative neuroinflammation and neurocognitive disorders. Front Aging Neurosci (2021) 13:671499. doi: 10.3389/fnagi.2021.671499 34122048 PMC8193130

[73] Kanazawa M Ninomiya I Hatakeyama M Takahashi T Shimohata T. Microglia and Monocytes/Macrophages polarization revealel therapeutic mechanism against stroke. Int J Mol Sci (2017) 18(10):2135. doi: 10.3390/ijms18102135 29027964 PMC5666817

[74] Lanza M Casili G Campolo M Paterniti I Colarossi C Mare M. Immunomodulatory effect of microglia-released cytokines in gliomas. Brain Sci (2021) 11(4):466. doi: 10.3390/brainsci11040466 33917013 PMC8067679

[75] Tian DS Li CY Qin C Murugan M Wu LJ Liu JL. Deficiency in the voltage-gated proton channel Hv1 increases M2 polarization of microglia and attenuates brain damage from photothrombotic ischemic stroke. J Neurochem (2016) 139(1):96–105. doi: 10.1111/jnc.13751 27470181 PMC5037018

[76] Hur J Lee P Kim MJ Kim Y Cho YW. Ischemia-activated microglia induces neuronal injury *via* activation of gp91phox NADPH oxidase. Biochem Biophys Res Commun (2010) 391(3):1526–30. doi: 10.1016/j.bbrc.2009.12.114 20036216

[77] Li QQ Li JY Zhou M Qin ZH Sheng R. Targeting neuroinflammation to treat cerebral ischemia - the role of TIGAR/NADPH axis. Neurochem Int (2021) 148:105081. doi: 10.1016/j.neuint.2021.105081 34082063

[78] Candelario-Jalil E Dijkhuizen RM Magnus T. Neuroinflammation, stroke, blood-brain barrier dysfunction, and imaging modalities. Stroke (2022) 53(5):1473–86. doi: 10.1161/STROKEAHA.122.036946 PMC9038693 35387495

[79] Kwon HS Koh SH. Neuroinflammation in neurodegenerative disorders: the roles of microglia and astrocytes. Transl Neurodegener (2020) 9(1):42. doi: 10.1186/s40035-020-00221-2 33239064 PMC7689983

[80] Webster CM Hokari M McManus A Tang XN Ma H Kacimi R. Microglial P2Y12 deficiency/inhibition protects against brain ischemia. PloS One (2013) 8(8):e70927. doi: 10.1371/journal.pone.0070927 23940669 PMC3733797

[81] Kawabori M Kacimi R Kauppinen T Calosing C Kim JY Hsieh CL. Triggering receptor expressed on myeloid cells 2 (TREM2) deficiency attenuates phagocytic activities of microglia and exacerbates ischemic damage in experimental stroke. J Neurosci (2015) 35(8):3384–96. doi: 10.1523/JNEUROSCI.2620-14.2015 PMC4339351 25716838

[82] Roesch S Rapp C Dettling S Herold-Mende C. When immune cells turn bad-Tumor-Associated Microglia/Macrophages in glioma. Int J Mol Sci (2018) 19(2):436. doi: 10.3390/ijms19020436 29389898 PMC5855658

[83] Chu F Shi M Zheng C Shen D Zhu J Zheng X. The roles of macrophages and microglia in multiple sclerosis and experimental autoimmune encephalomyelitis. J Neuroimmunol (2018) 318:1–7. doi: 10.1016/j.jneuroim.2018.02.015.E 29606295

[84] Dos Santos IRC Dias MNC Gomes-Leal W. Microglial activation and adult neurogenesis after brain stroke. Neural Regener Res (2021) 16(3):456–9. doi: 10.4103/1673-5374.291383 PMC7996005 32985465

[85] Mahmood A Miron VE. Microglia as therapeutic targets for central nervous system remyelination. Curr Opin Pharmacol (2022) 63:102188. doi: 10.1016/j.coph.2022.102188 35219055

[86] Lin X Wang H Chen J Zhao P Wen M Bingwa LA. Nonhuman primate models of ischemic stroke and neurological evaluation after stroke. J Neurosci Methods (2022) 376:109611. doi: 10.1016/j.jneumeth.2022.109611 35487315

[87] Zhu T Wang L Feng Y Sun G Sun X. Classical active ingredients and extracts of Chinese herbal medicines: Pharmacokinetics, pharmacodynamics, and molecular mechanisms for ischemic stroke. Oxid Med Cell Longev (2021) 2021:8868941. doi: 10.1155/2021/8868941 33791075 PMC7984881

[88] Holste KG Xia F Ye F Keep RF Xi G. Mechanisms of neuroinflammation in hydrocephalus after intraventricular hemorrhage: a review. Fluids Barriers CNS (2022) 19(1):28. doi: 10.1186/s12987-022-00324-0 35365172 PMC8973639

[89] Shi X Luo L Wang J Shen H Li Y Mamtilahun M. Stroke subtype-dependent synapse elimination by reactive gliosis in mice. Nat Commun (2021) 12(1):6943. doi: 10.1038/s41467-021-27248-x 34836962 PMC8626497

[90] Dhillon SK Gunn ER Lear BA King VJ Lear CA Wassink G. Cerebral oxygenation and metabolism after hypoxia-ischemia. Front Pediatr (2022) 10:925951. doi: 10.3389/fped.2022.925951 35903161 PMC9314655

[91] Zhang Q Jia M Wang Y Wang Q Wu J. Cell death mechanisms in cerebral ischemia-reperfusion injury. Neurochem Res (2022) 47(12):3525–42. doi: 10.1007/s11064-022-03697-8 35976487

[92] Wang Z Weaver DF. Microglia and microglial-based receptors in the pathogenesis and treatment of alzheimer's disease. Int Immunopharmacol (2022) 110:109070. doi: 10.1016/j.intimp.2022.109070 35978514

[93] Shi Y Holtzman DM. Interplay between innate immunity and Alzheimer disease: APOE and TREM2 in the spotlight. Nat Rev Immunol (2018) 18(12):759–72. doi: 10.1038/s41577-018-0051-1 PMC6425488 30140051

[94] Caso JR Pradillo JM Hurtado O Lorenzo P Moro MA Lizasoain I. Toll-like receptor 4 is involved in brain damage and inflammation after experimental stroke. Circulation (2007) 115(12):1599–608. doi: 10.1161/CIRCULATIONAHA.106.603431 17372179

[95] Kilic U Kilic E Matter CM Bassetti CL Hermann DM. TLR-4 deficiency protects against focal cerebral ischemia and axotomy-induced neurodegeneration. Neurobiol Dis (2008) 31(1):33–40. doi: 10.1016/j.nbd.2008.03.002 18486483

[96] Lehnardt S Lehmann S Kaul D Tschimmel K Hoffmann O Cho S. Toll-like receptor 2 mediates CNS injury in focal cerebral ischemia. J Neuroimmunol (2007) 190(1-2):28–33. doi: 10.1016/j.jneuroim.2007.07.023 17854911

[97] Lehnardt S Massillon L Follett P Jensen FE Ratan R Rosenberg PA. Activation of innate immunity in the CNS triggers neurodegeneration through a toll-like receptor 4-dependent pathway. Proc Natl Acad Sci U S A (2003) 100(14):8514–9. doi: 10.1073/pnas.1432609100 PMC166260 12824464

[98] Rosenberger K Derkow K Dembny P Krüger C Schott E Lehnardt S. The impact of single and pairwise toll-like receptor activation on neuroinflammation and neurodegeneration. J Neuroinflamm (2014) 20:11:166. doi: 10.1186/s12974-014-0166-7 PMC4182775 25239168

[99] Ngwa C Al Mamun A Qi S Sharmeen R Xu Y Liu F. Regulation of microglial activation in stroke in aged mice: a translational study. Aging (Albany NY) (2022) 14(15):6047–65. doi: 10.18632/aging.204216 PMC9417226 35963621

[100] Pradillo JM Fernández-López D García-Yébenes I Sobrado M Hurtado O Moro MA. Toll-like receptor 4 is involved in neuroprotection afforded by ischemic preconditioning. J Neurochem (2009) 109(1):287–94. doi: 10.1111/j.1471-4159.2009.05972.x 19200341

[101] Tang SC Arumugam TV Xu X Cheng A Mughal MR Jo DG. Pivotal role for neuronal toll-like receptors in ischemic brain injury and functional deficits. Proc Natl Acad Sci U S A (2007) 104(34):13798–803. doi: 10.1073/pnas.0702553104 PMC1959462 17693552

[102] Hu X Liou AK Leak RK Xu M An C Suenaga J. Neurobiology of microglial action in CNS injuries: receptor-mediated signaling mechanisms and functional roles. Prog Neurobiol (2014) 119-120:60–84. doi: 10.1016/j.pneurobio.2014.06.002 24923657 PMC4121732

[103] Trincavelli ML Melani A Guidi S Cuboni S Cipriani S Pedata F. Regulation of A(2A) adenosine receptor expression and functioning following permanent focal ischemia in rat brain. J Neurochem (2008) 104(2):479–90. doi: 10.1111/j.1471-4159.2007.04990.x 17953669

[104] Rivera-Oliver M Díaz-Ríos M. Using caffeine and other adenosine receptor antagonists and agonists as therapeutic tools against neurodegenerative diseases: a review. Life Sci (2014) 101(1-2):1–9. doi: 10.1016/j.lfs.2014.01.083 24530739 PMC4115368

[105] Abbracchio MP Burnstock G Verkhratsky A Zimmermann H. Purinergic signalling in the nervous system: an overview. Trends Neurosci (2009) 32(1):19–29. doi: 10.1016/j.tins.2008.10.001 19008000

[106] Burnstock G. Purinergic signalling and disorders of the central nervous system. Nat Rev Drug Discovery (2008) 7(7):575–90. doi: 10.1038/nrd2605 18591979

[107] Burnstock G. Physiology and pathophysiology of purinergic neurotransmission. Physiol Rev (2007) 87(2):659–797. doi: 10.1152/physrev.00043.2006 17429044

[108] Cavaliere F Florenzano F Amadio S Fusco FR Viscomi MT D'Ambrosi N. Up-regulation of P2X2, P2X4 receptor and ischemic cell death: prevention by P2 antagonists. Neuroscience (2003) 120(1):85–98. doi: 10.1016/s0306-4522(03)00228-8 12849743

[109] Gendron FP Chalimoniuk M Strosznajder J Shen S González FA Weisman GA. P2X7 nucleotide receptor activation enhances IFN gamma-induced type II nitric oxide synthase activity in BV-2 microglial cells. J Neurochem (2003) 87(2):344–52. doi: 10.1046/j.1471-4159.2003.01995.x 14511112

[110] Shiratori M Tozaki-Saitoh H Yoshitake M Tsuda M Inoue K. P2X7 receptor activation induces CXCL2 production in microglia through NFAT and PKC/MAPK pathways. J Neurochem (2010) 114(3):810–9. doi: 10.1111/j.1471-4159.2010.06809.x 20477948

[111] Ajami B Bennett JL Krieger C McNagny KM Rossi FM. Infiltrating monocytes trigger EAE progression, but do not contribute to the resident microglia pool. Nat Neurosci (2011) 14(9):1142–9. doi: 10.1038/nn.2887 21804537

[112] Savarin-Vuaillat C Ransohoff RM. Chemokines and chemokine receptors in neurological disease: raise, retain, or reduce? Neurotherapeutics (2007) 4(4):590–601. doi: 10.1016/j.nurt.2007.07.004 17920540 PMC7479679

[113] Inose Y Kato Y Kitagawa K Uchiyama S Shibata N. Activated microglia in ischemic stroke penumbra upregulate MCP-1 and CCR2 expression in response to lysophosphatidylcholine derived from adjacent neurons and astrocytes. Neuropathology (2015) 35(3):209–23. doi: 10.1111/neup.12182 25443158

[114] Dimitrijevic OB Stamatovic SM Keep RF Andjelkovic AV. Absence of the chemokine receptor CCR2 protects against cerebral ischemia/reperfusion injury in mice. Stroke (2007) 38(4):1345–53. doi: 10.1161/01.STR.0000259709.16654.8f 17332467

[115] Schilling M Strecker JK Ringelstein EB Schäbitz WR Kiefer R. The role of CC chemokine receptor 2 on microglia activation and blood-borne cell recruitment after transient focal cerebral ischemia in mice. Brain Res (2009) 1289:79–84. doi: 10.1016/j.brainres.2009.06.054 19559679

[116] Lue LF Walker DG Brachova L Beach TG Rogers J Schmidt AM. Involvement of microglial receptor for advanced glycation endproducts (RAGE) in alzheimer's disease: identification of a cellular activation mechanism. Exp Neurol (2001) 171(1):29–45. doi: 10.1006/exnr.2001.7732 11520119

[117] Zhai DX Kong QF Xu WS Bai SS Peng HS Zhao K. RAGE expression is up-regulated in human cerebral ischemia and pMCAO rats. Neurosci Lett (2008) 445(1):117–21. doi: 10.1016/j.neulet.2008.08.077 18782604

[118] Menini T Ikeda H Kimura S Gugliucci A. Circulating soluble RAGE increase after a cerebrovascular event. Clin Chem Lab Med (2014) 52(1):109–16. doi: 10.1515/cclm-2012-0813 23492566

[119] Muhammad S Barakat W Stoyanov S Murikinati S Yang H Tracey KJ. The HMGB1 receptor RAGE mediates ischemic brain damage. J Neurosci (2008) 28(46):12023–31. doi: 10.1523/JNEUROSCI.2435-08.2008 PMC4597312 19005067

[120] Hu X Li P Guo Y Wang H Leak RK Chen S. Microglia/macrophage polarization dynamics revealel mechanism of injury expansion after focal cerebral ischemia. Stroke (2012) 43(11):3063–70. doi: 10.1161/STROKEAHA.112.659656 22933588

[121] Norden DM Godbout JP. Review: microglia of the aged brain: primed to be activated and resistant to regulation. Neuropathol Appl Neurobiol (2013) 39(1):19–34. doi: 10.1111/j.1365-2990.2012.01306.x 23039106 PMC3553257

[122] O'Neill LA Hardie DG. Metabolism of inflammation limited by AMPK and pseudo-starvation. Nature (2013) ;493(7432):346–55. doi: 10.1038/nature11862 23325217

[123] Salvany S Casanovas A Piedrafita L Tarabal O Hernández S Calderó J. Microglial recruitment and mechanisms involved in the disruption of afferent synaptic terminals on spinal cord motor neurons after acute peripheral nerve injury. Glia (2021) 69(5):1216–40. doi: 10.1002/glia.23959 PMC7986680 33386754

[124] Perego C Fumagalli S De Simoni MG. Temporal pattern of expression and colocalization of microglia/macrophage phenotypekers following brain ischemic injury in mice. J Neuroinflamm (2011) 8:174. doi: 10.1186/1742-2094-8-174 PMC3251548 22152337

[125] Denes A Vidyasagar R Feng J Narvainen J McColl BW Kauppinen RA. Proliferating resident microglia after focal cerebral ischaemia in mice. J Cereb Blood Flow Metab (2007) 27(12):1941–53. doi: 10.1038/sj.jcbfm.9600495 17440490

[126] Vázquez-Villoldo N Domercq M Martín A Llop J Gómez-Vallejo V Matute C. P2X4 receptors control the fate and survival of activated microglia. Glia (2014) 62(2):171–84. doi: 10.1002/glia.22596 24254916

[127] Barnabei L Laplantine E Mbongo W Rieux-Laucat F Weil R. NF-κB: At the borders of autoimmunity and inflammation. Front Immunol (2021) 12:716469. doi: 10.3389/fimmu.2021.716469 34434197 PMC8381650

[128] Cheng QJ Ohta S Sheu KM Spreafico R Adelaja A Taylor B. NF-κB dynamics determine the stimulus specificity of epigenomic reprogramming in macrophages. Science (2021) 372(6548):1349–53. doi: 10.1126/science.abc0269.5 PMC8489855 34140389

[129] Lenglet S Montecucco F Mach F. Role of matrix metalloproteinases in animal models of ischemic stroke. Curr Vasc Pharmacol (2015) 13(2):161–6. doi: 10.2174/15701611113116660161 24188490

[130] Kim SR Jung YR An HJ Kim DH Jang EJ Choi YJ. Anti-wrinkle and anti-inflammatory effects of active garlic components and the inhibition of MMPs *via* NF-κB signaling. PloS One (2013) 8(9):e73877. doi: 10.1371/journal.pone.0073877 24066081 PMC3774756

[131] Shin HM Minter LM Cho OH Gottipati S Fauq AH Golde TE. Notch1ments NF-kappaB activity by facilitating its nuclear retention. EMBO J (2006) 25(1):129–38. doi: 10.1038/sj.emboj.7600902 PMC1356346 16319921

[132] Jha NK Chen WC Kumar S Dubey R Tsai LW Kar R. Molecular mechanisms of developmental pathways in neurological disorders: a pharmacological and therapeutic review. Open Biol (2022) 12(3):210289. doi: 10.1098/rsob.210289 35291879 PMC8924757

[133] Wei Z Chigurupati S Arumugam TV Jo DG Li H Chan SL. Notch activation enhances the microglia-mediated inflammatory response associated with focal cerebral ischemia. Stroke (2011) 42(9):2589–94. doi: 10.1161/STROKEAHA.111.614834 21737799

[134] Qin H Holdbrooks AT Liu Y Reynolds SL Yanagisawa LL Benveniste EN. SOCS3 deficiency promotes M1 macrophage polarization and inflammation. J Immunol (2012) 189(7):3439–48. doi: 10.4049/jimmunol.1201168 PMC4184888 22925925

[135] Takeda H Yamaguchi T Yano H Tanaka J. Microglial metabolic disturbances and neuroinflammation in cerebral infarction. J Pharmacol Sci (2021) 145(1):130–9. doi: 10.1016/j.jphs.2020.11.007 33357771

[136] Lai CS Lee JH Ho CT Liu CB Wang JM Wang YJ. Rosmanol potently inhibits lipopolysaccharide-induced iNOS and COX-2 expression through downregulating MAPK, NF-kappaB, STAT3 and C/EBP signaling pathways. J Agric Food Chem (2009) 57(22):10990–8. doi: 10.1021/jf9025713 19856917

[137] Jover-Mengual T Miyawaki T Latuszek A Alborch E Zukin RS Etgen AM. Acute estradiol protects CA1 neurons from ischemia-induced apoptotic cell death *via* the PI3K/Akt pathway. Brain Res (2010) 1321:1–12. doi: 10.1016/j.brainres.2010.01.046 20114038 PMC2836484

[138] Hu X Chen J Wang L Ivashkiv LB. Crosstalk among jak-STAT, toll-like receptor, and ITAM-dependent pathways in macrophage activation. J Leukoc Biol (2007) 82(2):237–43. doi: 10.1189/jlb.1206763 17502339

[139] Barić A Dobrivojević Radmilović M. Microglia and bradykinin cross talk in poststroke cognitive impairment in diabetes. Am J Physiol Cell Physiol (2021) 320(4):C613–8. doi: 10.1152/ajpcell.00402.2020 33502951

[140] Hosoi T Oba T Ozawa K. ER stress-mediated regulation of immune function under glucose-deprived condition in glial cells: up- and down-regulation of PGE2 + IFNγ-induced IL-6 and iNOS expressions. BiochemBiophys Res Commun (2013) 441(2):525–8. doi: 10.1016/j.bbrc.2013.10.109 24177007

[141] Hosoi T Honda M Oba T Ozawa K. ER stress upregulated PGE_2_/IFNγ-induced IL-6 expression and down-regulated iNOS expression in glial cells. Sci Rep (2013) 3:3388. doi: 10.1038/srep03388 24291824 PMC3844943

[142] Li D Wang C Yao Y Chen L Liu G Zhang R. mTORC1 pathway disruption ameliorates brain inflammation following stroke *via* a shift in microglia phenotype from M1 type to M2 type. FASEB J (2016) 30(10):3388–99. doi: 10.1096/fj.201600495R 27342766

[143] Lukiw WJ Pogue AI. Vesicular transport of encapsulated microRNA between glial and neuronal cells. Int J Mol Sci (2020) 21(14):5078. doi: 10.3390/ijms21145078 32708414 PMC7404393

[144] Vasudeva K Munshi A. miRNA dysregulation in ischaemic stroke: Focus on diagnosis, prognosis, therapeutic and protective biomarkers. Eur J Neurosci (2020) 52(6):3610–27. doi: 10.1111/ejn.14695 32022336

[145] Wang SW Liu Z Shi ZS. Non-coding RNA in acute ischemic stroke: Mechanisms, biomarkers and therapeutic targets. Cell Transplant (2018) 27(12):1763–77. doi: 10.1177/0963689718806818 PMC6300774 30362372

[146] Maniati E Bossard M Cook N Candido JB Emami-Shahri N Nedospasov SA. Crosstalk between the canonical NF-κB and notch signaling pathways inhibits pparγ expression and promotes pancreatic cancer progression in mice. J Clin Invest (2011) 121(12):4685–99. doi: 10.1172/JCI45797 PMC3225987 22056382

[147] Zhao Y Patzer A Herdegen T Gohlke P Culman J. Activation of cerebral peroxisome proliferator-activated receptors gamma promotes neuroprotection by attenuation of neuronal cyclooxygenase-2 overexpression after focal cerebral ischemia in rats. FASEB J (2006) 20(8):1162–75. doi: 10.1096/fj.05-5007com 16770015

[148] Ruffell D Mourkioti F Gambardella A Kirstetter P Lopez RG Rosenthal N. A CREB-C/EBPbeta cascade induces M2 macrophage-specific gene expression and promotes muscle injury repair. Proc Natl Acad Sci U.S.A. (2009) 106(41):17475–80. doi: 10.1073/pnas.0908641106 PMC2762675 19805133

[149] Gorgoni B Maritano D Marthyn P Righi M Poli V. C/EBP beta gene inactivation causes both impaired and enhanced gene expression and inverse regulation of IL-12 p40 and p35 mRNAs in macrophages. J Immunol (2002) 168(8):4055–62. doi: 10.4049/jimmunol.168.8.4055 11937564

[150] Stein B Cogswell PC Baldwin AS Jr. Functional and physical associations between NF-kappa b and C/EBP family members: a rel domain-bZIP interaction. Mol Cell Biol (1993) 13(7):3964–74. doi: 10.1128/mcb.13.7.3964-3974.1993 PMC359940 8321203

[151] Chistiakov DA Myasoedova VA Revin VV Orekhov AN Bobryshev YV. The impact of interferon-regulatory factors to macrophage differentiation and polarization into M1 and M2. Immunobiology (2018) 223(1):101–11. doi: 10.1016/j.imbio.2017.10.005 29032836

[152] Qin BY Liu C Srinath H Lam SS Correia JJ Derynck R. Crystal structure of IRF-3 in complex with CBP. Structure (2005) 13(9):1269–77. doi: 10.1016/j.str.2005.06.011 16154084

[153] Zhao SC Ma LS Chu ZH Xu H Wu WQ Liu F. Regulation of microglial activation in stroke. Acta Pharmacol Sin (2017) 38(4):445–58. doi: 10.1038/aps.2016.162 PMC5386316 28260801

[154] Neher JJ Emmrich JV Fricker M Mander PK Théry C Brown GC. Phagocytosis executes delayed neuronal death after focal brain ischemia. Proc Natl Acad Sci U S A (2013) 110(43):E4098–107. doi: 10.1073/pnas.1308679110 PMC3808587 24101459

[155] Zhao X Wang H Sun G Zhang J Edwards NJ Aronowski J. Neuronal interleukin-4 as a modulator of microglial pathways and ischemic brain damage. J Neurosci (2015) 35(32):11281–91. doi: 10.1523/JNEUROSCI.1685-15.2015 PMC4532758 26269636

[156] Noda M Doi Y Liang J Kawanokuchi J Sonobe Y Takeuchi H. Fractalkine attenuates excito-neurotoxicity *via* microglial clearance of damaged neurons and antioxidant enzyme heme oxygenase-1 expression. J Biol Chem (2016) 291(27):14388. doi: 10.1074/jbc.A110.169839 27371564 PMC4933193

[157] Dénes A Ferenczi S Halász J Környei Z Kovács KJ. Role of CX3CR1 (fractalkine receptor) in brain damage and inflammation induced by focal cerebral ischemia in mouse. J Cereb Blood Flow Metab (2008) 28(10):1707–21. doi: 10.1038/jcbfm.2008.64 18575457

[158] Franco EC Cardoso MM Gouvêia A Pereira A Gomes-Leal W. Modulation of microglial activation enhances neuroprotection and functional recovery derived from bonerow mononuclear cell transplantation after cortical ischemia. Neurosci Res (2012) 73(2):122–32. doi: 10.1016/j.neures.2012.03.006 22465414

[159] Wu LJ Wu G Akhavan Sharif MR Baker A Jia Y Fahey FH. The voltage-gated proton channel Hv1 enhances brain damage from ischemic stroke. Nat Neurosci (2012) 15(4):565–73. doi: 10.1038/nn.3059 PMC3314139 22388960

[160] Brown GC Neher JJ. Microglial phagocytosis of live neurons. Nat Rev Neurosci (2014) 15(4):209–16. doi: 10.1038/nrn3710 24646669

[161] Nimmerjahn A Kirchhoff F Helmchen F. Resting microglial cells are highly dynamic surveillants of brain parenchyma *in vivo* . Science (2005) 308(5726):1314–8. doi: 10.1126/science.1110647 15831717

[162] Parajuli B Koizumi S. Strategies for manipulating microglia to determine their role in the healthy and diseased brain. Neurochem Res (2022) 9:1–11. doi: 10.1007/s11064-022-03742-6 PMC9462627 36085395

[163] Ransohoff RM Brown MA. Innate immunity in the central nervous system. J Clin Invest (2012) 122(4):1164–71. doi: 10.1172/JCI58644 PMC3314450 22466658

[164] Young AP Denovan-Wright EM. The dynamic role of microglia and the endocannabinoid system in neuroinflammation. Front Pharmacol (2022) 12:806417. doi: 10.3389/fphar.2021.806417 35185547 PMC8854262

[165] Araki T Ikegaya Y Koyama R. The effects of microglia- and astrocyte-derived factors on neurogenesis in health and disease. Eur J Neurosci (2021) 54(5):5880–901. doi: 10.1111/ejn.14969 PMC8451940 32920880

[166] Pascual O Ben Achour S Rostaing P Triller A Bessis A. Microglia activation triggers astrocyte-mediated modulation of excitatory neurotransmission. Proc Natl Acad Sci U S A (2012) 109(4):E197–205. doi: 10.1073/pnas.1111098109 PMC3268269 22167804

[167] Rouach N Avignone E Même W Koulakoff A Venance L Blomstrand F. Gapctions and connexin expression in the normal and pathological central nervous system. Biol Cell (2002) 94(7-8):457–75. doi: 10.1016/s0248-4900(02)00016-3 12566220

[168] ouach N Calvo CF Glowinski J Giaume C. Brain macrophages inhibit gapctional communication and downregulate connexin 43 expression in cultured astrocytes. Eur J Neurosci (2002) 15(2):403–7. doi: 10.1046/j.0953-816x.2001.01868.x 11849308

[169] Zhang D Ren J Luo Y He Q Zhao R Chang J. T Cell response in ischemic stroke: From mechanisms to translational insights. Front Immunol (2021) 12:707972. doi: 10.3389/fimmu.2021.707972 34335623 PMC8320432

[170] Selvaraj UM Stowe AM. Long-term T cell responses in the brain after an ischemic stroke. Discovery Med (2017) 24(134):323–33. PMC5893311 29373810

[171] Gill D Veltkamp R. Dynamics of T cell responses after stroke. CurrOpinPharmacol (2016) 26:26–32. doi: 10.1016/j.coph.2015.09.009 26452204

[172] Wu Y Li J Shou J Zhang W Chen C. Diverse functions and mechanisms of regulatory T cell in ischemic stroke. Exp Neurol (2021) 343:113782. doi: 10.1016/j.expneurol.2021.113782 34116055

[173] Chen S Wu H Klebe D Hong Y Zhang J Tang J. Regulatory T cell in stroke: a new paradigm for immune regulation. Clin Dev Immunol (2013) 2013:689827. doi: 10.1155/2013/689827 23983771 PMC3747621

[174] Arya AK Hu B. Brain-gut axis after stroke. Brain Circ (2018) 4(4):165–73. doi: 10.4103/bc.bc_32_18 PMC6329216 30693343

[175] Meng H. Neuronal soluble FasL mediates M1-type microglia after ischemic stroke[3 (plasma cell polarization and mechanism[D]. Nanjing: Nanjing University (2016).

[176] Li Q. Tregs attenuates inflammatory injury in cerebral hemorrhage through IL-10/STAT3-induced M2-type polarization of microglia [D]. Chongqing: Chongqing Medical University (2018).

[177] Wang S Zhang H Xu Y. Crosstalk between microglia and T cells contributes to brain damage and recovery after ischemic stroke. Neurol Res (2016) 38(6):495–503. doi: 10.1080/01616412.2016.1188473 27244271

[178] Coughlan T Gibson C Murphy S. Modulatory effects of progesterone on inducible nitric oxide synthase expression *in vivo* and *in vitro* . J Neurochem (2005) 93(4):932–42. doi: 10.1111/j.1471-4159.2005.03068.x 15857396

[179] Park EM Cho S Frys KA Glickstein SB Zhou P Anrather J. Inducible nitric oxide synthase contributes to gender differences in ischemic brain injury. J Cereb Blood Flow Metab (2006) 26(3):392–401. doi: 10.1038/sj.jcbfm.9600194 16049426

[180] Liesz A Zhou W Na SY Hämmerling GJ Garbi N Karcher S. Boosting regulatory T cells limits neuroinflammation in permanent cortical stroke. J Neurosci (2013) 33(44):17350–62. doi: 10.1523/JNEUROSCI.4901-12.2013 PMC6618366 24174668

[181] Kleinschnitz C Kraft P Dreykluft A Hagedorn I Göbel K Schuhmann MK. Regulatory T cells are strong promoters of acute ischemic stroke in mice by inducing dysfunction of the cerebral microvasculature. Blood (2013) 121(4):679–91. doi: 10.1182/blood-2012-04-426734 PMC3790947 23160472

[182] Sakaguchi S Ono M Setoguchi R Yagi H Hori S Fehervari Z. Foxp3+ CD25+ CD4+ natural regulatory T cells in dominant self-tolerance and autoimmune disease. Immunol Rev (2006) 212:8–27. doi: 10.1111/j.0105-2896.2006.00427.x 16903903

[183] Ren X Akiyoshi K Vandenbark AA Hurn PD Offner H. CD4+FoxP3+ regulatory T-cells in cerebral ischemic stroke. Metab Brain Dis (2011) 26(1):87–90. doi: 10.1007/s11011-010-9226-6 21082336 PMC3070853

[184] Zouggari Y Ait-Oufella H Waeckel L Vilar J Loinard C Cochain C. Regulatory T cells modulate postischemic neovascularization. Circulation (2009) 6;120(14):1415–25. doi: 10.1161/CIRCULATIONAHA.109.875583 19770391

[185] Michels M Sonai B Dal-Pizzol F. Polarization of microglia and its role in bacterialsis. J Neuroimmunol (2017) 303:90–8. doi: 10.1016/j.jneuroim.2016.12.015 28087076

[186] Savage ND de Boer T Walburg KV Joosten SA van Meijgaarden K Geluk A. Human anti-inflammatory macrophages induce Foxp3+ GITR+ CD25+ regulatory T cells, which suppress *via* membrane-bound TGFbeta-1. J Immunol (2008) 181(3):2220–6. doi: 10.4049/jimmunol.181.3.2220 18641362

[187] Passaro AP Lebos AL Yao Y Stice SL. Immune response in neurological pathology: Emerging role of central and peripheral immune crosstalk. Front Immunol (2021) 12:676621. doi: 10.3389/fimmu.2021.676621 34177918 PMC8222736

[188] Shi L Sun Z Su W Xu F Xie D Zhang Q. Treg cell-derived osteopontin promotes microglia-mediated white matter repair after ischemic stroke. Immunity (2021) 54(7):1527–1542.e8. doi: 10.1016/j.immuni.2021.04.022 34015256 PMC8282725

[189] Rashid ChehrehBargh S Tafakhori A Masoumi F Rahmani F Ahmadi M Namdar A. Evaluation of regulatory T lymphocytes and IL2Ra and FOXP3 gene expression in peripheral mononuclear cells from patients with amyotrophic lateral sclerosis. Ir J Med Sci (2018) 187(4):1065–71. doi: 10.1007/s11845-018-1793-2 29574662

[190] Li Q Zhang W Tan Y Zhao G Zhang X Zhang P. Tregs attenuate the inflammatory injury of cerebral hemorrhage through IL-10/STAT3-induced M2-type polarization of microglia. J Third Military Med Univ (2018) 40(16):1461–8. doi: 10.16016/j.1000-5404.201802129

[191] Zhou K. Treg cells reduce inflammatory injury in cerebral hemorrhage by regulating microglia/macrophage polarization through IL-10/GSK3β/PTEN signaling. Third Military Med Univ (2016).

[192] Liu Y Gao J Peng M Meng H Ma H Cai P. A review on central nervous system effects of gastrodin. Front Pharmacol (2018) 9:24. doi: 10.3389/fphar.2018.00024 29456504 PMC5801292

[193] Deng C Chen H Meng Z Meng S. Gastrodin and vascular dementia: Advances and current perspectives. Evid Based Complement Alternat Med (2022) 2022:2563934. doi: 10.1155/2022/2563934 35463081 PMC9019412

[194] Ma D. Salvia polyphenolic acid attenuates experimental cerebral ischemia-reperfusion injury through microglia P2X7/NLRP3/GSDMD pathway [D]. Liaoning Univ Traditional Chin Med (2021). doi: 10.27213/d.cnki.glnzc.2021.000034

[195] Yang W Chen X Pan J Ge H Yin K Wu Z. Malibatol a protects against brain injury through reversing mitochondrial dysfunction in experimental stroke. Neurochem Int (2015) 80:33–40. doi: 10.1016/j.neuint.2014.11.003 25447763

[196] Pan J Jin JL Ge HM Yin KL Chen X Han LJ. Malibatol a regulates microglia M1/M2 polarization in experimental stroke in a PPARγ-dependent manner. J Neuroinflamm (2015) 14:12:51. doi: 10.1186/s12974-015-0270-3 PMC4378556 25889216

[197] Weng L Wu Z Zheng W Meng H Han L Wang S. Malibatol a enhances alternative activation of microglia by inhibiting phosphorylation of mammalian Ste20-like kinase1 in OGD-BV-2 cells. Neurol Res (2016) 38(4):342–8. doi: 10.1080/01616412.2016.1174423 27098434

[198] Meng T Xiao D Muhammed A Deng J Chen L He J. Anti-inflammatory action and mechanisms of resveratrol. Molecules (2021) 26(1):229. doi: 10.3390/molecules26010229 33466247 PMC7796143

[199] Pignet AL Schellnegger M Hecker A Kohlhauser M Kotzbeck P Kamolz LP. Resveratrol-induced signal transduction in wound healing. Int J Mol Sci (2021) 22(23):12614. doi: 10.3390/ijms222312614 34884419 PMC8657598

[200] Zhou DD Luo M Huang SY Saimaiti A Shang A Gan RY. Effects and mechanisms of resveratrol on aging and age-related diseases. Oxid Med Cell Longev (2021) 2021:9932218. doi: 10.1155/2021/9932218 34336123 PMC8289612

[201] Parsamanesh N Asghari A Sardari S Tasbandi A Jamialahmadi T Xu S. Resveratrol and endothelial function: A literature review. Pharmacol Res (2021) 170:105725. doi: 10.1016/j.phrs.2021.105725 34119624

[202] Wang Q Xu J Rottinghaus GE Simonyi A Lubahn D Sun GY. Resveratrol protects against global cerebral ischemic injury in gerbils. Brain Res (2002) 958(2):439–47. doi: 10.1016/s0006-8993(02)03543-6 12470882

[203] Shin JA Lee H Lim YK Koh Y Choi JH Park EM. Therapeutic effects of resveratrol during acute periods following experimental ischemic stroke. J Neuroimmunol (2010) 227(1-2):93–100. doi: 10.1016/j.jneuroim.2010.06.017 20655115

[204] Bischoff-Kont I Fürst R. Benefits of ginger and its constituent 6-shogaol in inhibiting inflammatory processes. Pharm (Basel) (2021) 14(6):571. doi: 10.3390/ph14060571 PMC8232759 34203813

[205] Ooi SL Campbell R Pak SC Golombick T Manoharan A Ramakrishna R. Is 6-shogaol an effective phytochemical for patients with lower-risk myelodysplastic syndrome? a narrative review. Integr Cancer Ther (2021) 20:15347354211065038. doi: 10.1177/15347354211065038 34930049 PMC8728773

[206] Kou X Wang X Ji R Liu L Qiao Y Lou Z. Occurrence, biological activity and metabolism of 6-shogaol. Food Funct (2018) 9(3):1310–27. doi: 10.1039/c7fo01354j 29417118

[207] Ha SK Moon E Ju MS Kim DH Ryu JH Oh MS. 6-shogaol, a ginger product, modulates neuroinflammation: a new approach to neuroprotection. Neuropharmacology (2012) 63(2):211–23. doi: 10.1016/j.neuropharm.2012.03.016 22465818

[208] Gaire BP Kwon OW Park SH Chun KH Kim SY Shin DY. Neuroprotective effect of 6-paradol in focal cerebral ischemia involves the attenuation of neuroinflammatory responses in activated microglia. PloS One (2015) 10(3):e0120203. doi: 10.1371/journal.pone.0120203 25789481 PMC4366308

[209] Sarrica A Kirika N Romeo M Salmona M Diomede L. Safety and toxicology of magnolol and honokiol. Planta Med (2018) 84(16):1151–64. doi: 10.1055/a-0642-1966 29925102

[210] Rauf A Olatunde A Imran M Alhumaydhi FA Aljohani ASM Khan SA. Honokiol: A review of its pharmacological potential and therapeutic insights. Phytomedicine (2021) 90:153647. doi: 10.1016/j.phymed.2021.153647 34362632

[211] Rauf A Patel S Imran M Maalik A Arshad MU Saeed F. Honokiol: An anticancer lignan. BioMed Pharmacother (2018) 107:555–62. doi: 10.1016/j.biopha.2018.08.054 30114639

[212] Zhang P Liu X Zhu Y Chen S Zhou D Wang Y. Honokiol inhibits the inflammatory reaction during cerebral ischemia reperfusion by suppressing NF-κB activation and cytokine production of glial cells. Neurosci Lett (2013) 8:534:123–7. doi: 10.1016/j.neulet.2012.11.052 23262090

[213] Hwang IK Yoo KY Li H Park OK Lee CH Choi JH. Indole-3-propionic acid attenuates neuronal damage and oxidative stress in the ischemic hippocampus. J Neurosci Res (2009) 87(9):2126–37. doi: 10.1002/jnr.22030 19235887

[214] Zhang L Li DC Liu LF. Paeonol: pharmacological effects and mechanisms of action. Int Immunopharmacol (2019) 72:413–21. doi: 10.1016/j.intimp.2019.04.033 31030097

[215] Wang J Wu G Chu H Wu Z Sun J. Paeonol derivatives and pharmacological activities: A review of recent progress. Mini Rev Med Chem (2020) 20(6):466–82. doi: 10.2174/1389557519666191015204223 31644406

[216] Wu M Yu Z Li X Zhang X Wang S Yang S. Paeonol for the treatment of atherosclerotic cardiovascular disease: A pharmacological and mechanistic overview. Front Cardiovasc Med (2021) 8:690116. doi: 10.3389/fcvm.2021.690116 34368250 PMC8333700

[217] Vellasamy S Murugan D Abas R Alias A Seng WY Woon CK. Biological activities of paeonol in cardiovascular diseases: A review. Molecules (2021) 26(16):4976. doi: 10.3390/molecules26164976 34443563 PMC8400614

[218] Hsieh CL Cheng CY Tsai TH Lin IH Liu CH Chiang SY. Paeonol reduced cerebral infarction involving the superoxide anion and microglia activation in ischemia-reperfusion injured rats. J Ethnopharmacol (2006) 106(2):208–15. doi: 10.1016/j.jep.2005.12.027 16458462

[219] Chu C Deng J Man Y Qu Y. Green tea extracts epigallocatechin-3-gallate for different treatments. BioMed Res Int (2017) 2017:5615647. doi: 10.1155/2017/5615647 28884125 PMC5572593

[220] Cione E La Torre C Cannataro R Caroleo MC Plastina P Gallelli L. Quercetin, epigallocatechin gallate, curcumin, and resveratrol: From dietary sources to human MicroRNA modulation. Molecules (2019) 25(1):63. doi: 10.3390/molecules25010063 31878082 PMC6983040

[221] Singh BN Shankar S Srivastava RK. Green tea catechin, epigallocatechin-3-gallate (EGCG): mechanisms, perspectives and clinical applications. Biochem Pharmacol (2011) 82(12):1807–21. doi: 10.1016/j.bcp.2011.07.093 PMC4082721 21827739

[222] Pervin M Unno K Takagaki A Isemura M Nakamura Y. Function of green tea catechins in the brain: Epigallocatechin gallate and its metabolites. Int J Mol Sci (2019) 20(15):3630. doi: 10.3390/ijms20153630 31349535 PMC6696481

[223] Sutherland BA Shaw OM Clarkson AN Jackson DN Sammut IA Appleton I. Neuroprotective effects of (-)-epigallocatechin gallate following hypoxia-ischemia-induced brain damage:el mechanisms of action. FASEB J (2005) 19(2):258–60. doi: 10.1096/fj.04-2806fje 15569775

[224] Shan Z Nisar MF Li M Zhang C Wan CC. Theaflavin chemistry and its health benefits. Oxid Med Cell Longev (2021) 2021:6256618. doi: 10.1155/2021/6256618 34804369 PMC8601833

[225] Cai Q Ji S Li M Zheng S Zhou X Guo H. Theaflavin-regulated imd condensates control drosophila intestinal homeostasis and aging. iScience (2021) 24(3):102150. doi: 10.1016/j.isci.2021.102150 33665569 PMC7905455

[226] Khan N Mukhtar H. Tea polyphenols in promotion of human health. Nutrients (2018) 11(1):39. doi: 10.3390/nu11010039 30585192 PMC6356332

[227] Cai F Li CR Wu JL Chen JG Liu C Min Q. Theaflavin ameliorates cerebral ischemia-reperfusion injury in rats through its anti-inflammatory effect and modulation of STAT-1. Mediators Inflamm (2006) 2006(5):30490. doi: 10.1155/MI/2006/30490 17392572 PMC1657077

[228] Zhou R Yang Z Tang X Tan Y Wu X Liu F. Propofol protects against focal cerebral ischemia *via* inhibition of microglia-mediated proinflammatory cytokines in a rat model of experimental stroke. PloS One (2013) 8(12):e82729. doi: 10.1371/journal.pone.0082729 24349350 PMC3857282

[229] Jung YS Park JH Kim H Kim SY Hwang JY Hong KW. Probucol inhibits LPS-induced microglia activation and ameliorates brain ischemic injury in normal and hyperlipidemic mice. Acta Pharmacol Sin (2016) 37(8):1031–44. doi: 10.1038/aps.2016.51 PMC4973385 27345627

[230] Xiong XY Liu L Yang QW. Refocusing neuroprotection in cerebral reperfusion era: New challenges and strategies. Front Neurol (2018) 9:249. doi: 10.3389/fneur.2018.00249 29740385 PMC5926527

[231] Jickling GC Liu D Stamova B Ander BP Zhan X Lu A. Hemorrhagic transformation after ischemic stroke in animals and humans. J Cereb Blood Flow Metab (2014) 34(2):185–99. doi: 10.1038/jcbfm.2013.203 PMC3915212 24281743

[232] Wang ZF Wang J Zhang HY Tang XC. Huperzine a exhibits anti-inflammatory and neuroprotective effects in a rat model of transient focal cerebral ischemia. J Neurochem (2008) 106(4):1594–603. doi: 10.1111/j.1471-4159.2008.05504.x 18513368

[233] Cai Y He Q Hu F Guo Q Li Y. The effect of emodin on the activation of microglia and the expression of inflammatory factors after cerebral ischemia. Shizhen Traditional Chin Med (2021) 32(03):574–7. doi: 10.3969/j.issn.1008-0805.2021.03.17.

[234] Zhang Y Cai Y Hu F Guo Q Li Y He Q. Effects of chrysophanol on the activation of microglia and the expression of inflammatory factors in rats with cerebral ischemia injury. Chin Pharm (2020) 31(23):2858–63. doi: 10.6039/j.issn.1001-0408.2020.23.08

[235] Guo Q He Q Hu F Cai Y Li Y. Chrysophanol regulates inflammatory response of microglia through TLR4/NF-kB signaling pathway. Med Inf (2020) 33(19):51–54+58. doi: 10.3969/j.issn.1006-1959.2020.19.016

[236] Zhang J Wu C Gao L Du G Qin X. Astragaloside IV derived from astragalus membranaceus: A research review on the pharmacological effects. Adv Pharmacol (2020) 87:89–112. doi: 10.1016/bs.apha.2019.08.002 32089240

[237] Xia ML Xie XH Ding JH Du RH Hu G. Astragaloside IV inhibits astrocyte senescence: implication in parkinson's disease. J Neuroinflamm (2020) 17(1):105. doi: 10.1186/s12974-020-01791-8 PMC7137443 32252767

[238] Zheng X Gan H Li L Hu X Fang Y Chu L. Astragaloside IV inhibits inflammatory response after cerebral ischemia in rats by promoting the M2-type polarization of microglia/macrophages. Zhejiang J Univ (Medical Edition) (2020) 49(06):679–86. doi: 10.3785/j.issn.1008-9292.2020.12.02 PMC10412416 33448170

[239] He Y Shi H Liu H Wu H Zhang B Wu X. Astragaloside IV regulates STAT1/IκB/NF-κB signaling pathway and inhibits γ-interferon-induced BV-2 cell activation. China J Traditional Chin Med (2015) 40(01):124–8. doi: 10.4268/cjcmm20150124 25993801

[240] Yu Y Zhou L Yang Y Liu Y. Cycloastragenol: An exciting novel candidate for age-associated diseases. Exp Ther Med (2018) 16(3):2175–82. doi: 10.3892/etm.2018.6501 PMC6122403 30186456

[241] Shen CY Jiang JG Yang L Wang DW Zhu W. Anti-ageing active ingredients from herbs and nutraceuticals used in traditional Chinese medicine: pharmacological mechanisms and implications for drug discovery. Br J Pharmacol (2017) 174(11):1395–425. doi: 10.1111/bph.13631 PMC5429334 27659301

[242] Li M Han B Zhao H Xu C Xu D Sieniawska E. Biological active ingredients of astragali radix and its mechanisms in treating cardiovascular and cerebrovascular diseases. Phytomedicine (2022) 98:153918. doi: 10.1016/j.phymed.2021.153918 35104756

[243] Li M Li SC Dou BK Zou YX Han HZ Liu DX. Cycloastragenol upregulates SIRT1 expression, attenuates apoptosis and suppresses neuroinflammation after brain ischemia. Acta Pharmacol Sin (2020) 41(8):1025–32. doi: 10.1038/s41401-020-0386-6 PMC7471431 32203080

[244] Barker EC Kim BG Yoon JH Tochtrop GP Letterio JJ Choi SH. Potent suppression of both spontaneous and carcinogen-induced colitis-associated colorectal cancer in mice by dietary celastrol supplementation. Carcinogenesis (2018) 39(1):36–46. doi: 10.1093/carcin/bgx115 29069290 PMC5862246

[245] Ren B Liu H Gao H Liu S Zhang Z Fribley AM. Celastrol induces apoptosis in hepatocellular carcinoma cells *via* targeting ER-stress/UPR. Oncotarget (2017) 8(54):93039–50. doi: 10.18632/oncotarget.21750 PMC5696242 29190976

[246] Astry B Venkatesha SH Laurence A Christensen-Quick A Garzino-Demo A Frieman MB. Celastrol, a Chinese herbal compound, controls autoimmune inflammation by altering the balance of pathogenic and regulatory T cells in the target organ. Clin Immunol (2015) 157(2):228–38. doi: 10.1016/j.clim.2015.01.011 PMC4410084 25660987

[247] Luo D Guo Y Cheng Y Zhao J Wang Y Rong J. Natural product celastrol suppressed macrophage M1 polarization against inflammation in diet-induced obese mice *via* regulating Nrf2/HO-1, MAP kinase and NF-κB pathways. Aging (Albany NY) (2017) 9(10):2069–82. doi: 10.18632/aging.101302 PMC5680556 29040966

[248] Bai S Hu Z Yang Y Yin Y Li W Wu L. Anti-inflammatory and neuroprotective effects of triptolide *via* the NF-κB signaling pathway in a rat MCAO model. Anat Rec (Hoboken) (2016) 299(2):256–66. doi: 10.1002/ar.23293 26575184

[249] Jiang M Liu X Zhang D Wang Y Hu X Xu F. Celastrol treatment protects against acute ischemic stroke-induced brain injury by promoting an IL-33/ST2 axis-mediated microglia/macrophage M2 polarization. J Neuroinflamm (2018) 15(1):78. doi: 10.1186/s12974-018-1124-6 PMC5853059 29540209

[250] Lalloo DG Shingadia D Bell DJ Beeching NJ Whitty CJM Chiodini PL. PHE advisory committee on malaria prevention in UK travellers. UK malaria treatment guidelines 2016. J Infect (2016) 72(6):635–49. doi: 10.1016/j.jinf.2016.02.001 PMC7132403 26880088

[251] Zuo S Ge H Li Q. Artesunate protected blood-brain barrier *via* sphingosine 1 phosphate receptor 1/phosphatidylinositol 3 kinase pathway after subarachnoid hemorrhage in rats. Mol Neurobiol (2017) 54:1213–28. doi: 10.1007/s12035-016-9732-6 26820677

[252] Clemmer L Martins YC Zanini GM Frangos JA Carvalho LJ. Artemether and artesunate show the highest efficacies in rescuing mice with late-stage cerebral malaria and rapidlyrease leukocyte accumulation in the brain. Antimicrob Agents Chemother (2011) 55(4):1383–90. doi: 10.1128/AAC.01277-10 PMC3067152 21220531

[253] Ho WE Peh HY Chan TK Wong WS. Artemisinins: pharmacological actions beyond anti-malarial. Pharmacol Ther (2014) 142(1):126–39. doi: 10.1016/j.pharmthera.2013.12.001 24316259

[254] Zuo S Li Q Liu X Feng H Chen Y. The potential therapeutic effects of artesunate on stroke and other central nervous system diseases. BioMed Res Int (2016) 2016:1489050. doi: 10.1155/2016/1489050 28116289 PMC5223005

[255] Lai L Chen Y Tian X Li X Zhang X Lei J. Artesunate alleviates hepatic fibrosis induced by multiple pathogenic factors and inflammation through the inhibition of LPS/TLR4/NF-κB signaling pathway in rats. Eur J Pharmacol (2015) 15;765:234–41. doi: 10.1016/j.ejphar.2015.08.040 26318197

[256] Okorji UP Olajide OA. A semi-synthetic derivative of artemisinin, artesunate inhibits prostaglandin E2 production in LPS/IFNγ-activated BV2 microglia. Bioorg Med Chem (2014) 22:4726–34. doi: 10.1016/j.bmc.2014.07.007 25074847

[257] Lu H Wang B Cui N Zhang Y. Artesunate suppresses oxidative and inflammatory processes by activating Nrf2 and ROS dependent p38 MAPK and protects against cerebral ischemia reperfusion injury. Mol Med Rep (2018) 17:6639–46. doi: 10.3892/mmr.2018.8666 29512760

[258] Liu Y Dang W Zhang S Wang L Zhang X. Artesunate attenuates inflammatory injury and inhibits the NF-κB pathway in a mouse model of cerebral ischemia. J Int Med Res (2021) 49(11):3000605211053549. doi: 10.1177/03000605211053549 34743632 PMC8579345

[259] Bailly C Vergoten G. Glycyrrhizin: An alternative drug for the treatment of COVID-19 infection and the associated respiratory syndrome? Pharmacol Ther (2020) 214:107618. doi: 10.1016/j.pharmthera.2020.107618 32592716 PMC7311916

[260] Al-Kamel H Grundmann O. Glycyrrhizin as a potential treatment for the novel coronavirus (COVID-19). Mini Rev Med Chem (2021) 21(16):2204–8. doi: 10.2174/1389557521666210210160237 33568033

[261] Kim SW Lim CM Lee HK Lee JK. The use of stronger neo-minophagen c, a glycyrrhizin-containing preparation, in robust neuroprotection in the postischemic brain. Anat Cell Biol (2011) 44(4):304–13. doi: 10.5115/acb.2011.44.4.304 PMC3254884 22254159

[262] Gaid M Biedermann E Füller J Haas P Behrends S Krull R. Biotechnological production of hyperforin for pharmaceutical formulation. Eur J Pharm Biopharm (2018) 126:10–26. doi: 10.1016/j.ejpb.2017.03.024 28377273

[263] Billard C Merhi F Bauvois B. Mechanistic insights into the antileukemic activity of hyperforin. Curr Cancer Drug Targets (2013) 13(1):1–10. doi: 10.2174/156800913804486601 22924417

[264] Chrubasik-Hausmann S Vlachojannis J McLachlan AJ. Understanding drug interactions with St john's wort (Hypericum perforatum l.): impact of hyperforin content. J Pharm Pharmacol (2019) 71(1):129–38. doi: 10.1111/jphp.12858 29411879

[265] Ma L Pan X Zhou F Liu K Wang L. Hyperforin protects against acute cerebral ischemic injury through inhibition of interleukin-17A-mediated microglial activation. Brain Res (2018) 1678:254–61. doi: 10.1016/j.brainres.2017.08.023 28870826

[266] Xu AL Zheng GY Wang ZJ Chen XD Jiang Q. Neuroprotective effects of ilexonin a following transient focal cerebral ischemia in rats. Mol Med Rep (2016) 13(4):2957–66. doi: 10.3892/mmr.2016.4921 PMC4805093 26936330

[267] Xu AL Zheng GY Ye HY Chen XD Jiang Q. Characterization of astrocytes and microglial cells in the hippocampal CA1 region after transient focal cerebral ischemia in rats treated with ilexonin a. Neural Regener Res (2020) 15(1):78–85. doi: 10.4103/1673-5374.264465 PMC6862412 31535655

[268] Zhang BQ Zheng GY Han Y Chen XD Jiang Q. Ilexonin a promotes neuronal proliferation and regeneration *via* activation of the canonical wnt signaling pathway after cerebral ischemia reperfusion in rats. Evid Based Complement Alternat Med (2016) 2016:9753189. doi: 10.1155/2016/9753189 27057202 PMC4739464

[269] Asgharzade S Khorrami MB Forouzanfar F. Neuroprotective effect of herniarin following transient focal cerebral ischemia in rats. Metab Brain Dis (2021) 36(8):2505–10. doi: 10.1007/s11011-021-00841-1 34519909

[270] Friedli MJ Inestrosa NC. Huperzine a and its neuroprotective molecular signaling in alzheimer's disease. Molecules (2021) 26(21):6531. doi: 10.3390/molecules26216531 34770940 PMC8587556

[271] Shukla M Wongchitrat P Govitrapong P. A synopsis of multitarget potential therapeutic effects of huperzine a in diverse pathologies-emphasis on alzheimer's disease pathogenesis. Neurochem Res (2022) 47(5):1166–82. doi: 10.1007/s11064-022-03530-2 35122609

[272] Li X Li W Tian P Tan T. Delineating biosynthesis of huperzine a, a plant-derived medicine for the treatment of alzheimer's disease. Biotechnol Adv (2022) 60:108026. doi: 10.1016/j.biotechadv.2022.108026 35914626

[273] Wang J Zhang HY Tang XC. Huperzine a improves chronic inflammation and cognitiveline in rats with cerebral hypoperfusion. J Neurosci Res (2010) 88(4):807–15. doi: 10.1002/jnr.22237 19795377

[274] Baska A Leis K Gałązka P. Berberine in the treatment of diabetes mellitus: A review. Endocr Metab Immune Disord Drug Targets (2021) 21(8):1379–86. doi: 10.2174/1568026620666201022144405 33092516

[275] Xu X Yi H Wu J Kuang T Zhang J Li Q. Therapeutic effect of berberine on metabolic diseases: Both pharmacological data and clinical evidence. BioMed Pharmacother (2021) 133:110984. doi: 10.1016/j.biopha.2020.110984 33186794

[276] Kim M Shin MS Lee JM Cho HS Kim CJ Kim YJ. Inhibitory effects of isoquinoline alkaloid berberine on ischemia-induced apoptosis *via* activation of phosphoinositide 3-Kinase/Protein kinase b signaling pathway. Int Neurourol J (2014) 18(3):115–25. doi: 10.5213/inj.2014.18.3.115 PMC4180161 25279238

[277] Jiang W Fan W Gao T Li T Yin Z Guo H. Analgesic mechanism of sinomenine against chronic pain. Pain Res Manage (2020) 2020:1876862. doi: 10.1155/2020/1876862 PMC7225909 32454918

[278] Zhang MW Wang XH Shi J Yu JG. Sinomenine in cardio-cerebrovascular diseases: Potential therapeutic effects and pharmacological evidences. Front Cardiovasc Med (2021) 8:749113. doi: 10.3389/fcvm.2021.749113 34660748 PMC8517137

[279] Tang J Raza A Chen J Xu H. A systematic review on the sinomenine derivatives. Mini Rev Med Chem (2018) 18(11):906–17. doi: 10.2174/1389557517666171123212557 29173167

[280] Qiu J Wang M Zhang J Cai Q Lu D Li Y. The neuroprotection of sinomenine against ischemic stroke in mice by suppressing NLRP3 inflammasome *via* AMPK signaling. Int Immunopharmacol (2016) 40:492–500. doi: 10.1016/j.intimp.2016.09.024 27769021

[281] He C Wang Z Shi J. Pharmacological effects of icariin. Adv Pharmacol (2020) 87:179–203. doi: 10.1016/bs.apha.2019.10.004 32089233

[282] Wang S Ma J Zeng Y Zhou G Wang Y Zhou W. Icariin, an up-and-Coming bioactive compound against neurological diseases: Network pharmacology-based study and literature review. Drug Des Devel Ther (2021) 15:3619–41. doi: 10.2147/DDDT.S310686 PMC8384151 34447243

[283] Wang Z Wang D Yang D Zhen W Zhang J Peng S. The effect of icariin on bone metabolism and its potential clinical application. Osteoporos Int (2018) 29(3):535–44. doi: 10.1007/s00198-017-4255-1 29110063

[284] Wang M Gao H Li W Wu B. Icariin and its metabolites regulate lipid metabolism: From effects to molecular mechanisms. BioMed Pharmacother (2020) 131:110675. doi: 10.1016/j.biopha.2020.110675 32861069

[285] Tang B Zhang Y Wu Y Liu Y Liu M Hu W. The effect of icariin on neuroprotection and microglial TLR4/NF-κB pathway in cerebral ischemia-reperfusion rats ]. Chin J Exp Prescriptions (2020) 26(22):47–52. doi: 10.13422/j.cnki.syfjx.20201865

[286] Park SJ Choi H Kim JH Kim CS. Antifibrotic effects of eupatilin on TGF-β1-treated human vocal fold fibroblasts. PloS One (2021) 16(3):e0249041. doi: 10.1371/journal.pone.0249041 33765087 PMC7993872

[287] Wu Z Zou B Zhang X Peng X. Eupatilin regulates proliferation and cell cycle of cervical cancer by regulating hedgehog signalling pathway. Cell Biochem Funct (2020) 38(4):428–35. doi: 10.1002/cbf.3493 31926121

[288] Cinar AK Ozal SA Serttas R Erdogan S. Eupatilin attenuates TGF-β2-induced proliferation and epithelial-mesenchymal transition of retinal pigment epithelial cells. Cutan Ocul Toxicol (2021) 40(2):103–14. doi: 10.1080/15569527.2021.1902343 33719768

[289] Sapkota A Gaire BP Cho KS Jeon SJ Kwon OW Jang DS. Eupatilin exerts neuroprotective effects in mice with transient focal cerebral ischemia by reducing microglial activation. PloS One (2017) 12(2):e0171479. doi: 10.1371/journal.pone.0171479 28178289 PMC5298292

[290] Okuyama S Morita M Miyoshi K Nishigawa Y Kaji M Sawamoto A. Heptamethoxyflavone, a citrus flavonoid, on protection against memory impairment and neuronal cell death in a global cerebral ischemia mouse model. Neurochem Int (2014) 70:30–8. doi: 10.1016/j.neuint.2014.03.008 24657445

[291] Khan S Kamal MA. Can wogonin be used in controlling diabetic cardiomyopathy? Curr Pharm Des (2019) 25(19):2171–7. doi: 10.2174/1381612825666190708173108 31298148

[292] Sharifi-Rad J Herrera-Bravo J Salazar LA Shaheen S Abdulmajid Ayatollahi S Kobarfard F. The therapeutic potential of wogonin observed in preclinical studies. Evid Based Complement Alternat Med (2021) 2021:9935451. doi: 10.1155/2021/9935451 34221094 PMC8221866

[293] Huynh DL Ngau TH Nguyen NH Tran GB Nguyen CT. Potential therapeutic and pharmacological effects of wogonin: an updated review. Mol Biol Rep (2020) 47(12):9779–89. doi: 10.1007/s11033-020-05972-9 33165817

[294] Lee H Kim YO Kim H Kim SY Noh HS Kang SS. Flavonoid wogonin from medicinal herb is neuroprotective by inhibiting inflammatory activation of microglia. FASEB J (2003) 17(13):1943–4. doi: 10.1096/fj.03-0057fje 12897065

[295] Zhou YX Zhang H Peng C. Puerarin: a review of pharmacological effects. Phytother Res (2014) 28(7):961–75. doi: 10.1002/ptr.5083 24339367

[296] Zhang L. Pharmacokinetics and drug delivery systems for puerarin, a bioactive flavone from traditional Chinese medicine. Drug Deliv (2019) 26(1):860–9. doi: 10.1080/10717544.2019.1660732 PMC6758605 31524010

[297] Kulczyński B Gramza-Michałowska A Suliburska J Sidor A. Puerarin-an isoflavone with beneficial effects on bone health. Front Biosci (Landmark Ed) (2021) 26(12):1653–67. doi: 10.52586/5058 34994179

[298] Zhou YX Zhang H Peng C. Effects of puerarin on the prevention and treatment of cardiovascular diseases. Front Pharmacol (2021) 12:771793. doi: 10.3389/fphar.2021.771793 34950032 PMC8689134

[299] Lim DW Lee C Kim IH Kim YT. Anti-inflammatory effects of total isoflavones from pueraria lobata on cerebral ischemia in rats. Molecules (2013) 18(9):10404–12. doi: 10.3390/molecules180910404 PMC6270189 23989686

[300] Derosa G Maffioli P D'Angelo A Di Pierro F. A role for quercetin in coronavirus disease 2019 (COVID-19). Phytother Res (2021) 35(3):1230–6. doi: 10.1002/ptr.6887 PMC7675685 33034398

[301] Singh P Arif Y Bajguz A Hayat S. The role of quercetin in plants. Plant Physiol Biochem (2021) 166:10–9. doi: 10.1016/j.plaphy.2021.05.023 34087741

[302] Hosseini A Razavi BM Banach M Hosseinzadeh H. Quercetin and metabolic syndrome: A review. Phytother Res (2021) 35(10):5352–64. doi: 10.1002/ptr.7144 34101925

[303] Shen P Lin W Deng X Ba X Han L Chen Z. Potential implications of quercetin in autoimmune diseases. Front Immunol (2021) 12:689044. doi: 10.3389/fimmu.2021.689044 34248976 PMC8260830

[304] Pierro F DI Khan A Bertuccioli A Maffioli P Derosa G Khan S. Quercetin phytosome® as a potential candidate for managing COVID-19. Minerva Gastroenterol (Torino) (2021) 67(2):190–5. doi: 10.23736/S2724-5985.20.02771-3 33016666

[305] Imran M Saeed F Gilani SA Shariati MA Imran A Afzaal M. Fisetin: An anticancer perspective. Food Sci Nutr (2020) 9(1):3–16. doi: 10.1002/fsn3.1872 33473265 PMC7802565

[306] Kirkland JL Tchkonia T. Senolytic drugs: from discovery to translation. J Intern Med (2020) 288(5):518–36. doi: 10.1111/joim.13141 PMC7405395 32686219

[307] Farooqi AA Naureen H Zahid R Youssef L Attar R Xu B. Cancer chemopreventive role of fisetin: Regulation of cell signaling pathways in different cancers. Pharmacol Res (2021) 172:105784. doi: 10.1016/j.phrs.2021.105784 34302980

[308] Kubina R Krzykawski K Kabała-Dzik A Wojtyczka RD Chodurek E Dziedzic A. Fisetin, a potent anticancer flavonol exhibiting cytotoxic activity against neoplastic malignant cells and cancerous conditions: A scoping, comprehensive review. Nutrients (2022) 14(13):2604. doi: 10.3390/nu14132604 35807785 PMC9268460

[309] Gelderblom M Leypoldt F Lewerenz J Birkenmayer G Orozco D Ludewig P. Theflavonoid fisetin attenuates postischemic immune cell infiltration, activation andinfarct size after transient cerebral middle artery occlusion in mice. J Cereb.Blood Flow Metab (2012) 32(5):835–43. doi: 10.1038/jcbfm.2011.189 PMC3345911 22234339

[310] Wen L He T Yu A Sun S Li X Wei J. Breviscapine: A review on its phytochemistry, pharmacokinetics and therapeutic effects. Am J Chin Med (2021) 49(6):1369–97. doi: 10.1142/S0192415X21500646 34263720

[311] Ma Y Li H Guan S. Enhancement of the oral bioavailability of breviscapine by nanoemulsions drug delivery system. Drug Dev Ind Pharm (2015) 41(2):177–82. doi: 10.3109/03639045.2014.947510 25113432

[312] Wu L Liu M Fang Z. Combined therapy of hypertensive nephropathy with breviscapine injection and antihypertensive drugs: A systematic review and a meta-analysis. Evid Based Complement Alternat Med (2018) 2018:2958717. doi: 10.1155/2018/2958717 30671127 PMC6317107

[313] Long Y Yang Q Xiang Y Zhang Y Wan J Liu S. Nose to brain drug delivery - a promising strategy for active components from herbal medicine for treating cerebral ischemia reperfusion. Pharmacol Res (2020) 159:104795. doi: 10.1016/j.phrs.2020.104795 32278035

[314] Yuan Y Zha H Rangarajan P Ling EA Wu C. Anti-inflammatory effects of edaravone and scutellarin in activated microglia in experimentally induced ischemia injury in rats and in BV-2 microglia. BMC Neurosci (2014) 22:15:125. doi: 10.1186/s12868-014-0125-3 PMC4247200 25416145

[315] Yuan Y Rangarajan P Kan EM Wu Y Wu C Ling EA. Scutellarin regulates the notch pathway and affects the migration and morphological transformation of activated microglia in experimentally induced cerebral ischemia in rats and in activated BV-2 microglia. J Neuroinflamm (2015) 20:12:11. doi: 10.1186/s12974-014-0226-z PMC4316603 25600517

[316] Naz S Imran M Rauf A Orhan IE Shariati MA Iahtisham-Ul-Haq. Chrysin: Pharmacological and therapeutic properties. Life Sci (2019) 235:116797. doi: 10.1016/j.lfs.2019.116797 31472146

[317] Mani R Natesan V. Chrysin: Sources, beneficial pharmacological activities, and molecular mechanism of action. Phytochemistry (2018) 145:187–96. doi: 10.1016/j.phytochem.2017.09.016 29161583

[318] Stompor-Gorący M Bajek-Bil A Machaczka M. Chrysin: Perspectives on contemporary status and future possibilities as pro-health agent. Nutrients (2021) 13(6):2038. doi: 10.3390/nu13062038 34198618 PMC8232110

[319] Yao Y Chen L Xiao J Wang C Jiang W Zhang R. Chrysin protectsagainst focal cerebral ischemia/reperfusion injury in mice through attenuation ofoxidative stress and inflammation. Int J Mol Sci (2014) 15(11):20913–26. doi: 10.3390/ijms151120913 PMC4264203 25402649

[320] Daussin FN Heyman E Burelle Y. Effects of (-)-epicatechin on mitochondria. Nutr Rev (2021) 79(1):25–41. doi: 10.1093/nutrit/nuaa094 32989466

[321] Cremonini E Iglesias DE Kang J Lombardo GE Mostofinejad Z Wang Z. (-)-Epicatechin and the comorbidities of obesity. Arch Biochem Biophys (2020) 690:108505. doi: 10.1016/j.abb.2020.108505 32679195

[322] Si H Lai CQ Liu D. Dietary epicatechin, a novel anti-aging bioactive small molecule. Curr Med Chem (2021) 28(1):3–18. doi: 10.2174/0929867327666191230104958 31886745

[323] Qu Z Liu A Li P Liu C Xiao W Huang J. Advances in physiological functions and mechanisms of (-)-epicatechin. Crit Rev Food Sci Nutr (2021) 61(2):211–33. doi: 10.1080/10408398.2020.1723057 32090598

[324] Leonardo CC Agrawal M Singh N Moore JR Biswal S Dore S. Oraladministration of the flavanol (-)-epicatechin bolsters endogenous protectionagainst focal ischemia through the Nrf2 cytoprotective pathway. Eur J Neurosci (2013) 38(11):3659–68. doi: 10.1111/ejn.12362 PMC6010187 24112193

[325] Zhang X Xie L Long J Xie Q Zheng Y Liu K. Salidroside: A review of its recent advances in synthetic pathways and pharmacological properties. Chem Biol Interact (2021) 339:109268. doi: 10.1016/j.cbi.2020.109268 33617801

[326] Han J Xiao Q Lin YH Zheng ZZ He ZD Hu J. Neuroprotective effects of salidroside on focal cerebral ischemia/reperfusion injury involve the nuclear erythroid 2-related factor 2 pathway. Neural Regener Res (2015) 10(12):1989–96. doi: 10.4103/1673-5374.172317 PMC4730824 26889188

[327] Liu X Wen S Yan F Liu K Liu L Wang L. Salidroside provides neuroprotection by modulating microglial polarization after cerebral ischemia. J Neuroinflamm (2018) 15(1):39. doi: 10.1186/s12974-018-1081-0 PMC5807735 29426336

[328] Pan LL Yang Y Hui M Wang S Li CY Zhang H. Sulfation predominates the pharmacokinetics, metabolism, and excretion of forsythin in humans: major enzymes and transporters identified. Acta Pharmacol Sin (2021) 42(2):311–22. doi: 10.1038/s41401-020-0481-8 PMC8026975 32860005

[329] Han Z Guo J Meng F Liao H Deng Y Huang Y. Genetic toxicology and safety pharmacological evaluation of forsythin. Evid Based Complement Alternat Med (2021) 2021:6610793. doi: 10.1155/2021/6610793 34239584 PMC8233079

[330] Yuan WJ Zhang SP He ZY He YX He SQ Liu LJ. Comparative transcriptome analyses identify genes involved into the biosynthesis of forsythin and forsythoside a in forsythia suspensa. Funct Integr Genomics (2022) 22(5):731–41. doi: 10.1007/s10142-022-00887-z 35870023

[331] Kim JM Kim S Kim DH Lee CH Park SJ Jung JW. Neuroprotective effect of forsythiaside against transient cerebral global ischemia in gerbil. Eur J Pharmacol (2011) 660(2-3):326–33. doi: 10.1016/j.ejphar.2011.03.051 21501605

[332] Yin X Hu H Shen X Li X Pei J Xu J. Ginseng omics for ginsenoside biosynthesis. Curr Pharm Biotechnol (2021) 22(5):570–8. doi: 10.2174/1389201021666200807113723 32767915

[333] Nakhjavani M Smith E Townsend AR Price TJ Hardingham JE. Anti-angiogenic properties of ginsenoside Rg3. Molecules (2020) 25(21):4905. doi: 10.3390/molecules25214905 33113992 PMC7660320

[334] Lee HW Lee MS Kim TH Alraek T Zaslawski C Kim JW. Ginseng for erectile dysfunction. Cochrane Database Syst Rev (2021) 4(4):CD012654. doi: 10.1002/14651858.CD012654.pub2 33871063 PMC8094213

[335] Guo M Shao S Wang D Zhao D Wang M. Recent progress in polysaccharides from panax ginseng c. a. Meyer. Food Funct (2021) 12(2):494–518. doi: 10.1039/d0fo01896a 33331377

[336] Ye R Yang Q Kong X Han J Zhang X Zhang Y. Ginsenoside Rd attenuates early oxidative damage and sequential inflammatory response after transient focal ischemia in rats. Neurochem Int (2011) 58(3):391–8. doi: 10.1016/j.neuint.2010.12.015 21185898

[337] Zhu J Jiang Y Wu L Lu T Xu G Liu X. Suppression of local inflammation contributes to the neuroprotective effect of ginsenoside Rb1 in rats with cerebral ischemia. Neuroscience (2012) 202:342–51. doi: 10.1016/j.neuroscience.2011.11.070 22173011

[338] Yuan Y Zhai Y Chen J Xu X Wang H. Kaempferol ameliorates oxygen-glucose Deprivation/Reoxygenation-induced neuronal ferroptosis by activating Nrf2/SLC7A11/GPX4 axis. Biomolecules (2021) 11(7):923. doi: 10.3390/biom11070923 34206421 PMC8301948

[339] Imran M Salehi B Sharifi-Rad J Aslam Gondal T Saeed F Imran A. Kaempferol: A key emphasis to its anticancer potential. Molecules (2019) 24(12):2277. doi: 10.3390/molecules24122277 31248102 PMC6631472

[340] Dabeek WM Marra MV. Dietary quercetin and kaempferol: Bioavailability and potential cardiovascular-related bioactivity in humans. Nutrients (2019) 11(10):2288. doi: 10.3390/nu11102288 31557798 PMC6835347

[341] Calderón-Montaño JM Burgos-Morón E Pérez-Guerrero C López-Lázaro M. A review on the dietary flavonoid kaempferol. Mini Rev Med Chem (2011) 11(4):298–344. doi: 10.2174/138955711795305335 21428901

[342] Devi KP Malar DS Nabavi SF Sureda A Xiao J Nabavi SM. Kaempferol and inflammation: From chemistry to medicine. Pharmacol Res (2015) 99:1–10. doi: 10.1016/j.phrs.2015.05.002 25982933

[343] Yu L Chen C Wang LF Kuang X Liu K Zhang H. Neuroprotective effect of kaempferol glycosides against brain injury and neuroinflammation by inhibiting the activation of NF-kappaB and STAT3 in transient focal stroke. PloS One (2013) 8(2):e55839. doi: 10.1371/journal.pone.0055839 23437066 PMC3577792

[344] Zhang L Wei W. Anti-inflammatory and immunoregulatory effects of paeoniflorin and total glucosides of paeony. Pharmacol Ther (2020) 207:107452. doi: 10.1016/j.pharmthera.2019.107452 31836457

[345] Ji Y Dou YN Zhao QW Zhang JZ Yang Y Wang T. Paeoniflorin suppresses TGF-β mediated epithelial-mesenchymal transition in pulmonary fibrosis through a smad-dependent pathway. Acta Pharmacol Sin (2016) 37(6):794–804. doi: 10.1038/aps.2016.36 27133302 PMC4954768

[346] Wang XL Feng ST Wang YT Chen NH Wang ZZ Zhang Y. Paeoniflorin: A neuroprotective monoterpenoid glycoside with promising anti-depressive properties. Phytomedicine (2021) 90:153669. doi: 10.1016/j.phymed.2021.153669 34334273

[347] Luo XQ Li A Yang X Xiao X Hu R Wang TW. Paeoniflorin exerts neuroprotective effects by modulating the M1/M2 subset polarization of microglia/macrophages in the hippocampal CA1 region of vascular dementia rats *via* cannabinoid receptor 2. Chin Med (2018) 13:14. doi: 10.1186/s13020-018-0173-1 29560022 PMC5859430

[348] Cohen JA Chun J. Mechanisms of fingolimod's eficacy and adverse effects in multiple sclerosis. Ann Neurol (2011) 69(5):759–77. doi: 10.1186/s12974-018-1323-1 21520239

[349] Gaire BP Song MR Choi JW. Sphingosine 1-phosphate receptor subtype 3(S1P3)contributes to brain injury after transient focal cerebral ischemia *via* modulating microglial activation and their M1 polarization. J Neuroinflamm (2018) 15(1):284. doi: 10.1186/s12974-018-1323-1 PMC6180378 30305119

[350] Li X Wang MH Qin C. Fingolimod suppresses neuronal autophagy through the mTOR/p70S6K pathway and alleviates ischemic brain damage in mice. PloS One (2017) 12(11):e0188748. doi: 10.1371/journal.pone.0188748 29186197 PMC5706683

[351] Kraft P Gob E Gobel K. FTY720 ameliorates acute ischemic stroke in mice by reducing thrombo-inflammation but not by direct neuroprotection. Stroke (2013) 44(11):3202–10. doi: 10.1161/STROKEAHA.113.002880 24029635

[352] Campos F Qin T Castillo J. Fingolimod reduces hemorrhagic transformation associated with delayed tissue plasminogen activator treatment in a mouse thromboembolic model. Stroke (2013) 44(2):505–11. doi: 10.1161/STROKEAHA.112.679043 PMC3586809 23287783

[353] Ali AI Jing GY. Areview of recent advances in neuroprotective potential of 3-n-butylphthalide and its derivatives. BioMed Res Int (2016) 2016:1–9. doi: 10.1155/2016/5012341 PMC5178327 28053983

[354] Zhao H Yun W Zhang Q. Mobilization of circulating endothelial progenitor cells by DL-3-N-butylphthalide in acute ischemic stroke patients. J StrokeCerebrovasc Dis (2016) 25(4):.752–760. doi: 10.1016/j.jstrokecerebrovasdis.2015.11.018 26775268

[355] Hu J Wen Q Wu Y Li B Gao P. The effect of butylphthalide on the brain edema, blood-brain barrier of rats after focal cerebral infarction and the expression of Rho A. Cell Biochem Biophys (2014) 69(2):363–8. doi: 10.1007/s12013-013-9808-0 24442989

[356] Li F Ma Q Zhao H Wang R Tao Z Fan Z. L-3-n-Butylphthalide reduces ischemic stroke injury and increases M2 microglial polarization. Metab Brain Dis (2018) 33(6):1995–2003. doi: 10.1007/s11011-018-0307-2 30117100 PMC6244772

[357] Han D. Danshensu borneol improves pressure-induced heart failure through the mTOR / β-TrcP / Nrf2 pathway [ d ]. Zhengzhou Univ (2021). doi: 10.27466/d.cnki.gzzdu.2021.000719

[358] Zhang Z. Brain targeting effect of danshensu borneol ester and its effect on p-glycoprotein on blood-brain barrier [ d ]. Hefei Univ Technol (2017).

[359] Liao S Wu J Liu R Wang S Luo J Yang Y. Ael compound DBZ ameliorates neuroinflammation in LPS-stimulated microglia and ischemic stroke rats: Role of Akt(Ser473)/GSK3β(Ser9)-mediated Nrf2 activation. Redox Biol (2020) 36:101644. doi: 10.1016/j.redox.2020.101644 32863210 PMC7371982

[360] Shabgah AG Suksatan W Achmad MH Bokov DO Abdelbasset WK Ezzatifar F. Arctigenin, an anti-tumor agent; a cutting-edge topic and up-to-the-minute approach in cancer treatment. Eur J Pharmacol (2021) 909:174419. doi: 10.1016/j.ejphar.2021.174419 34391770

[361] He Y Fan Q Cai T Huang W Xie X Wen Y. Molecular mechanisms of the action of arctigenin in cancer. BioMed Pharmacother (2018) 108:403–7. doi: 10.1016/j.biopha.2018.08.158 30236849

[362] Gao Q Yang M Zuo Z. Overview of the anti-inflammatory effects, pharmacokinetic properties and clinical efficacies of arctigenin and arctiin from arctium lappa l. Acta Pharmacol Sin (2018) 39(5):787–801. doi: 10.1038/aps.2018.32 29698388 PMC5943914

[363] Fan T Jiang WL Zhu J Feng Zhang Y. Arctigenin protects focal cerebral ischemia-reperfusion rats through inhibiting neuroinflammation. Biol Pharm Bull (2012) 35(11):2004–9. doi: 10.1248/bpb.b12-00463 22972486

[364] Mottaghi S Abbaszadeh H. A comprehensive mechanistic insight into the dietary and estrogenic lignans, arctigenin and sesamin as potential anticarcinogenic and anticancer agents. current status, challenges, and future perspectives. Crit Rev Food Sci Nutr (2022) 62(26):7301–18. doi: 10.1080/10408398.2021.1913568 33905270

[365] Anju VT Busi S Ranganathan S Ampasala DR Kumar S Suchiang K. Sesamin and sesamolin rescues caenorhabditis elegans from pseudomonas aeruginosa infection through the attenuation of quorum sensing regulated virulence factors. Microb Pathog (2021) 155:104912. doi: 10.1016/j.micpath.2021.104912 33932548

[366] Ahmad S Elsherbiny NM Haque R Khan MB Ishrat T Shah ZA. Sesamin attenuates neurotoxicity in mouse model of ischemic brain stroke. Neurotoxicology (2014) 45:100–10. doi: 10.1016/j.neuro.2014.10.002 25316624

[367] Jiao L Zhang J Li Z Liu H Chen Y Xu S. Edaravone alleviates delayed neuronal death and long-dated cognitive dysfunction of hippocampus after transient focal ischemia in wistar rat brains. Neuroscience (2011) 19:182:177–83. doi: 10.1016/j.neuroscience.2011.01.017 21241778

[368] Gao HJ Liu PF Li PW Huang ZY Yu FB Lei T. Ligustrazine monomer against cerebral ischemia/reperfusion injury. Neural Regener Res (2015) 10(5):832–40. doi: 10.4103/1673-5374.156991 PMC4468780 26109963

[369] Shao H He X Zhang L Du S Yi X Cui X. Efficacy of ligustrazine injection as adjunctive therapy in treating acute cerebral infarction: A systematic review and meta-analysis. Front Pharmacol (2021) 12:761722. doi: 10.3389/fphar.2021.761722 34880757 PMC8646035

[370] Zheng Q Huang YY Zhu PC Tong Q Bao XY Zhang QH. Ligustrazine exerts cardioprotection in animal models of myocardial Ischemia/Reperfusion injury: Preclinical evidence and possible mechanisms. Front Pharmacol (2018) 9:729. doi: 10.3389/fphar.2018.00729 30090062 PMC6068386

[371] Ni X Ni X Liu S Guo X. Medium- and long-term efficacy of ligustrazine plus conventional medication on ischemic stroke: a systematic review and meta-analysis. J Tradit Chin Med (2013) 33(6):715–20. doi: 10.1016/s0254-6272(14)60002-9 24660601

[372] Lin J Wang Q Zhou S Xu S Yao K. Tetramethylpyrazine: A review on its mechanisms and functions. BioMed Pharmacother (2022) 150:113005. doi: 10.1016/j.biopha.2022.113005 35483189

[373] Kao TK Chang CY Ou YC Chen WY Kuan YH Pan HC. Tetramethylpyrazine reduces cellular inflammatory response following permanent focal cerebral ischemia in rats. Exp Neurol (2013) 247:188–201. doi: 10.1016/j.expneurol.2013.04.010 23644042

[374] Britch SC Babalonis S Walsh SL. Cannabidiol: pharmacology and therapeutic targets. Psychopharmacol (Berl) (2021) 238(1):9–28. doi: 10.1007/s00213-020-05712-8 PMC7796924 33221931

[375] von Wrede R Helmstaedter C Surges R. Cannabidiol in the treatment of epilepsy. Clin Drug Investig (2021) 41(3):211–20. doi: 10.1007/s40261-021-01003-y PMC7946683 33559102

[376] Gaston TE Martin RC Szaflarski JP. Cannabidiol (CBD) and cognition in epilepsy. Epilepsy Behav (2021) 124:108316. doi: 10.1016/j.yebeh.2021.108316 34563808

[377] Batalla A Bos J Postma A Bossong MG. The impact of cannabidiol on human brain function: A systematic review. Front Pharmacol (2021) 11:618184. doi: 10.3389/fphar.2020.618184 33551817 PMC7858248

[378] Mori MA Meyer E Soares LM Milani H Guimarães FS de Oliveira RMW. Cannabidiol reduces neuroinflammation and promotes neuroplasticity and functional recovery after brain ischemia. Prog Neuropsychopharmacol Biol Psychiatry (2017) 3:75:94–105. doi: 10.1016/j.pnpbp.2016.11.005 27889412

[379] Xie Q Zhang L Xie L Zheng Y Liu K Tang H. Z-ligustilide: A review of its pharmacokinetics and pharmacology. Phytother Res (2020) 34(8):1966–91. doi: 10.1002/ptr.6662 32135035

[380] Zhang Q Liu J Duan H Li R Peng W Wu C. Activation of Nrf2/HO-1 signaling: An important molecular mechanism of herbal medicine in the treatment of atherosclerosis *via* the protection of vascular endothelial cells from oxidative stress. J Adv Res (2021) 34:43–63. doi: 10.1016/j.jare.2021.06.023 35024180 PMC8655139

[381] Tasneem S Liu B Li B Choudhary MI Wang W. Molecular pharmacology of inflammation: Medicinal plants as anti-inflammatory agents. Pharmacol Res (2019) 139:126–40. doi: 10.1016/j.phrs.2018.11.001 30395947

[382] Prud'homme GJ Kurt M Wang Q. Pathobiology of the klotho antiaging protein and therapeutic considerations. Front Aging (2022) 3:931331. doi: 10.3389/fragi.2022.931331 35903083 PMC9314780

[383] Ghosh N Ghosh R Bhat ZA Mandal V Bachar SC Nima ND. Advances in herbal medicine for treatment of ischemic brain injury. Nat Prod Commun (2014) 9(7):1045–55. doi: 10.1177/1934578X1400900739 25230523

[384] Kuang X Wang LF Yu L Li YJ Wang YN He Q. Ligustilide ameliorates neuroinflammation and brain injury in focal cerebral ischemia/reperfusion rats: involvement of inhibition of TLR4/peroxiredoxin 6 signaling. Free Radic Biol Med (2014) 71:165–75. doi: 10.1016/j.freeradbiomed.2014.03.028 24681253

[385] Ma L Tang L Yi Q. Salvianolic acids: Potential source of natural drugs for the treatment of fibrosis disease and cancer. Front Pharmacol (2019) 10:97. doi: 10.3389/fphar.2019.00097 30842735 PMC6391314

[386] Du G Song J Du L Zhang L Qiang G Wang S. Chemical and pharmacological research on the polyphenol acids isolated from danshen: A review of salvianolic acids. Adv Pharmacol (2020) 87:1–41. doi: 10.1016/bs.apha.2019.12.004 32089230

[387] Han R Huang H Xia W Liu J Luo H Tang J. Perspectives for forkhead box transcription factors in diabetic cardiomyopathy: Their therapeutic potential and possible effects of salvianolic acids. Front Cardiovasc Med (2022) 9:951597. doi: 10.3389/fcvm.2022.951597 36035917 PMC9403618

[388] Xu H Wang E Chen F Xiao J Wang M. Neuroprotective phytochemicals in experimental ischemic stroke: Mechanisms and potential clinical applications. Oxid Med Cell Longev (2021) 2021:6687386. doi: 10.1155/2021/6687386 34007405 PMC8102108

[389] Liu H Lu X Hu Y Fan X. Chemical constituents of panax ginseng and panax notoginseng explain why they differ in therapeutic efficacy. Pharmacol Res (2020) 161:105263. doi: 10.1016/j.phrs.2020.105263 33127555

[390] Zhang X Zhang B Zhang C Sun G Sun X. Effect of panax notoginseng saponins and major anti-obesity components on weight loss. Front Pharmacol (2021) 11:601751. doi: 10.3389/fphar.2020.601751 33841133 PMC8027240

[391] Ji C Zhang Q Shi R Li J Wang X Wu Z. Determination of the authenticity and origin of panax notoginseng: A review. J AOAC Int (2022) 105(6):1708–18. doi: 10.1093/jaoacint/qsac081 35894651

[392] Wang R Wang M Zhou J Wu D Ye J Sun G. Saponins in Chinese herbal medicine exerts protection in myocardial ischemia-reperfusion injury: Possible mechanism and target analysis. Front Pharmacol (2021) 11:570867. doi: 10.3389/fphar.2020.570867 33597866 PMC7883640

[393] Li Y Guo Q Huang J Wang Z. Antidepressant active ingredients from Chinese traditional herb panax notoginseng: A pharmacological mechanism review. Front Pharmacol (2022) 13:922337. doi: 10.3389/fphar.2022.922337 35795547 PMC9252462

[394] Tan MM Chen MH Han F Wang JW Tu YX. Role of bioactive constituents of panax notoginseng in the modulation of tumorigenesis: A potential review for the treatment of cancer. Front Pharmacol (2021) 12:738914. doi: 10.3389/fphar.2021.738914 34776959 PMC8578715

[395] Jia Z Chen H Zhao L Yuan Q Yin M Wang S. Salvia polyphenolic acid combined with panax notoginseng saponins can regulate the polarization of M1/M2 type microglia on cerebral ischemia-regeneration in rats. influence of perfusion injury. Tianjin Traditional Chin Med (2020) 37(07):824–30. doi: 10.11656/j.issn.1672-1519.2020.07.26

[396] Zhang M Wang S Mao L. Omega-3 fatty acids protect the brain against ischemic injury by activating Nrf2 and upregulating heme oxygenase 1. JNeurosci (2014) 34(5):1903–15. doi: 10.1523/JNEUROSCI.4043-13.2014 PMC3905150 24478369

[397] Yang B Ren XL Huang H Guo XJ Ma AG Li D. Circulating long-chain n-3 polyunsaturated fatty acid and incidence of stroke:a meta-analysis of prospective cohort studies. Oncotarget (2017) 8(48):83781–91. doi: 10.18632/oncotarget.19530 PMC5663554 29137382

[398] Cai M Zhang W Weng Z Stetler RA Jiang X Shi Y. Promoting neurovascular recovery in aged mice after ischemic stroke -prophylactic effect of omega.3 polyunsaturated fatty acids. Aging Dis (2017) 8(5):531–45. 10.14336/AD.2017.0520 PMC5614319 28966799

[399] Berressem D Koch K Franke N Klein J Eckert GP. Intravenous treatment with a longchain omega-3 lipid emulsion provides neuroprotection in a murine model of ischemic stroke:a pilot study. PloS One (2016) 11(11):e0167329. 27902774 10.1371/journal.pone.0167329 PMC5130273

[400] Jiang X Pu H Hu X Wei Z Hong D Zhang W. A post-stroke therapeutic regimen with omega-3 polyunsaturated fatty acids that promotes white matter integrity and beneficial microglial responses after cerebral ischemia. Transl Stroke Res (2016) 7(6):548–61. doi: 10.1007/s12975-016-0502-6 PMC5125517 27714669

[401] Xie W Zhu T Dong X Nan F Meng X Zhou P. HMGB1-triggered triggered inflammation inhibition of notoginseng leaf triterpenes against cerebral ischemia and reperfusion injury *via* MAPK and NF-κB signaling pathways. Biomolecules (2019) 9(10):512. doi: 10.3390/biom9100512 31547018 PMC6843331

[402] Liu H Zhang Z Zang C Wang L Yang H Sheng C. GJ-4 ameliorates memory impairment in focal cerebral ischemia/reperfusion of rats *via* inhibiting JAK2/STAT1-mediated neuroinflammation. J Ethnopharmacol (2021) 267:113491. doi: 10.1016/j.jep.2020.113491 33091490

[403] Qin C Fan WH Liu Q. Fingolimod protects against ischemic white matter damage by modulating microglia toward M2 polarization *via* STAT3 pathway. Stroke (2017) 48(12):3336–46. doi: 10.1161/STROKEAHA.117.018505 PMC5728178 29114096

